# A Randomized Trial of Two Coverage Targets for Mass Treatment with Azithromycin for Trachoma

**DOI:** 10.1371/journal.pntd.0002415

**Published:** 2013-08-29

**Authors:** Sheila K. West, Robin Bailey, Beatriz Munoz, Tansy Edwards, Harran Mkocha, Charlotte Gaydos, Thomas Lietman, Travis Porco, David Mabey, Thomas C. Quinn

**Affiliations:** 1 Dana Center for Preventive Ophthalmology, Johns Hopkins University, Baltimore, Maryland, United States of America; 2 London School of Hygiene and Tropical Medicine, London, United Kingdom; 3 Kongwa Trachoma Project, Kongwa, Tanzania; 4 International Chlamydia Laboratory, Department of Infectious Diseases, Johns Hopkins University, Baltimore, Maryland, United States of America; 5 Proctor Foundation, University of California, San Francisco, San Francisco, California, United States of America; 6 Division of Intramural Research, National Institute for Allergy and Infectious Diseases (NIAID), Bethesda, Maryland, United States of America; University of California San Diego School of Medicine, United States of America

## Abstract

**Background:**

The World Health Organization recommends at least 3 annual antibiotic mass drug administrations (MDA) where the prevalence of trachoma is >10% in children ages 1–9 years, with coverage at least at 80%. However, the additional value of higher coverage targeted at children with multiple rounds is unknown.

**Trial Design:**

2×2 factorial community randomized, double blind, trial.

**Trial methods:**

32 communities with prevalence of trachoma ≥20% were randomized to: annual MDA aiming for coverage of children between 80%–90% (usual target) versus aiming for coverage>90% (enhanced target); and to: MDA for three years versus a rule of cessation of MDA early if the estimated prevalence of ocular *C. trachomatis* infection was less than 5%. The primary outcome was the community prevalence of infection with *C. trachomatis* at 36 months.

**Results:**

Over the trial's course, no community met the MDA cessation rule, so all communities had the full 3 rounds of MDA. At 36 months, there was no significant difference in the prevalence of infection, 4.0 versus 5.4 (mean adjusted difference = 1.4%, 95% CI = −1.0% to 3.8%), nor in the prevalence of trachoma, 6.1 versus 9.0 (mean adjusted difference = 2.6%, 95% CI = −0.3% to 5.3%) comparing the usual target to the enhanced target group. There was no difference if analyzed using coverage as a continuous variable.

**Conclusion:**

In communities that had pre-treatment prevalence of follicular trachoma of 20% or greater, there is no evidence that MDA can be stopped before 3 annual rounds, even with high coverage. Increasing coverage in children above 90% does not appear to confer additional benefit.

## Author Summary

The World Health Organization recommends at least 3 annual antibiotic mass drug administrations (MDA) where the prevalence of trachoma is >10% in children ages 1–9 years, with coverage at least at 80%. However, the additional value of higher coverage targeted at children with multiple rounds is unknown. We randomized 32 communities in Kongwa, Tanzania, with starting prevalence estimated at >20% to four arms: annual MDA aiming for coverage of children between 80%–90% (usual target) versus aiming for coverage>90% (enhanced target); and to: MDA for three years versus a rule of cessation of MDA early if the estimated prevalence of ocular *C. trachomatis* infection was less than 5%. After three rounds of MDA, infection with *C. trachomatis* and trachoma had declined significantly from baseline but no communities had treatment stopped. There was no difference in infection or in trachoma at three years comparing the usual coverage communities to the enhanced coverage communities. We conclude that in communities that had pre-treatment prevalence of follicular trachoma of 20% or greater, there is no evidence that MDA can be stopped before 3 annual rounds, even with high coverage. Increasing coverage in children above 90% does not appear to confer additional benefit.

## Introduction

Trachoma, caused by ocular *Chlamydia trachomatis*, is the leading infectious cause of blindness world-wide [1]. In addition to the heavy personal and societal burden inflicted by loss of vision, there are significant economic impacts as well. The 3.8 million cases of blindness and 5.3 million cases of low vision due to trachoma are estimated to diminish productivity by $2.9 billion each year [2].

A multi-faceted strategy to control all phases of trachoma has been endorsed by the World Health Organization (WHO), consisting of **S**urgery (to repair lids distorted by trachoma (trichiasis) in imminent danger of vision loss), **A**ntibiotics (mass drug (antibiotic) treatment (MDA) to reduce the community pool of infection with *Chlamydia trachomatis*), **F**ace washing (to reduce transmission from ocular and nasal secretions), and **E**nvironmental improvements (to interrupt transmission and prevent re-emergence). Children, particularly pre-school age children, in trachoma-endemic communities are the reservoir of infection and active follicular trachoma [3]–[5].

The WHO has recommended at least three years of annual MDA, with coverage targets of 80% of the population, followed by an impact survey to guide continued MDA. The indication for ongoing MDA is a prevalence of clinical follicular trachoma of ≥10% in children ages 1 to 9 years. However, there are no data on whether increasing coverage in children would result in more rapid declines in infection. Recent data from Tanzania suggest that with coverage less than 80%, more than seven years of annual mass drug administration (MDA) might be needed to achieve the target of trachoma less than 5% [6]. On the other hand, one round of MDA with high coverage in low prevalence communities in The Gambia lowered the infection rate to virtually zero although clinical signs of trachoma were still present [7]. Such data suggest that in low prevalence communities, high coverage may obviate the need for three annual rounds of MDA. In addition, relying for cessation of treatment on clinical signs of trachoma may result in unnecessary treatment of communities where infection has been eliminated and only residual disease is present. Yet, in some communities, even low rates of active trachoma are associated with the presence of infection [8]. Research on the optimal use of antibiotic for trachoma control, especially now that MDA distribution may be integrated with other neglected tropical diseases is urgently needed [9].

We hypothesized that increasing the coverage of MDA to greater than 90% as monitored in children would result in more rapid decline in infection and trachoma compared to usual coverage, between 80–90%. Where communities achieved an estimated prevalence of infection less than 5% in children ages ≤ five years (regardless of status of clinical signs), we hypothesized that the community could cease mass treatment without re-emergence. To test these hypotheses, we conducted a community-randomized trial in Kongwa Tanzania.

## Methods

### Ethics Statement

All study procedure and protocols were approved by the Johns Hopkins University Institutional Review Board and the National Institute for Medical Research in Tanzania. Written informed consent was obtained by parents on behalf of all child participants. ClinicalTrials.gov ID#NCT00792922.

### Overview

Communities were randomized 1∶1∶1∶1 in a 2×2 factorial design to two different coverage targets: 80%–90% (usual target) versus >90% (enhanced target). They were also randomized to two different annual MDA strategies: yearly mass treatment for 3 years (usual care) versus yearly mass treatment each year if warranted by the infection prevalence above 5%; otherwise, the MDA would cease for communities in this arm and the community monitored for re-emergent infection (cessation rule) [10].

Communities were randomly selected from all communities in Kongwa district based on previous research or assessment suggesting the prevalence of TF was 20% or greater. Within each community, 100 children less than five years old were randomly selected and surveyed for trachoma and infection at baseline and new samples selected every six months for 36 months. Mass drug administration was provided to each community annually, unless the community was randomized to the cessation rule arm and the prevalence of infection or trachoma fell to less than 5%. Our working definition of the cessation rule was if zero children out of 100 in the sentinel sample at 6 months or 18 months had infection, as determined by PCR, then the next round of MDA would not occur. With a sample size of 100 children, finding no infection is associated with an exact upper 95% confidence limit less than 5%.

**Figure 1:**
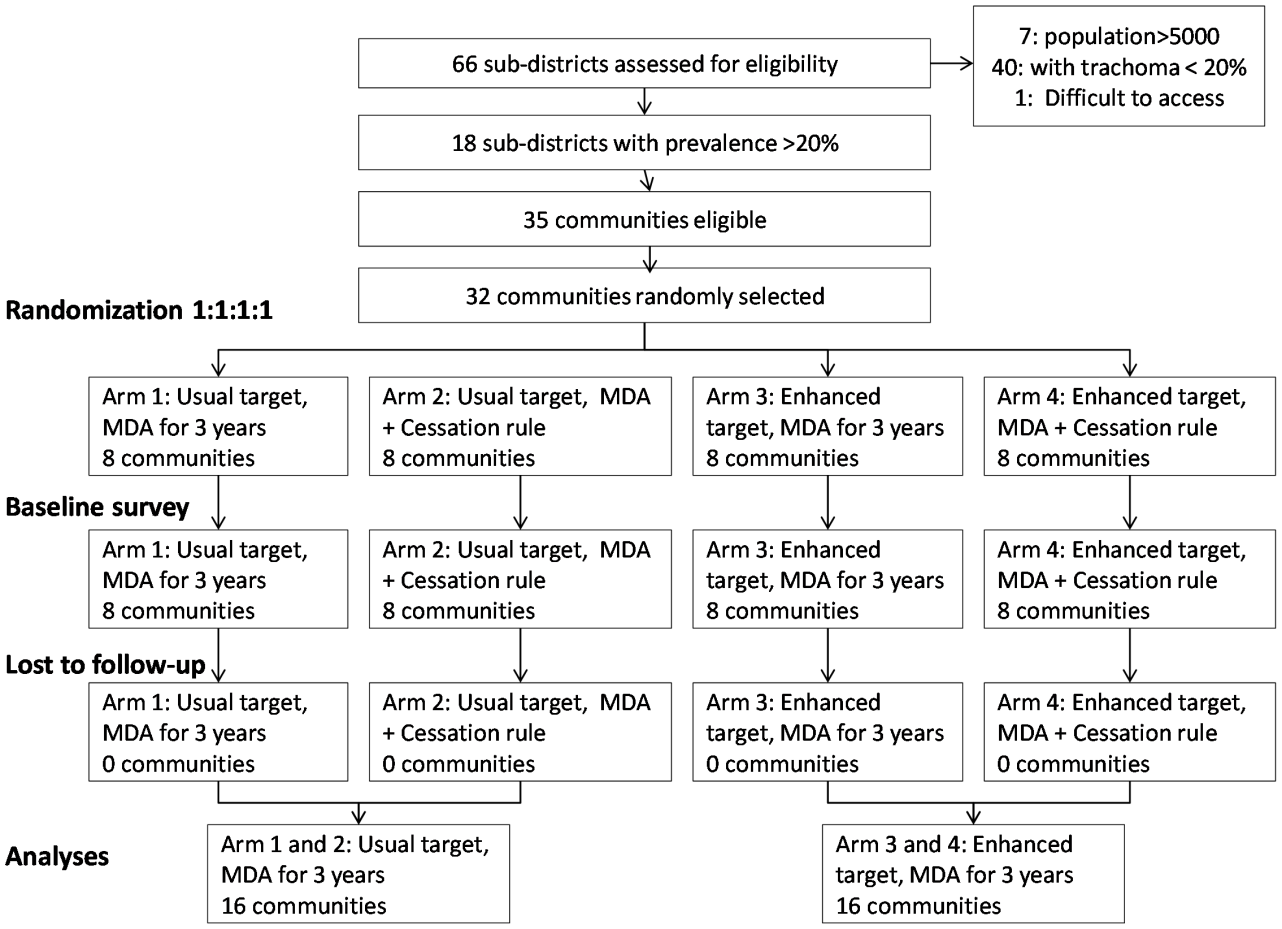
Flow of Communities in Kongwa district through the Trial.

The design of the trial is shown in Figure 1. By the 18 month survey, no community in the arms randomized to the cessation rule had achieved an estimated prevalence of infection less than 5%, so all communities proceeded to have three rounds of MDA. Thus, for the final analyses, the main effect of the stopping rule was not considered since no communities were stopped. Only the main effect for the coverage arms was analyzed.

### Communities

The eligibility criteria for communities in this trial were as follows:

1. Communities were part of the Kongwa District in Tanzania
2. If the population estimate from the last Tanzania census for the community was greater than 5,000 persons, the community was not included as these were considered urban areas.
3. Based on initial assessment the community prevalence of trachoma was estimated at 20% or higher
4. The community leadership agreed that the community could participate.

We defined communities as the smallest population level for implementation of services. Kongwa district is part of the National Trachoma Control Program for Tanzania, so all communities outside the trial were offered MDA if the prevalence of TF was >10% in children ages 0–9 years.

### Intervention

MDA consists of a single dose of azithromycin, 20 mg/kg up to 1 GM, offered to all residents of the community ages 6 months or older. Infants under age 6 months are offered topical tetracycline. MDA was carried out by a network of paid community treatment assistants (CTAs) who were trained and supervised by Kongwa Trachoma Project staff. These were the only staff that had access to the randomization assignment of the community in order to monitor coverage and plan for MDA cessation.

The census list of the community was used to monitor coverage, and as each resident presented for treatment, treatment was observed and recorded in the treatment log by the CTA. The numbers of treatment days were scheduled depending on the tally of coverage at the end of the first three days in each community. For subsequent days, the CTAs were notified in advance of persons who missed MDA, and were to go house to house to provide treatment. If, after the first 3 days of MDA, the percentage of children treated was between 80% and 90% for the one coverage arm, or above 90% for the second coverage arm, then treatment for that community stopped. If treatment was below target, then the staff supervisor scheduled subsequent days for the community, with treatment to be held for another day until coverage targets are met or all persons accounted for in the Treatment log. All persons who presented for treatment were treated, even if it meant higher than 90% coverage for communities randomized to 80–90% coverage.

Treatment verification was undertaken after each round of MDA by having a sample of five households per CTA re-visited by a supervisor. Each person in the household was queried about receiving treatment. Payment of TSH 1,000 per day for the CTA ($0.80) was contingent on achieving 80% agreement on coverage of persons in the five households.

### Outcome Measures

The primary outcome measures are the prevalence of *C. trachomatis* infection, and the prevalence of follicular trachoma, at the community level at the 36 months survey (last follow up December 2011).

### Randomization Scheme

Using a custom built SAS macro for constrained randomization, the 32 villages were randomly assigned on a 1∶1∶1∶1 to each arm of the trial. This approach reduces the likelihood of a bad randomization outcome by constraining the randomization by baseline trachoma prevalence as a co-variate [11], thus ensuring balance in each arm. The study statistician had the responsibility for generating the random assignment of communities. After the village leadership had agreed the community would participate, the census and survey completed, the random assignment was provided to the project director in Tanzania. He then informed the MDA implementation team immediately prior to MDA.

For each survey, a custom built Access program (Microsoft Office 2007) randomly selected from the most recent census a list of 110 children ages 5 years and under. A random number was assigned to each child and we used the first 110 lowest numbers. Ten children were kept in reserve in case of the unavailability of a child in the first 100 children sampled.

### Sample Size Determination

Assuming a standard deviation of 0.05 within each arm, a correlation of 0.5 between baseline and 36 month results, a figure observed from previous studies, and no interaction between factors, we estimate that a total of 32 communities provides greater than 80% power for each main effect. Neither the assumption of plausible variation in a possible interaction effect nor the assumption of a beta distribution rather than a normal distribution altered these estimates to any substantial degree. Although we estimated that between 40% and 80% of communities randomized to the cessation rule arms would cease MDA, in fact zero communities ceased MDA. Thus, for the final analyses, 16 villages were in the 80–90% coverage group and 16 villages were in the >90% coverage group.

### Masking

The survey teams who assessed trachoma were masked to the allocation of the communities in each arm, as they were never shown the allocation and communities were surveyed in no order by treatment allocation. It was theoretically possible that if a community had MDA stopped their allocation to the cessation rule would be unmasked, but none of the communities were stopped.

The laboratory at Johns Hopkins University received specimens with labels that could not be linked to persons or study arms by lab personnel, and these were processed for infection masked to intervention. The results were reported only to the statistician and data managers of her team. The community residents who participated in the survey were not told the results of the laboratory findings, since all were eligible to receive azithromycin. Thus, the infection outcomes were double masked.

### Study Methods

#### Census

In each of the 32 communities, a complete census of all households was carried out starting in May 2008 until December 2008. The census was updated yearly and new households and new persons in existing households were recorded, as well as households and persons who no longer resided in the community were noted.

#### Surveys

Details of the surveys are described elsewhere [10], but summarized as follows:

The 100 sentinel children presented for examination at a central site. Written informed consent was obtained from the guardian prior to the start of the examination. The trachoma grader, lid flipper, and laboratory person were all gloved and changed gloves between subjects to avoid field contamination. Both eyelids of the child were everted and the tarsal conjunctiva graded for signs of clinical trachoma, using the WHO simplified grading scheme [12]. On a random sample of sentinel children, ocular photographs of the right eye were taken to determine drift in grading over time. A swab to test for infection was taken of the right eye, using a Dacron swab, stored dry and frozen until shipped to the International Chlamydia laboratory at Johns Hopkins. A 5% sample of “air” control swabs were also taken to test for field and laboratory contamination.

The specimens were processed using the Amplicor kit (Roche Molecular systems, Pleasanton, CA) according to strict protocol, outlined in the manufacturer's kit directions. Each swab was eluted by vortexing in Amplicor CT/NG lysis buffer in polypropylene tubes, and then Amplicor specimen diluent was added. Two *C. Trachomatis* positive and two *C. trachomatis* negative processing controls were run with each batch of specimens. Samples whose values in valid runs were ≥0.8 A_450_ were counted as positive, and samples less than 0.2 A_450_ were considered negative. Samples for which the result were equivocal (≥0,2, <0.8) were tested again; if equivocal twice, they were left as equivocal and reported as not positive in the analyses as no run equaled 0.8 or greater. Less than 0.1% of specimens were equivocal. The laboratory personnel were masked to community and treatment assignment.

Baseline, 12 month, and 24 month surveys were carried out prior to annual MDA.

#### Quality control

Trachoma assessment was monitored over time using the field photographs, at least 50 per grader per visit. These were graded after each survey by a senior grader (RB) masked to the field grades and to the grades of the images by the field graders. Agreement was assessed using Kappa statistic, and if agreement fell below 0.6 for any survey, re-standardization was instituted to bring agreement to acceptable levels. At no survey was agreement less than 0.6

Field and laboratory contamination was monitored over time using the field “air” controls, as well as internal laboratory controls for each run. If any field control was positive, then immediate investigation was undertaken to determine the source of contamination, and specimens re-run if needed to confirm results. At the baseline survey, the laboratory experienced contamination in the initial processing of the specimens, and 76 specimens (23, 43, and 10 in each of three communities) out of 3200 could not be used. There were no further losses due to contamination in the study.

Data quality, results of monitoring for quality of trachoma assessment and laboratory assessment of infection, were presented to a yearly meeting of the Data and Safety Monitoring Committee.

#### Statistical analyses

Because no community in the “stopping rule” arm reached the pre-specified rule for ceasing MDA, the analytic plan was based on the main effect of two coverage arms, as follows:

The primary analyses were conducted on the intention-to-treat principle (ITT); therefore, communities were analyzed according to their coverage randomization assignment regardless of their actual coverage level. We pre-specified that if there was no difference in the ITT analyses, we would undertake analyses of actual coverage and the relationship to infection and trachoma at 36 months.

We compared baseline community and other characteristics that may be related to the prevalence of infection/follicular trachoma prior to conducting further analyses, and any variables unevenly distributed among groups was included in all multivariate models to control for potential residual confounding. Population characteristics were summarized at community level and then summarized by group. The mean (SD) for each arm is presented for each characteristic. The Kruskal-Wallis test was used for comparison.

This trial had a pre-specified outcome of the prevalence at 36 months only, because usual program practice is to evaluate the outcomes at 3 years post-baseline and it was felt that the difference in coverage levels would be seen by three years. We modeled the community-level prevalence at 3-years post first mass treatment on a square root transformed scale. Ordinary least squares (OLS) linear regression was used to model the square-root transformed prevalence including independent variables for coverage arm and baseline prevalence of infection/follicular trachoma.

We calculated the adjusted mean difference in prevalence of infection/follicular trachoma by treatment arm at 36 months as follows: 1) For each community, using the baseline observed prevalence, treatment arm, and the parameters estimated from the square root transformed model described above, we estimated the predicted prevalence. 2) For each arm we average the estimated prevalences; 3) The difference in the adjusted mean prevalence for enhanced arm and the standard arm was then calculated. 4) In order to derive the confidence intervals for the adjusted difference, we repeated Steps 1 to 4 for 1000 bootstrap samples; 5) The median of the adjusted mean differences and corresponding 2.5% and 97.5% percentiles were reported.

## Results

A comparison of baseline characteristics of each of the 4 groups of communities showed no imbalances in population size, percentage of households with no latrine, percentage more than 30 minutes from water, or average education of head of household. Similar prevalence of follicular trachoma and *C. trachomatis* infection were observed (Table 1). For the rest of the analyses, we show only the coverage arms because none of the communities stopped MDA during the study (Figure 1)

**Table 1:** Baseline characteristics by randomization group.

Characteristic	Statistic	Allocation (8 communities per arm)				p-value Kruskal-Wallis
		80–90% coverage 3 annual MDA	>90% coverage 3 annual MDA	80–90% coverage Cessation rule	>90% coverage Cessation rule	
Population size	Mean (SD)	1456 (243)	1316 (465)	1379 (481)	1678 (472)	0.42
Household head years of education:	Mean (SD)	3.4 (0.7)	3.2 (1.0)	3.2 (1.0)	3.2 (0.9)	0.79
% houses >30 minutes from water	Mean (SD)	67.2 (28.0)	80.9 (24.2)	84.1 (14.9)	71.9 (26.3)	0.53
% houses with latrine	Mean (SD)	72.0 (10.8)	62.9 (23.4)	62.2 (10.7)	62.0 (20.3)	0.58
Prevalence of TF in children 0–<5 years	Mean (SD)	30.3 (13.5)	30.7 (16.3)	30.5 (10.4)	31.1 (9.5)	0.99
Prevalence of *C. trachomatis* in children 0–<5 years	Mean (SD)	17.8 (10.3)	24.6 (12.4)	22.4 (23.3)	23.0 (11.2)	0.55

Data are summary statistics generated from community level summary measures.

The treatment coverage at each of the three rounds of mass drug administration was consistently greater in the enhanced target group compared to the usual target group (Table 2). The average difference between the two groups in terms of coverage was never more than 6% (third round of mass treatment). All of the communities in the usual target arm achieved coverage above 80% in children under age ten years in each of the three rounds. Only at the one year treatment round did one village in the enhanced target group have coverage below 90%.

**Table 2:** Treatment coverage in children under 10 years of age by randomization group.

Time	Statistic	Allocation (16 communities per arm)		p-value Wilcoxon
		80–90%	>90%l	
Baseline	Mean ±SD	90.8±5.4	96.6±2.3	0.001
One Year	Mean ± SD	87.7±5.6	92.5±4.0	0.02
Two Years	Mean ± SD	86.7±4.3	92. 7±2.5	0.001

**Figure 2:**
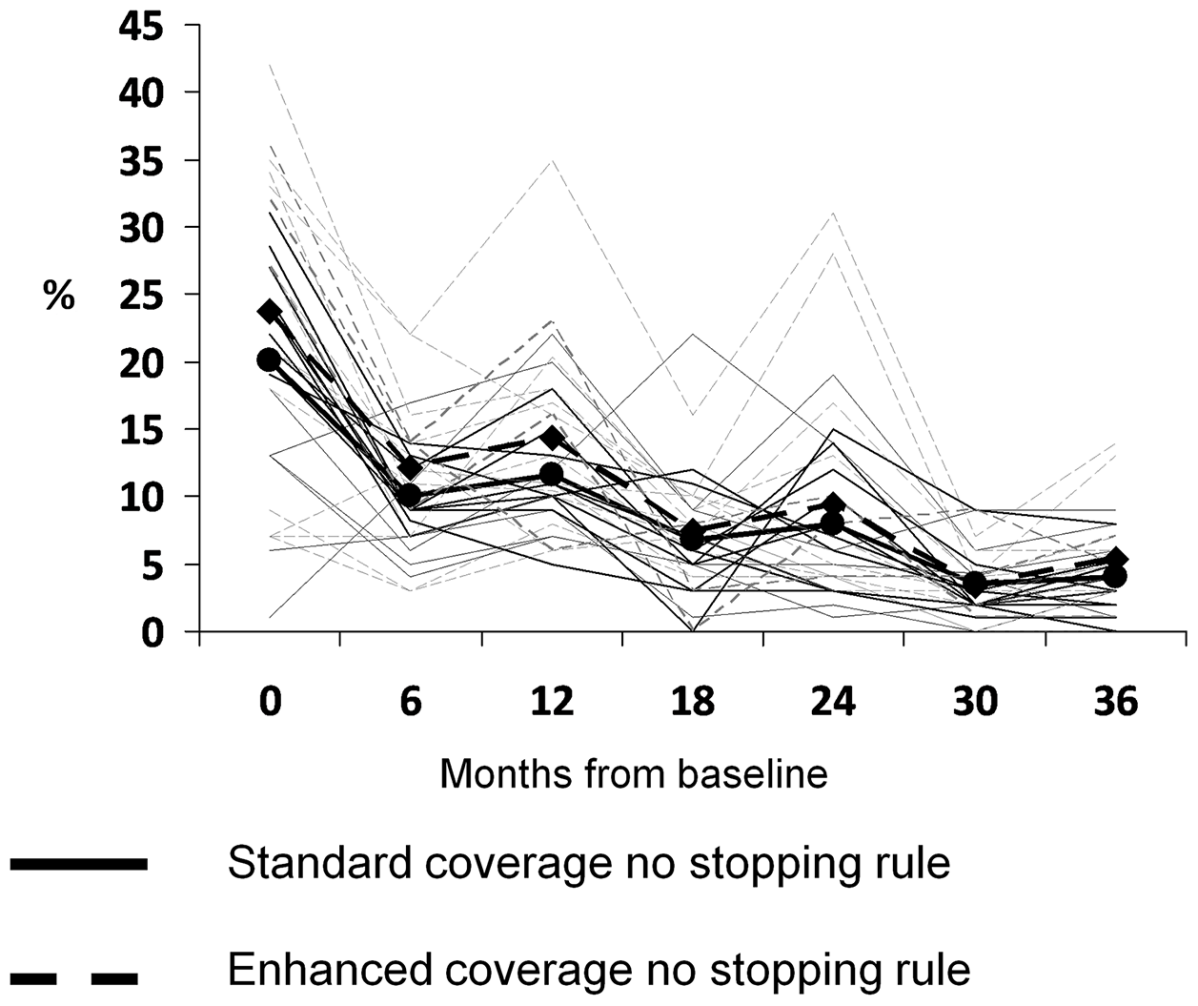
Prevalence of *C.* **trachomatis**** **Infection over time by Coverage Arm.** ****

The baseline prevalence of infection with *C. trachomatis* was not different between the two coverage groups, 20.1% and 23.8% respectively (Figure 2). There was a decline in infection from baseline to 36 months in both the usual target group and the enhanced coverage group. At 36 months (one year after the third MDA), the prevalence of infection was 4.0% in the usual target group and 5.4% in the enhanced target group, with an adjusted difference of 1.4% (95% Confidence Interval (CI) = −1.0% to 3.8%).

**Figure 3:**
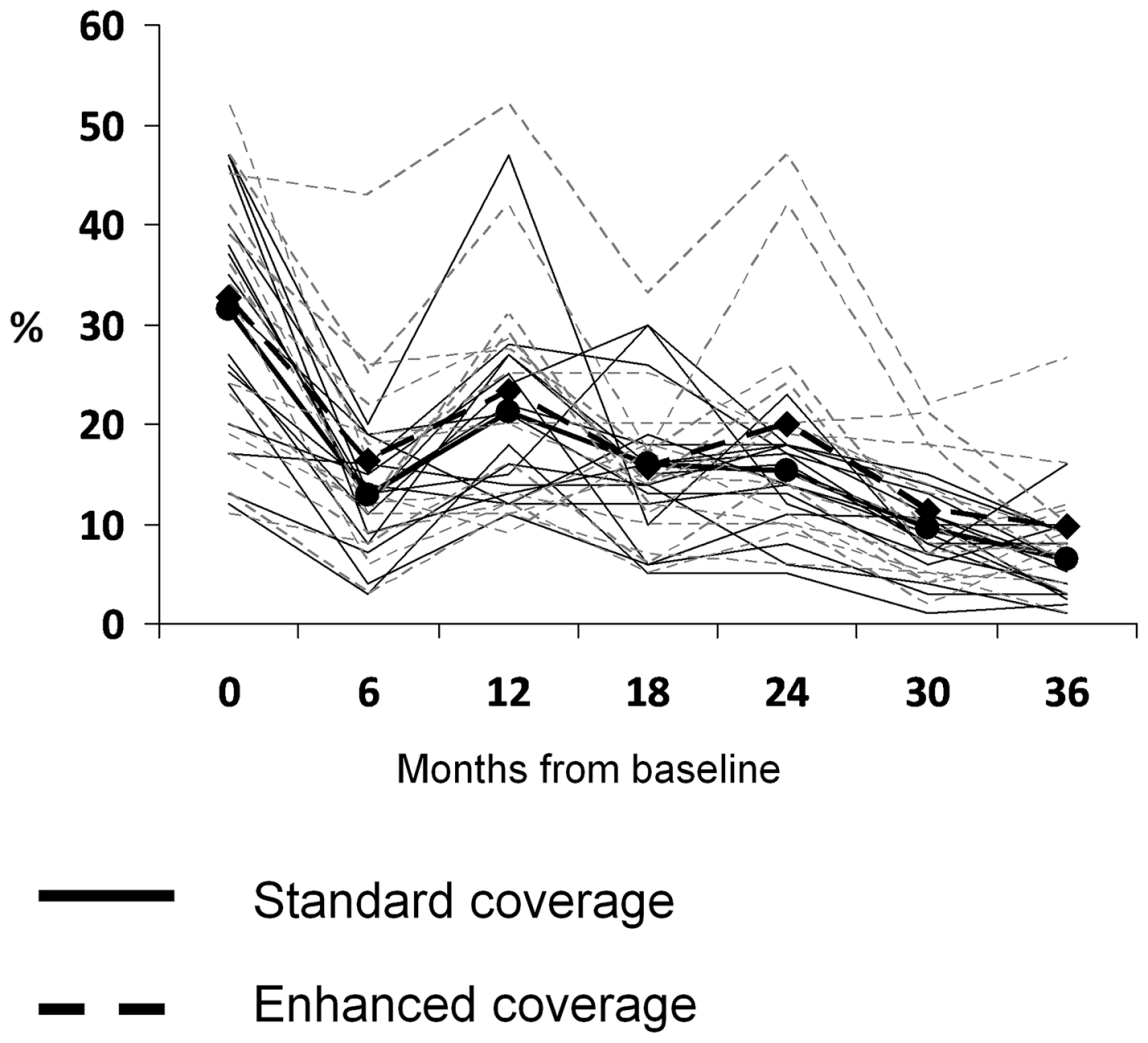
Prevalence of Follicular Trachoma (TF) over time by Coverage Arm.

The prevalence of follicular trachoma by treatment group showed a similar patter, with no difference between groups at baseline, 30.4% and 30.9% in the usual target and enhanced target group respectively (Figure 3). By 36 months, the trachoma prevalence had fallen to 6.1% and 9.0% respectively, an adjusted difference of 2.6% with 95% CI = −.3% to 5.3%.

Separate models of infection and trachoma at 36 months, adjusted for baseline prevalence, showed no effect of being in the enhanced target arm (Table 3). The prevalence of trachoma at baseline predicted the prevalence of trachoma at 36 months, but the prevalence of infection at baseline was not predictive of infection at 36 months.

**Table 3:** Impact of enhanced target coverage on prevalence* of *C. trachomatis* infection and follicular trachoma at 36 months.

Variable	Parameter Estimate (β)	95% Confidence Intervals
***Predicting infection***		
Enhanced coverage group	0.034	−0.028, 0.096
Baseline *C. trachomatis* prevalence (square root)	−0.024	−0.276, 0.228
***Predicting clinical trachoma***		
Enhanced coverage group	0.050	−0.007, 0.106
Baseline TF prevalence (square root)	0.357	0.107, 0.608

* Squared root transformation.

When analyzed as actual coverage, there was still no evidence that increasing coverage in children resulted in lower infection or trachoma prevalence at 36 months (Table 4). If the variable of average percentage coverage over the three rounds was replaced with a variable of percentage coverage at the last round of mass treatment, there was still no evidence for a significant decrease in infection or trachoma with increasing coverage at 36 months (data not shown).

**Table 4:** Impact of actual coverage on prevalence of *C. trachomatis* and follicular trachoma at 36 months.

Variable	Parameter Estimate (β)	95% Confidence Intervals
***Predicting Infection***		
Average Coverage (per % increase)	−0.0006	−0.0088, 0.0077
Baseline *C. trachomatis* prevalence (square root)	0.006	−0.278, 0.290
***Predicting clinical Trachoma***		
Average coverage (per % increase)	0.0038	−0.0032, 0.0108
Baseline TF prevalence (square root)	0.336	0.0725, 0.5985

There were no serious adverse events reported in either arm.

## Discussion

This community based, randomized trial had two major findings: first, there was no evidence of benefit to increasing mass drug administration coverage of children ages under ten years above 90%, compared to targeting coverage between 80–90%. The analyses repeated on actual coverage found no benefit per unit increase in coverage either. Second, that if the baseline prevalence of trachoma in communities are estimated at 20% or greater, two years of annual MDA were insufficient to decrease infection in any community below an estimate of 5% and at least three annual rounds will be necessary even with high coverage.

There may be at least two possible reasons for the first finding. With coverage this high in children, there were very few children who were not treated at least once after three MDAs. In a study in these communities of persistent non- participation in MDA, Ssemanda et al found that only 2% of households contained children who did not participate in 2 MDAs [13]. By the third round of MDA, there may be little benefit to the extra effort required to achieve over 90% coverage. This supposition has supporting evidence with the rapid fall in infection by 36 months in both arms of the trial. Trachoma at baseline in these communities averaged 30%, with 22% infection. By three years and three rounds of MDA, infection averaged 4.7% and trachoma fell to an average of 7.6%. The trajectory suggests that just a few more rounds of MDA would be needed to decrease trachoma below 5% and even eliminate infection, fewer annual rounds than suggested by other work in Tanzania where coverage was estimated at less than 75% [6].

Another possible reason is that the difference in coverage in the enhanced target versus the usual target groups was relative small. The coverage in children in the usual target group was high, 90%, 88%, and 87% respectively for the three rounds. While some communities were lower, no community had MDA coverage in children below 80%, by design. The enhanced target group had significantly higher coverage, but the largest difference was in the third round with average coverage of 93% compared to the usual care group with average coverage of 87%. However, even adjusting for slightly higher rates of infection in the enhanced coverage group, there was no survey where the enhanced coverage group had a point estimate that was lower infection or trachoma compared to the usual coverage group. When the analyses were repeated using actual coverage in the communities, where the range was greater, there was no apparent effect of increasing coverage on infection or trachoma. The data appear to be more consistent with an absence of an effect of enhanced coverage than failure to detect a difference.

It is unlikely that the intervention was carried out improperly in either arm of the trial. Treatment verification consistently showed high agreement between recorded treatment and personal history of recipients. Trachoma and infection fell over time consistent with good coverage in children as reported from studies elsewhere in other high prevalence villages [14], [15].

The fact that we did not stop MDA in any of the communities randomized to the cessation rule, even with high coverage, provides support for the WHO guideline that suggest 3 rounds of MDA before re-assessing the impact, at least for communities with baseline prevalence above 20%. We had expected that with high coverage, communities would reach our pre-specified target of zero cases of infection in 100 sentinel children before the third round of MDA. This did not happen when the starting prevalences of infection in the communities were, on average, 22%. Our experience was different than the experience with high coverage in small communities in Ethiopia, where after a single round of high coverage, infection fell from 50% to less than 5% at 6 months and after a second round at 6 months, disappeared [15]. Other studies in The Gambia and in a low prevalence community in Tanzania reported that one to two annual rounds was sufficient to eliminate infection [7], [16], but these communities started with low levels of infection. A recent study from Ethiopia noted that annual treatment with high coverage reduced infection from 42% in children ages 0–9 years to 1.9% after four rounds of annual MDA, which is more consistent with the trajectory of our observed decline [17]. In that study, 5 of the 12 communities had no infection at 36 months, but with on average only 50 sentinel children per community, the possibility of infection as high as 5% could not be ruled out.

In summary, a community randomized clinical trial comparing high coverage (80–90%) in children with antibiotic for trachoma to very high coverage (>90%) found no difference in infection or trachoma rates after three annual rounds of MDA. The results suggest that aiming for at least 80% coverage of children in trachoma endemic communities is reasonable, and there is no advantage to expending resources to push for even higher coverage. When treating communities with 20% or greater prevalence of trachoma at baseline, at least 3 rounds of MDA will be needed before infection drops confidently below 5%.

## Supporting Information

Checklist S1 Consort checklist.
(DOC)

Protocol S1 Trial protocol.
(DOC)
