# Supplementary material for: A Randomized Trial of Two Coverage Targets for Mass Treatment with Azithromycin for Trachoma
Source: PLoS Negl Trop Dis. 2013 Aug 29;7(8):e2415. doi: 10.1371/journal.pntd.0002415 (PMC3757067; doi:10.1371/journal.pntd.0002415)
Supplement: Protocol S1 — Trial protocol. (DOC) [file pntd.0002415.s002.doc]

Version

10

# Partnership for the Rapid

# Elimination of Trachoma (PRET)

# Manual of Operations


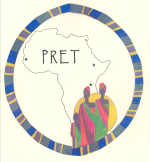


© Johns Hopkins Medical Institutions

600 N Wolfe St Baltimore MD, 21205

Tel: (410) 955-2606

FAX: (410) 955-0096

Table of Contents

[PRET Manual of Operations: Version Updates 7](#__RefHeading___Toc282515976)

[Chapter 1: Overview 9](#__RefHeading___Toc282515977)

[1.1 Overview 9](#__RefHeading___Toc282515978)

[1.2 Research Questions 10](#__RefHeading___Toc282515979)

[1.3 Overall Design 11](#__RefHeading___Toc282515980)

[1.4 PRET Ziada 12](#__RefHeading___Toc282515981)

[Chapter 2: Background and Significance 13](#__RefHeading___Toc282515982)

[2.1 Trachoma as a Public Health Problem 13](#__RefHeading___Toc282515983)

[2.2 Anticipated Impact 14](#__RefHeading___Toc282515984)

[Chapter 3: Countries and Populations 15](#__RefHeading___Toc282515985)

[3.1 Niger 15](#__RefHeading___Toc282515986)

[3.2 Tanzania 15](#__RefHeading___Toc282515987)

[3.3 The Gambia 16](#__RefHeading___Toc282515988)

[Chapter 4: Randomization Arms and Sample Size 17](#__RefHeading___Toc282515989)

[4.1 Overview 17](#__RefHeading___Toc282515990)

[4.2 Randomization Arms 17](#__RefHeading___Toc282515991)

[4.3 Communities and Sentinel Children 19](#__RefHeading___Toc282515992)

[4.3.1 Details on Randomization for Niger 20](#__RefHeading___Toc282515993)

[4.3.2 Details on Randomization for Tanzania 21](#__RefHeading___Toc282515994)

[4.3.3 Details on Randomization for The Gambia 22](#__RefHeading___Toc282515995)

[4.4 Sample Size 23](#__RefHeading___Toc282515996)

[4.4.1 Villages 23](#__RefHeading___Toc282515997)

[4.4.2 Children 26](#__RefHeading___Toc282515998)

[Chapter 5: Eligibility, Consent and Enrollment 27](#__RefHeading___Toc282515999)

[5.1 Eligible Communities 27](#__RefHeading___Toc282516000)

[5.2 Eligible Children 28](#__RefHeading___Toc282516001)

[5.3 Consent Procedures 28](#__RefHeading___Toc282516002)

[5.3.1 Niger 28](#__RefHeading___Toc282516003)

[5.3.2 Tanzania 29](#__RefHeading___Toc282516004)

[5.3.3 The Gambia 31](#__RefHeading___Toc282516005)

[5.4 Risks and Efforts to Minimize Risk 31](#__RefHeading___Toc282516006)

[Chapter 6: Procedures: Census of communities 33](#__RefHeading___Toc282516007)

[6.1 Overview 33](#__RefHeading___Toc282516008)

[6.2 Baseline Census 33](#__RefHeading___Toc282516009)

[6.3 Interim Census 34](#__RefHeading___Toc282516010)

[Chapter 7: Procedures: Study Visits 35](#__RefHeading___Toc282516011)

[7.1 Overview 35](#__RefHeading___Toc282516012)

[7.2 Baseline Visit 35](#__RefHeading___Toc282516013)

[7.2.1 Trachoma Assessment 36](#__RefHeading___Toc282516014)

[7.2.2 Laboratory Procedures 37](#__RefHeading___Toc282516015)

[7.2.3 Shipping and Storage of Samples 39](#__RefHeading___Toc282516016)

[7.2.4 Laboratory Processing Procedures 40](#__RefHeading___Toc282516017)

[7.2.5 Photograph Grading 41](#__RefHeading___Toc282516018)

[7.2.6 Photography Protocol 41](#__RefHeading___Toc282516019)

[7.2.6 Follow-up of Children 43](#__RefHeading___Toc282516020)

[7.3 Forms and Swab Management for Visits 43](#__RefHeading___Toc282516021)

[7.4 Missed Visits 44](#__RefHeading___Toc282516022)

[7.5 Follow-Up Visits 44](#__RefHeading___Toc282516023)

[7.6 Sample Study Visit Schedule 44](#__RefHeading___Toc282516024)

[Chapter 8: Intervention: Mass Treatment Implementation 47](#__RefHeading___Toc282516025)

[8.1 Overview: Treatment with Azithromycin 47](#__RefHeading___Toc282516026)

[8.2 Niger 47](#__RefHeading___Toc282516027)

[8.2.1 Six monthly Mass Treatment (Niger) 48](#__RefHeading___Toc282516028)

[8.3 Tanzania 49](#__RefHeading___Toc282516029)

[8.4 The Gambia 50](#__RefHeading___Toc282516030)

[8.5 Monitoring Mass Treatment Coverage 52](#__RefHeading___Toc282516031)

[8.5.1 Mass Treatment Verification Procedure 52](#__RefHeading___Toc282516032)

[8.6. Mass Treatment and Stopping Rule Implementation 53](#__RefHeading___Toc282516033)

[8.7 Masking Intervention 54](#__RefHeading___Toc282516034)

[Chapter 9: Cost Effectiveness Data Collection 55](#__RefHeading___Toc282516035)

[9.1 Overview 55](#__RefHeading___Toc282516036)

[9.2 Cost Data Collection 55](#__RefHeading___Toc282516037)

[Chapter 10: Study Instruments 56](#__RefHeading___Toc282516038)

[10.1 Overview 56](#__RefHeading___Toc282516039)

[10.2 Census Forms 56](#__RefHeading___Toc282516040)

[10.2.1 Baseline Census Form (Form 1) 56](#__RefHeading___Toc282516041)

[10.2.2 Census Update Forms 59](#__RefHeading___Toc282516042)

[10.2.3 Master List of Eligible Children for Survey (Form 4) 61](#__RefHeading___Toc282516043)

[10.3 Ocular Form (Form 5) 61](#__RefHeading___Toc282516044)

[10.4 Photo Log Book (Form 6) 63](#__RefHeading___Toc282516045)

[10.5 Ocular Specimen Shipping List (Form 7) 63](#__RefHeading___Toc282516046)

[10.6 Mass Treatment Log Book (Form 8) 63](#__RefHeading___Toc282516047)

[10.7 Stopping Rule: Decision Report Form (Form 9) 64](#__RefHeading___Toc282516048)

[10.8 Cost Forms 64](#__RefHeading___Toc282516049)

[Chapter 11: Training and Certification 68](#__RefHeading___Toc282516050)

[11.1 Trachoma Grading 68](#__RefHeading___Toc282516051)

[11.2 Laboratory Specimen Handling 69](#__RefHeading___Toc282516052)

[11.3 Photography 69](#__RefHeading___Toc282516053)

[11.4 Study Forms 69](#__RefHeading___Toc282516054)

[11.5 Data entry 70](#__RefHeading___Toc282516055)

[Chapter 12: Data Management and Routine Reports 71](#__RefHeading___Toc282516056)

[12.1 Overview 71](#__RefHeading___Toc282516057)

[12.2 Niger 71](#__RefHeading___Toc282516058)

[12.2.1 Study Forms Preparation 71](#__RefHeading___Toc282516059)

[12.2.2 Daily Data Management Activities in Field 72](#__RefHeading___Toc282516060)

[12.2.3 Data entry 72](#__RefHeading___Toc282516061)

[12.2.4 Preparation of Data for Transfer to UCSF 72](#__RefHeading___Toc282516062)

[12.2.5 Preparation of data for transfer to LSHTM 73](#__RefHeading___Toc282516063)

[12.2.6 Data Editing at the Coordinating Center 73](#__RefHeading___Toc282516064)

[12.2.7 Data management process 73](#__RefHeading___Toc282516065)

[12.2.8 Periodic reports 74](#__RefHeading___Toc282516066)

[12.2.9 PCR result reports 74](#__RefHeading___Toc282516067)

[12.2.10 Data Storage 74](#__RefHeading___Toc282516068)

[12.3 Tanzania 74](#__RefHeading___Toc282516069)

[12.3.1 Overview 74](#__RefHeading___Toc282516070)

[12.3.2 Daily Data Management Activities in Field 74](#__RefHeading___Toc282516071)

[12.3.3 Data Entry 75](#__RefHeading___Toc282516072)

[12.3.4 Preparation of Data for Transfer to JHU Data Coordinating Center 76](#__RefHeading___Toc282516073)

[12.3.5 Data Editing 77](#__RefHeading___Toc282516074)

[12.3.6 Generation of Follow-up Lists 77](#__RefHeading___Toc282516075)

[12.3.7 Periodic Reports 78](#__RefHeading___Toc282516076)

[12.3.8 Data Storage 78](#__RefHeading___Toc282516077)

[12.3.9 Preparation of data for transfer to LSHTM, UCSF 79](#__RefHeading___Toc282516078)

[12.4 The Gambia 79](#__RefHeading___Toc282516079)

[12.4.1 Study Forms Preparation 79](#__RefHeading___Toc282516080)

[12.4.2 Daily Data Management Activities in Field 80](#__RefHeading___Toc282516081)

[12.4.3 Data Entry 80](#__RefHeading___Toc282516082)

[12.4.4 Preparation of Data for Transfer to LSHTM 80](#__RefHeading___Toc282516083)

[Chapter 13: Quality Assurance Policies and Procedures 82](#__RefHeading___Toc282516084)

[13.1 Overview 82](#__RefHeading___Toc282516085)

[13.2 General Guidelines for Form Completion 83](#__RefHeading___Toc282516086)

[13.3 Missing Data on Study Forms 83](#__RefHeading___Toc282516087)

[13.4 Changing Responses on Study Forms 84](#__RefHeading___Toc282516088)

[13.5 Oversight 84](#__RefHeading___Toc282516089)

[13.6 Adherence to Procedures 84](#__RefHeading___Toc282516090)

[13.7 Quality Assurance for Field Trachoma Grading 84](#__RefHeading___Toc282516091)

[13.8 Quality Assurance for Specimens 85](#__RefHeading___Toc282516092)

[Chapter 14: Adverse Events 87](#__RefHeading___Toc282516093)

[Chapter 15: Ancillary Studies 88](#__RefHeading___Toc282516094)

[15.1 Risk of Re-emergence: Pediatric Longitudinal Study 88](#__RefHeading___Toc282516095)

[15.2 Modeling Trajectory of Disease Elimination: Public Health Planning Study 88](#__RefHeading___Toc282516096)

[15.3 Ancillary Benefits of Mass Treatment with Azithromycin 89](#__RefHeading___Toc282516097)

[15.4 Cost Effectiveness Study 89](#__RefHeading___Toc282516098)

[15.5 Laboratory Screening Study 89](#__RefHeading___Toc282516099)

[Chapter 16: Data Analyses Plan 90](#__RefHeading___Toc282516100)

[16.1. Primary Outcome Analyses 90](#__RefHeading___Toc282516101)

[16.1.1 Community-level Analysis 90](#__RefHeading___Toc282516102)

[16.1.2 Child-level Analysis 91](#__RefHeading___Toc282516103)

[16.2 Additional Analyses 92](#__RefHeading___Toc282516104)

[16.2.1 Cost Effectiveness Analyses 92](#__RefHeading___Toc282516105)

[16.2.2 Model of Trachoma and Infection Elimination 93](#__RefHeading___Toc282516106)

[16.2.3 Re-emergence: Longitudinal Study of Cohorts of Children 94](#__RefHeading___Toc282516107)

[16.2.4 Specific Analyses for the Niger site 95](#__RefHeading___Toc282516108)

[Chapter 17: Responsibilities of PRET Partners 97](#__RefHeading___Toc282516109)

[17.1 Overview 97](#__RefHeading___Toc282516110)

[17.2 PRET Executive Committee 97](#__RefHeading___Toc282516111)

[17.3 Study Chairman’s Office 98](#__RefHeading___Toc282516112)

[17.4 Clinical Centers: Niger, Tanzania, and The Gambia 98](#__RefHeading___Toc282516113)

[17.5 Data Coordination Centers 99](#__RefHeading___Toc282516114)

[17.5.1 The Data Coordination Center 99](#__RefHeading___Toc282516115)

[17.5.2 Trachoma Grading Quality Assurance Center 101](#__RefHeading___Toc282516116)

[17.5.3 Laboratory Quality Assurance Center 102](#__RefHeading___Toc282516117)

[Chapter 18: Data and Safety Monitoring Committee and Contact Information 104](#__RefHeading___Toc282516118)

[18.1 Charter of Data and Safety Monitoring Committee 104](#__RefHeading___Toc282516119)

[18.2 Terms of Reference 105](#__RefHeading___Toc282516120)

[18.3 DSMC Key Responsibilities 106](#__RefHeading___Toc282516121)

[18.4 DSMC Membership 106](#__RefHeading___Toc282516122)

[18.4.1 Conflicts of Interest 106](#__RefHeading___Toc282516123)

[18.4.2 Relationships 107](#__RefHeading___Toc282516124)

[18.5 DSMC Meetings 107](#__RefHeading___Toc282516125)

[18.5.1 Protocol Changes and Ancillary Studies 108](#__RefHeading___Toc282516126)

[18.5.2 Recommendations 108](#__RefHeading___Toc282516127)

[Chapter 19: Timeline 110](#__RefHeading___Toc282516128)

[Figure 4: PRET Timeline - All Locations 110](#__RefHeading___Toc282516129)

[Figure 5: PRET Timeline - Niger 111](#__RefHeading___Toc282516130)

[Figure 6: PRET Timeline - Tanzania 112](#__RefHeading___Toc282516131)

[Figure 7: PRET Timeline - The Gambia 113](#__RefHeading___Toc282516132)

[Chapter 20: Overall Project 114](#__RefHeading___Toc282516133)

[Figure 8: Program for the Rapid Elimination of Trachoma Overview 114](#__RefHeading___Toc282516134)

[Appendix 1: General Study Forms 115](#__RefHeading___Toc282516135)

[Appendix 2: Informed Consent Forms 115](#__RefHeading___Toc282516136)

[Appendix 3: DSMC Tables 115](#__RefHeading___Toc282516137)

# PRET Manual of Operations: Version Updates

|  | | |
| --- | --- | --- |
| Version | Date | Modifications |
| 2 | February 2008 | - Changes sample size and augmented sampling description - Table of contents as separate document |
| 3 | October 30, 2008 | - Introduced automatic table of contents - Changed the format of the entire document (e.g. added concrete chapter delineations, page numbers) - Created title page - Updated table of contents |
| 4 | October 31, 2008 | - Added version, header, and footer - Grammatical revisions to chapters 1 and 2 - Added titles for tables in the randomization arm chapter - Changed the order of countries to maintain consistency - Replaced follow-up with follow-up - Verified correct numbering of forms - Added additional chapter for project overview diagram - Updated references and table of contents |
| 5 | November 7, 2008 | - Developed and added the following tables: Chapter 4 - Table. Treatment coverage and frequency randomization arms by country, Figure 4.3.1. Randomization and survey sampling strategy for Ethiopia, Figure 4.3.2. Randomization and survey sampling strategy for Tanzania, Figure 4.3.3. Randomization and survey sampling strategy for The Gambia, Table 7.5.1: Visit schedule for Tanzania, Table 7.5.2. Visit schedule for Ethiopia, 18.2 Table. DSMC Contact Information, Table. PRET Trial of Azithromycin for Trachoma Control, Figure. Program for the Rapid Elimination of Trachoma Overview - Updated table of contents |
| 6 | November 20, 2008 | - Created and inserted four timelines: 1) All locations timeline, 2) Ethiopian timeline, 3)Tanzanian timeline and 4) The Gambian timeline and added a new Project flowchart - Made modifications to the project overview - Updated references and table of contents |
| 7 | November 26, 2008 | - Inserted manual version control table - UCSF updates for Ethiopia |
| 8 | December 22, 2008 | - UCSF photo procedure update |
| 9 | January 21, 2010 | - Addition of PRET Ziada villages |
| 10 | July 28, 2010 | - Global search/replace of Ethiopia with Niger including revised site-specific protocols |

Future versions to include:

- Study forms
- DSMC reporting templates

# **Chapter 1: Overview**

Chapter

1

_______________________________________________________________________

## 1.1 Overview

The goal of this community-based randomized clinical trial is to test hypotheses about the impact on trachoma and ocular *C. trachomatis* infection of varying frequency and coverage of mass azithromycin use in communities. This trial will add to the evidence base to improve the implementation of the antibiotic component of the SAFE (Surgery/Antibiotics/ Face washing/Environmental change) strategy for trachoma control. Through the PRET partnership, we will address significant trachoma program barriers: slow decline in trachoma in these communities and re-emergence of infection following mass treatment.

Our proposal will study trachoma and ocular *C. trachomatis* infection under two alternatives for antibiotic coverage, and three alternatives for frequency of mass treatment in three program settings; first, where the disease and infection is hypoendemic (The Gambia); second, where the disease and infection were formerly hyperendemic but have declined over eight years to mesoendemic levels (Tanzania); third, where disease and infection were formerly hyperendemic but have had a lapse in programmatic activities (Niger).

The overall objective is to determine optimal strategies for elimination of trachoma and infection under differing mass treatment coverages and frequencies in areas of diverse endemicities. We have five specific objectives to meet this overall objective:

- To determine the effective coverage (80% to 90% versus above 90% in children) of mass azithromycin treatment necessary for different trachoma endemicities that will lead to rapid reduction of infection to less than 5%.
- To establish if mass azithromycin administration every six months in children can reduce infection in the entire community, and would be an effective control strategy in practice. In other words, do children act as a core group in the transmission of trachoma?
- To evaluate the potential increased effectiveness, from the trachoma control program perspective, of a laboratory-based strategy to supplement the World Health Organization (WHO) clinical strategy to identify communities where antibiotic intervention may not be further indicated, and communities can be “graduated” from mass treatment.
- To assess the effect on re-emergence of *graduating* communities that have evidence of low rates of infection, or meet World Health Organization (WHO) definition of low disease prevalence.

To meet the overall objective, then, we have two major activities: (1) Conduct clinical trials of communities in three countries, representing different baseline endemicities, of alternative coverages and frequencies of administration of mass antibiotic treatment; (2) Determine the cost-effectiveness of these different strategies from program perspective. In ancillary studies, we will determine risk factors for re-emergence, and use the data to model the effect of these alternatives to establish optimal approaches to a steep trajectory of decline in trachoma and infection.

## 1.2 Research Questions

*1) COVERAGE: What are the differences at three years post-baseline in rates of trachoma and ocular C. trachomatis infection in villages randomized to mass antibiotic coverage of 80% to 89% (as measured in children ages less than 10 years) compared to villages randomized to enhanced coverage of 90% or greater?*

- The 80% to 89% coverage represents reasonable program targets. We consider greater than or equal to 90% an enhanced program coverage, requiring additional time and resources. All countries will be participating in the randomization arms for coverage, in order to determine the incremental effectiveness of extra effort to improve coverage in reducing infection and disease. We chose these targets for several reasons. First, trachoma coverage rates below 80% are not effective in reduction of infection in communities. Second, 80%-89% coverage is achievable by country programs using current distribution systems as reported in Niger and Tanzania (Note that The Gambia has not yet initiated its country program). Third, the extra effort to achieve greater than 90% coverage is manifest in terms of community manpower, targeted visits to uncovered households, and extra days of distribution to cover temporary absences from the community. We will use additional resources and mandates that are within country program ability. This specific aim will determine the effective coverage (80% or greater in children) of mass azithromycin treatment that will lead to reduction of infection and clinical trachoma for different trachoma endemicities.

*2) FREQUENCY OF MASS TREATMENT: What are the differences at three years post-baseline in rates of trachoma and ocular C. trachomatis infection in hyper-endemic villages in Niger randomized to mass treatment of children ages twelve and under biannually (every six months) compared to yearly mass treatment of everyone?*

- In Niger, we will be evaluating the effect of the two coverage targets, but also asking the question if treatment every six months of children is as effective as treatment yearly of everyone in the villages. We also address whether bi-annual treatment of children is more cost-effective than yearly treatment in rapid reduction of infection in hyper-endemic communities. We describe the cost component of this in another section.

*3) FREQUENCY OF MASS TREATMENT: What are the differences, at three years post baseline, in rates of trachoma and ocular C. trachomatis infection in mesoendemic villages in Tanzania and hypo-endemic villages in The Gambia randomized that use a laboratory based strategy to stop after one or two rounds of mass treatment, compared to villages randomized to receive yearly mass treatment for three years?*

- In this specific objective, we evaluate the potential increased cost-effectiveness, from the perspective of a trachoma control program. We will use a laboratory-based strategy to identify communities where there may not be further indication for antibiotic intervention. Communities can then “graduate” from mass treatment. The study will evaluate the effect on re-emergence of graduating communities which have evidence of infection rates less than 5%, or meet WHO definition for stopping (less than 5% Trachoma Follicular (TF)). Because the rates of disease and infection within Tanzania and The Gambia are not hyperendemic, the resources to treat on a mass scale more than yearly cannot be justified. Research in Tanzania has shown that re-emergence across households takes about one year, suggesting the yearly interval is reasonable.

## 1.3 Overall Design

We propose a community-randomized design within each of the three countries, which takes advantage of the level of endemicity to address questions of coverage and frequency. Within these study areas, communities will be randomized (minimum 16 in each arm) to two different coverage targets (80%-89% versus ≥90%) for three years of mass treatment. In Niger, we will further randomize communities to annual mass azithromycin distribution versus every 6-month distribution. Only children ages 12 and under will receive azithromycin treatment. In The Gambia and Tanzania, we will further randomize communities to yearly mass treatment versus annual mass treatment if warranted by the trachoma infection rates. We will evaluate the efficacy of guiding further mass treatment according to a laboratory test for Chlamydia or WHO guidelines. Where we estimate communities have infection rates less than 5% in sentinel children, or TF rates less than 5%, the community will be “graduated” from further mass treatment and followed for up to three years to look for evidence of re-emergent infection and disease. If rates of infection return to 20% at the 12 or 18-month survey, we will initiate mass treatment again.

Sentinel children randomly selected as cross sectional samples in these communities will be monitored at baseline, 6, 12, 18, 24, 30, and 36 months for infection and clinical disease. The three-year study is in accord with the WHO guidelines for three years of mass treatment followed by a re-survey to determine areas in need of further treatment.

Primary analyses will focus, within countries, on the comparison at three years of trachoma and infection rates in villages randomized to 90% or greater coverage versus less than 90%, and in villages randomized to yearly treatment of everyone versus 6 monthly treatment of children (Niger) or yearly versus in the “stopping rule” arm (Tanzania and The Gambia). Primary analyses will consider the 2 X 2 factorial design, the baseline prevalence, and baseline indicators of environmental hygiene as co-variates and effect modifiers. Secondary analyses will evaluate trachoma and infection in children, adjusting for clustering at the village level.

There are three ancillary studies nested within the trial. First, we will determine the cost-effectiveness of the different treatment coverage and administration frequency arms. The data on costs associated with a lab-based strategy to guide mass treatment cessation will assist in development of the cost of such a tool if it is finally developed. Moreover, we must weigh the potential additional costs associated with treatments every six months for children, or higher coverage in villages, against the benefit in terms of reduction of trachoma infection.

The second study will determine rates of re-emergence and the effect of multiple rounds of mass treatment within children. In communities selected at random within each arm, we will follow all children ages 9 years and under longitudinally after mass treatment for re-emergent trachoma infection and clinical disease. We will determine risk factors predicting re-emergence.

The third study will use the data collected from all three sites to develop mathematical models for use as public health planning tools to estimate the frequency and coverage of treatment necessary to determine a steep trajectory for elimination of infection and trachoma.

These three studies provide significant value to the trials in helping elucidate treatment “failures”, in providing cost-effectiveness data, as well as effectiveness data, on the treatment strategy alternatives, and in developing a model, that extends the three-year time frame of these trials to future expectations.

## 1.4 PRET Ziada

In January 2010, the PRET study group in Tanzania proposed to augment the current PRET study with the addition of PRET Ziada. PRET Ziada will utilize eight villages in Chamwino, Tanzania, with a starting trachoma prevalence between 10 and 20% to evaluate study question 3 only (frequency of mass treatment, stopping rule). The addition of PRET Ziada will allow the study group to evaluate the stopping rule in villages with lower prevalence levels (PRET villages have prevalence between 20 and 30%).

PRET Ziada will employ the same study design as described in the sections that follow, to include randomization, sample size, study procedures, data collection and analysis. All data will be presented to the DSMC on an annual basis.

# **Chapter 2: Background and Significance**

Chapter

2

_____________________________________________________________

## 2.1 Trachoma as a Public Health Problem

Trachoma, caused by ocular *Chlamydia trachomatis,* is the leading infectious cause of blindness worldwide. There are profound inequalities in the burden of trachoma, as it affects almost exclusively the poorest countries in the world, as measured by World Bank PovCal indices. Within affected countries, the geographic burden of trachoma is highest in relatively rural, poor regions. Research has shown that within these regions, trachoma disproportionately affects the most poor, and those who traditionally have no voice in leadership: the pool of trachoma in these impoverished communities is greatest in children less than five years of age, and women develop blindness from trachoma at a rate 2–3 times that of men.

In addition to the heavy personal and societal burden inflicted by loss of vision, there are significant economic impacts as well. The 3.8 million cases of blindness and 5.3 million cases of low vision due to trachoma are estimated to diminish productivity by $2.9 billion each year[[1]](#footnote-2).

In recognition of the public health impact of trachoma, the WHO established the Global Alliance to Eliminate blinding Trachoma by 2020 (GET 2020). A multi-faceted strategy to control all phases of trachoma has been endorsed by the WHO. It consists of **S**urgery (to repair lids distorted by trachoma (trichiasis) in imminent danger of vision loss), **A**ntibiotics (mass antibiotic treatment to reduce the community pool of infection with *Chlamydia trachomatis*), **F**ace washing (to reduce transmission from ocular and nasal secretions), and **E**nvironmental improvements (to interrupt transmission and prevent re-emergence). With even partial implementation of the SAFE strategy to 80% of those who need it, it is estimated that over 15 million DALYs per year globally would be saved[[2]](#footnote-3).

However, the trajectory for elimination of trachoma in these countries has been disappointingly slow. How long and with what frequency must we mass treat communities with antibiotics for active trachoma to achieve elimination? While WHO has recommended mass antibiotic treatment coverage targets of 80%, there are no solid data on how high the coverage should be and the frequency of administration. High antibiotic treatment coverage rates, and repeated treatments, may eliminate infection in some communities, but with almost a three to five year lag time between reduction in infection and significant reduction in the clinical signs of trachoma, communities remain targets of resources for mass treatment even when such resources may be better-deployed elsewhere[[3]](#footnote-4). It is estimated that antibiotic distribution costs for mass treatment in Tanzania, even with donated antibiotic, averages $5,000 per 100,000 population. Even with high coverage, some treated children with high loads of infection continue to have infection post treatment, arguing for approaches to targeted surveillance or more frequent coverage[[4]](#footnote-5). With renewed interest in trachoma as part of a battery of neglected tropical diseases, and the possibility where ecologically justified of integrated programs, there is an even more urgent need to clarify how best to use azithromycin.

The consortium brings together the leading trachoma research teams, at Johns Hopkins University (JHU), the London School of Hygiene and Tropical Medicine (LSHTM), and the Proctor Foundation at the University of California at San Francisco (UCSF). Consortium partners include Pfizer, who have pledged antibiotics for this project, advocacy partners including WHO and the Trachoma Control Programs in The Gambia, Tanzania, and Niger.

## 2.2 Anticipated Impact

Working with Ministries of Health and country programs, this trial of mass antibiotic intervention has the potential to alter the trajectory of blinding trachoma dramatically. This includes countries where trachoma is on the verge of elimination (The Gambia), where national programs have been in place and disease rates are declining, but elimination is slow (Tanzania) and in areas where disease and infection were formerly hyperendemic but have had a lapse in programmatic activities (Niger). No other research partnership has the breadth and ability to undertake such a comprehensive, and critical, proposal for trachoma control. We have proposed the dissemination of results in a format for country National Trachoma Control programs and Ministries of Health, the ultimate end users who need this evidence base to drive improvements in implementation of SAFE for trachoma elimination.

# **Chapter 3: Countries and Populations**

Chapter

3

We plan to carry out our research into more effective use of azithromycin in trachoma endemic sites in Niger, The Gambia and Tanzania. Each site was chosen because of the active involvement of partners in trachoma research and programs in the country, the ecology of trachoma in those areas (which is crucial for generalizability of findings to other countries), and the commitment to elimination of trachoma. The project will be carried out simultaneously in three countries, with overlapping and unique features in each. In order to determine the impact of our treatment arms on trachoma and infection, we will sample a sentinel group of children within randomly selected communities. We define communities as the smallest population unit for which health services are organized and trachoma control programs are implemented. These communities vary in size between countries. In Niger, we will use grappes, comprised of 250-600 people, as communities. In The Gambia, we will use Census Enumeration Areas, of population size 600-800 as communities. In Tanzania, we will use mtaas or clusters of mtaas (geographically distinct sub units of villages), which average around 1200 population. Each of the country settings are described more fully below.

## 3.1 Niger

Historically, Niger has been hyperendemic for trachoma.  Most individuals in the country received oral azithromycin in the mid 2000s, but the program has lapsed over the last 3 years.  There is evidence that clinical trachoma has returned in some areas since the last treatment.  The prevalence of clinically active trachoma in Matameye district has been estimated, and the Programme National de Lutte Contre la Cécité (PNLCC) and Carter Center have determined that retreatment is warranted. Previous data suggest at least three rounds of mass treatment will be necessary[[5]](#footnote-6).

## 3.2 Tanzania

In Tanzania, trachoma is prevalent largely throughout the country. The Dodoma region had the highest rates of trachoma in Tanzania, and the Tanzania National Trachoma Control Program started its activities in that region. Kongwa is a district in Dodoma, comprised of largely subsistence farmers. The Kongwa Trachoma project in collaboration with Johns Hopkins University was started in 1986 to document the burden of disease, and has since been the field site for studies of trachoma, including development of diagnostics, immunological studies, and clinical trials of face washing, fly control, and azithromycin use in communities. In this setting, program activities over the last eight years have reduced infection and disease from greater than 50% to 10%-30%, but at a slow pace. We expect that with more rigorous coverage and surveys to stop mass treatment when warranted, we can improve cost effectiveness of the programs. The Kongwa Trachoma project with Johns Hopkins University has been an integral part of the Tanzania National Trachoma Control Program, conducting surveys to document the effect of the program in regions all over Tanzania.

## 3.3 The Gambia

In The Gambia, there has been a decline in the national prevalence of active trachoma in recent years (from 20% to 7% in national surveys in 1986 and 1996: adjusted to 1-9 age group) but a survey in 2006 showed that substantial parts of Lower River Division (LRD) and North Bank Division(NBD) still have more than 10% prevalence of active trachoma in children under 10, and there is evidence that active trachoma persists at potentially blinding levels in foci elsewhere in the country. The program has identified 11 rural districts (rural districts vary from 5-20,000 population) and a total population of 150,000 who need mass treatment based on the WHO criteria. There has been a longstanding relationship between the National Eye Care Program and the LSHTM trachoma research group based at the Medical Research Council Laboratories which has led to a series of studies focused both on diagnostic and immunological studies in trachoma and operational research for trachoma control.

# **Chapter 4: Randomization Arms and Sample Size**

Chapter

4

________________________________________________________________

4.1 Overview

We propose to randomize communities in a 2 X 2 factorial design within each of the three countries. Communities will be randomized (minimum 16 in each arm) to two different coverage targets (80%-89% versus ≥90%) for three years of mass treatment. In Niger, communities will be further randomized to annual mass azithromycin distribution for everyone versus every 6 month distribution in children ages twelve and under. In The Gambia and Tanzania, communities will be randomized to yearly mass treatment versus yearly mass treatment if warranted by the infection /disease rates. We will evaluate the efficacy of guiding further mass treatment according to a laboratory test for Chlamydia or WHO guidelines. Where we estimate communities have infection rates less than 5% in sentinel children, or TF rates less than 5%, the community will be “graduated” from further mass treatment and followed for up to three years to look for evidence of re-emergent infection and disease. If rates of infection return at the 12 or 18 month survey to 20%, then mass treatment will again be initiated as clearly the stopping rule will have failed.

Sentinel children age five years and younger will be randomly selected from each community based on census of the community, as cross sectional samples of the rates of trachoma and infection. The census will be updated each year, and villages monitored at baseline, 6, 12, 18, 24, 30, and 36 months for infection and clinical disease. The three-year study is in accord with the WHO guidelines for three years of annual mass treatment followed by a re-survey to determine need for further treatment.

## 4.2 Randomization Arms

The basic structure of the trial is shown in Table 1. In this 2 X 2 factorial design, communities in each location will be randomized into four treatment arms, but these arms will differ by country. All communities will receive mass treatment with azithromycin at baseline. At present, recommended coverage of the community is 80% or better. We propose to evaluate the effect of two coverage targets, (estimated in children age 0 to 9 years old). The two arms would have 1:1 randomization at community level to one of two coverage targets: 80% to 89% coverage (reasonable program targets) and ≥90% (enhanced program coverage, requiring additional time and resources). All countries will be participating in the randomization arms for coverage, in order to determine the incremental effectiveness of extra effort in coverage in reducing infection and disease.

The second component of the antibiotic trial is frequency of administration of mass treatment. We propose two frequencies of mass antibiotic administration, yearly for everyone in the community (The Gambia, Niger, and Tanzania) and every six months for children ages twelve and under (Niger). The rates of disease and infection within Tanzania and The Gambia are not hyperendemic, and the resources to mass treat more than yearly cannot be justified. Research in Tanzania has shown that re-emergence across households takes about one year, suggesting this interval is reasonable. Therefore, for communities within Tanzania and The Gambia, we propose an alternative frequency regime: communities receiving yearly mass treatment will be further randomized to two arms: yearly mass treatment every year for three years (similar to the Nigerien arm), versus an arm that allows mass treatment cessation if the community achieves our clinical or infection goals. These goals are a prevalence rate in 0-5 year olds of less than 5% trachoma (defined as TF rates less than 5%) or 5% infection (defined as laboratory positive rate of less than 5%).

## 4.3 Communities and Sentinel Children

In order to determine the impact of our treatment arms on trachoma and infection, we will randomly sample a sentinel group of up to 100 children within randomly selected communities. *We define communities as the smallest population unit for which health services are organized and trachoma control programs are implemented*. These communities vary in size between countries. In Niger, we will use Grappes, comprised of 250- 600 people, as communities. In The Gambia, we will use Census Enumeration Areas, of population size 600-800 as communities. In Tanzania, we will use mtaas or clusters of mtaas, which average around 1200 population. As described below, we plan to have 8 communities in each cell of the Tanzanian arms, and 12 communities in each cell of the Nigerien and The Gambian arms. We need multiple communities to have a stable estimate of the impact of the treatment arm.

We also need a large enough sample **within** the communities for The Gambia and Tanzania, in order to be able to determine if mass treatment is to cease, based on our infection and disease rules for stopping. The universe of children being sampled differs for each of the country settings, as it relates to the size of the community. In Tanzania, of the potentially 16,000 children who form the universe of the sample, 3200 will be randomly selected, or one in five. In The Gambia, 48 Census Enumeration Areas (CEA) will be randomly selected of the 114 to serve as sentinel communities. With about 700 persons total and 175 children in the eligible age range, the total universe for this sample is 8400 children. 4800 children will be randomly selected at each survey, for a slightly higher than 1/2 chance of selection. In Niger, the study area is comprised of 6 Centre de Santé Intégrés (CSI) or integrated health centers composed of 235 grappes (roughly comparable to a village with an average size of 375 people). 48 of these grappes were randomly selected to serve as study communities (8 grappes from each of the 6 CSI), with up to 100 children and 40 randomly selected adults included in each round. Note, that there is no stopping rule in Niger, and thus a sample size smaller than 100 children is not an issue.

For this trial, we propose to monitor the changes in prevalence and infection over time according to treatment arm. For this, simple cross-sectional samples of up to 100 children ages 0 to 5 years within the community at specified times are sufficient. This sample will enable us to determine the effect of treatment after each round, and if treatment is to be stopped in the communities of The Gambia and Tanzania. This sample will not be followed longitudinally, and at each sampling time, a new random sample will be drawn. To support the sampling, a census update will be performed yearly.

### 4.3.1 Details on Randomization for Niger

Figure 1 illustrates the sampling plan for Niger. We will be working in the Matameye department of the Zinder Region. We will randomize at the grappe level.

For randomization, we stratified based on geography and endemicity. As indicated above, we began with six CSIs. Each of these six CSIs contributed 8 grappes to the study—with two grappes per CSI allocated to each of the 4 arms (for a total of twelve grappes in each of four arms, or 48 total). Moreover, we ranked the eight grappes from each CSI by clinical activity in 0-5 year old children. The top four of these were considered higher prevalence, and the lower four were considered lower prevalence. When randomizing, one higher prevalence grappe from each CSI was randomized to each treatment arm, and one lower prevalence grappe was randomized to each treatment arm. Finally, each study arm has one randomly chosen grappe intended for detailed longitudinal follow-up.

Each of the 48 grappes will have a complete census; the census will be used to randomly select children as well as provide the basis for data on treatment according to randomization into treatment arm. After the census, we will have a complete listing of all children five years or younger. One hundred of these children will be randomly selected, by assigning each child a random number and choosing the children 0-5 years associated with the lowest 100 numbers (or all children if a grappe has less than 100 in this age group). A second group of 20 children will be randomly selected as a substitution group if not all children from the first group of 100 are available. If a child on the list of 100 is not examined, the reason will be recorded (anticipated reasons include: moved to nearby grappe, moved to Maradi, Zinder, or Nigeria, absent for the day, absent more than 1 month, sick, or guardian refused). In addition, 40 adults (age 15+) will be randomly selected for monitoring at each collection.

### 4.3.2 Details on Randomization for Tanzania

Figure 2 illustrates the sampling plan for Tanzania .We will be working in the district of Kongwa Tanzania, which contains 67 villages. Of these villages, 10 are either large towns with low trachoma rates or villages known to have trachoma rates that are less than 10%. The remaining 57 villages are eligible for this trial. We will have some preliminary data on trachoma rates (data from other surveys past 2005) on all of them by end of February, and plan to identify the mtaas or clusters of mtaas within each village and stratify them into groups by rates of trachoma. The overall distribution of villages into these categories will dictate the distribution in each of the arms of the trial. We plan to use a SAS macro for constrained randomization, for this community randomized design. This approach reduces the likelihood of a bad randomization outcome by balancing on baseline trachoma as co-variates [[6]](#footnote-7). We plan to randomly select then 9 communities in each arm, one to serve as an alternate in case a community selected is either not eligible or refuses to participate.

Each of the 2 X 2 cells in Figure 2 are marked as “A, B, C, or D” and each will have eight communities randomly assigned until 32 communities are selected for the trial using the allocations that meet the balance criteria.

Each of the 32 communities will have a complete census carried out; the census will be used to randomly select children as well as provide the basis for data on mass treatment according to randomization into treatment arm. After the census, we will have a complete listing of all households with at least one child age five years or younger. One hundred of these households will be randomly selected, by assigning a random number to each household and using the first 100 numbers. Within each household, we will also assign random numbers to each child age five years and younger and select the child with the lowest random number. This avoids clustering at the household level. In Tanzania, the communities are large and have on average 240 households, and 85% contain a child in this age range, so we anticipate no problem in having ample households for the sample. In the event that there are fewer than 100 eligible households, we will then randomly pick the households in which two children age 5 years and younger are selected, and note to adjust for household clustering. We plan to randomly select 110 households in total, with the final ten households held in reserve in case we are unable to examine the first 100 children. We need 100 children at each examination, and we will make considerable effort to find the child randomly selected in the first 100 households, but if they are known to be gone from the community over the examination period, or the household cannot be located over two visits at two different times, then a child will be selected off the alternate list.

### 4.3.3 Details on Randomization for The Gambia

Figure 3 illustrates the sampling plan for The Gambia. We will randomize, at district level, 60,000 total population, about 15,000 in each arm, for antibiotic treatment coverage. These communities will be treated with mass antibiotic yearly as described in the randomization section above, or allocated to stopping rules. We will work in the contiguous rural Gambian districts of Lower Badibu (est. 2008 population 15,556) Central Badibu (est. 2008 population 14,964), Foni Bintang Karanai (est. 2008 population 18,866) and Foni Kansala (est. 2008 population 15,394), which have all been identified in the Gambia’s national trachoma control plan as requiring annual mass treatment for three years.

We will use a random number generator program to assign each district to a 2 X 2 cell in Figure 3 marked as “A, B, C, or D”. Within each district the effects of each strategy will be assessed in 12 of the 30 census enumeration areas, which will be randomly chosen. A census enumeration area contains variously several small hamlets, one medium sized village or represents a subdivision of a large village. In these 12 EAs per district a census will be carried out; and the census will be used to randomly select children as well as provide the basis for data on mass treatment according to randomization into treatment arm. After the census, we will have a complete listing of all households within each EA, and will be able to identify those containing at least one child age five years or younger. In 9 EAs per district we shall take a 25% sample of children age 5 and under, by selecting households from the list at random until the sample includes 100 children age 5 and under. To ensure that a number of 100 is reached we will randomly select a further 10 households to serve as reserves. In 3 randomly chosen EAs we will sample all children 0-9 as described in the ancillary study.

## 4.4 Sample Size

### 4.4.1 Villages

We estimated the sample size requirements for our two-by-two factorial design using simulation[[7]](#footnote-8),[[8]](#footnote-9). Briefly, we generated simulated data assuming the alternative hypothesis and determined the sample size needed for how often (selecting 80%, our desired power) our statistical test would yield a rejection of the null hypothesis. We adopt a non-inferiority design[[9]](#footnote-10) for each factor with non-inferiority limits of 8%. Assuming a standard deviation of 0.05 within each arm, a correlation of 0.5 between baseline and 36 month results (derived from previous studies), and no interaction between factors, we estimate that a total of 32 communities provides greater than 80% power for each main effect (95% one-sided non-inferiority). Thus, for Tanzania, out of a total of 32 villages, 16 villages will be assigned to annual treatment and 16 to a stoppage rule. We expect that between 40% and 80% of communities randomized to the stopping rule arm in The Gambia and Tanzania will stop mass treatment after one or two rounds, based on previous work. For Niger and the Gambia, the smaller community sizes imply a higher variance. Conservatively, we assumed the variance was one third higher. Thus, to obtain greater than 80% power, we require 12 villages in each arm (for a total of 48 villages, again assuming a one-sided 95% non-inferiority comparison with limit 0.08). Neither the assumption of plausible variation in the interaction effect nor the assumption of a beta distribution rather than a normal distribution alters these estimates to any substantial degree.

In Niger, the sample size provides sufficient power to distinguish between the expected prevalence of annual and biannual treatment arms (approximately 10% and 1% respectively based on mathematical projections), and for all countries, between the expected prevalence after 3 annual treatments with greater than 90% coverage versus 3 annual treatments with 80%-90% coverage (approximately 2% and 13% respectively based on mathematical projections). Note that we will be able to conduct a standard superiority test[[10]](#footnote-11) with a power of at least 80% to detect a difference of 0.08 for each main effect, as determined by simulation.) In practice, we have elected to enroll 12 grappes in each of the 4 arms.

We show the sample size for each hypothesis and assumption, recognizing the simple approach requested is not the analytic plan, and show that the simulation approach yields comparable estimates, and which includes consideration for our approach to analyses.

**1) What are differences at 3 years post baseline in rates of trachoma and ocular *Chlamydia trachomatis* in communities randomized to mass antibiotic coverage of low (80-89%) versus high (>90%) coverage.**

*We hypothesize that higher coverage (as measured in ages <10 years) will result in lower prevalence of infection at three years in all countries.*

In principle, this could be addressed by a two-sample T-test. We estimate that the inclusion of 12 communities per arm in both The Gambia and Niger will provide greater than 80% power to detect a 6% difference in the prevalence of infection in individuals 0 to 5 years of age at 36 months, assuming a standard deviation of 5.0% and a two-tailed alpha of 0.05.

We computed the sample sizes for two-sample T-tests by finding solving the following standard equation for *n*:

$\Psi_{2n-2}\left(t_{\alpha/2,2n-2},\frac{\left|\delta\right|\sqrt{n}}{\sigma\sqrt{2}}\right)=\beta$ (Chow et al, 2003[[11]](#footnote-12)),

where sigma divided by the square root of n is the standard deviation (alpha=0.05), delta is the effect size, 1-beta is the power, $\Psi_{m}\left(x,k\right)$ denotes the cumulative distribution function of the non-central T-distribution with m degrees of freedom and non-centrality parameter k, and $t_{a,m}$ is the ath quantile of the T-distribution with m degrees of freedom and non-centrality parameter k, and $t_{a,m}$ is the ath quantile of the T-distribution with m degrees of freedom.

We will randomize 12 communities into each cell of the factorial design described below. We used the standard deviation found from the distribution of infection rates in Niger of communities of the size that are proposed for Niger and The Gambia. When we examine the distribution of infection rates in Niger of larger communities, the standard deviation drops to 4%. Thus, we estimate that the inclusion of 8 of the larger Tanzanian communities per arm will provide approximately 80% power to detect a 6% difference in the prevalence of infection in individuals 0 to 5 years of age at 36 months, assuming a standard deviation of 4.0%. This computation was conducted using the same formula as above. We will randomize 8 communities from Tanzania into each cell of the factorial design described below. We have used our judgment to propose that a 6% difference in the prevalence of infection is the clinically relevant difference we wish to detect. After three years of mass treatment, we expect infection rates to be low, and the difference in terms of re-emergence between, for example, 2% and 7% is insignificant. Similarly, re-emergence rates may be higher, but we do not expect differences in the rates, if at three years infection rates are 10% to 15%. Note that the community size in Tanzania is now between 1,000-2,000 persons, at least 1.5 times the size of Niger (300) or The Gambia (600-800).

**2) What are the differences at 3 years post baseline in the rate of ocular *C.trachomatis* in communities randomized to mass antibiotic treatment of children every 6 months versus yearly treatment of everyone? (Niger only)**

*We hypothesize that treatment of children every six months will result in a lower prevalence of chlamydial infection at three years in Niger compared to yearly treatment of all residents.*

As with Hypothesis 1, this could be addressed by a two-sample T-test. We estimate that the inclusion of 12 communities per arm in Niger will provide greater than 80% power to detect a 6% difference in the prevalence of infection in individuals 0 to 5 years of age at 36 months, assuming a standard deviation of 5.0% and a two-tailed alpha of 0.05.  This computation was conducted using the same computation as for Hypothesis 1.

**3) What are differences at 3 years post baseline in rates of trachoma and ocular *C. trachomatis* in communities randomized to yearly mass antibiotic treatment versus a strategy to discontinue treatment (graduate communities), if infection/disease reaches less than 5%? (Tanzania and The Gambia only)**

*We hypothesize that the prevalence rates of infection, trachoma will be no different in villages treated for three years versus villages in the arm with the graduation rule at three years in Tanzania and the Gambia.*

Here we use a non-inferiority analysis. A total of 8.06 Tanzanian communities is needed to allow the null hypothesis that the graduation rule is at least 6% inferior to be rejected with a power of greater than 80% using a 2-sided alpha of 5%, and assuming a standard deviation in the 36-month prevalence of 4%.  This computation was conducted using the same formula as in hypothesis 1 (see Chow et al, sec. 4.4.1). Note that with a one-sided test with an alpha of 0.05, 80% power is achieved with 7 Tanzanian communities[[12]](#footnote-13).

A total of 11.9 Gambian communities is needed to allow the null hypothesis that the graduation rule is at least 6% inferior to be rejected with a power of greater than 80% using a 2-sided alpha of 5%, and assuming a standard deviation in the 36-month prevalence of 5%. As before, we used the same formula as in hypothesis 1. For a one-sided test, 80% power is achieved with 10 communities.

We originally computed the sample size for an 8% limit to be considered non-inferior; because for this hypothesis we felt the data would show very low rates of infection (in order to be graduated, the estimate for a community must be 0%) and 6%-7% infection rates at this level would not result in significant re-emergence. However, for consistency sake, we show that we do have 80% power (as opposed to 90% power to detect 8% differences) with the sample size as justified to detect differences as low as 6%[[13]](#footnote-14), [[14]](#footnote-15).

### 4.4.2 Children

We chose a sample size of 100 children per community specifically for the Tanzanian and Gambian arms, where we need sufficient numbers of children to determine with precision if disease has fallen to less than 5%. At the same time, we balanced concerns for costs and survey difficulties if this methodology is to be used by country programs to decide if mass treatment can be stopped. With 100 children in the sample, if zero children have infection, the upper 95% confidence limit of the estimated prevalence is less than 5% (3%). For example, if one child in the sample has infection, then mass treatment will not be stopped, even though the estimate of the mean is 1%, as we cannot be 95% certain that the true prevalence is not above 5%. We cannot power the study for infection below 1%, as that would require over 3 times the sample size (368 children), and such a size would be prohibitively expensive in terms of laboratory testing and follow-up time. However, with our sample size of 100 if even one child has infection, we know the estimate is 1% with a 95% upper confidence bound of 5.4%. We recognize this is a conservative approach, but we are strengthening the likelihood that the true value is below 5% according to the upper confidence limit.

**Chapter 5: Eligibility, Consent and Enrollment**

Chapter

5

## 5.1 Eligible Communities

This study aims to determine the impact of different approaches to mass treatment on prevalence of trachoma and infection in villages that are hyperendemic, mesoendemic, and hypo-endemic for trachoma. Within each country, there may be communities that may fall into each category, but in general the hyperendemic communities will be located in Niger, the mesoendemic communities in Tanzania, and the hypo-endemic villages in The Gambia, based on previous surveys. Eligible communities for each country are described below:

To be eligible for the trial in Niger, a community must meet the following criteria:

1. The community must be located in the target district around Matameye
2. The village chief consents to participation in the trial (this does not obviate the need for individual consent, but without overall leadership consent, the community as a whole cannot be part of the trial)
3. The prevalence of TF/TI in the community must be >10.5%.
4. Communities must be more than four kilometers away from the center of any semi-urban areas, which are thought to have a lower prevalence of trachoma
5. Communities must have an estimated population of between 250-600 people.

To be eligible for the trial in Tanzania, a community must meet the following criteria:

1. The community must be located in the Kongwa District
2. The community leadership gives consent that the mtaa is willing to participate in the trial (this does not obviate the need for individual consent, but without overall leadership consent, the community as a whole cannot be part of the trial)
3. The estimate of the village prevalence of trachoma is 20% or greater in preschool age children. For Tanzania, we want to concentrate on trachoma endemicity that is not at the level of virtual elimination, as we will have ample numbers of communities from The Gambia that fall into this range.
4. If the population estimate from the last Tanzania census for the community is greater than 5,000 persons, the village is not included. This will effectively exclude Kongwa town and two large communities on the main road. We excluded them because the need to do census updates and mass treatment in these communities will consume excessive time for our team. If the population estimate for an mtaa is less than 1,000 persons, and it geographically cannot be combined with another mtaa, it is excluded.

To be eligible for the trial in The Gambia, an EA must meet the following criteria:

1. The EA must be located in the target districts
2. Where several villages are contained within the EA, each village leadership gives consent that the village is willing to participate in the trial (this does not obviate the need for individual consent, but without overall leadership consent, the village as a whole cannot be part of the trial)
3. Where the EA consists of a single village or a segment of a large community, consent is obtained from the community leadership (note that no village is larger than 4,000 persons or 6 EAs)
4. The EA must have a prevalence of trachoma greater than 5%.

We will use the best available data on prevalence prior to the baseline surveys to determine eligibility. Once the communities are randomized, and the baseline survey completed, if a community is not eligible on the basis of trachoma rates, then another community is chosen for that arm from the alternate list.

## 5.2 Eligible Children

Within each community, sentinel children will be randomly selected to serve as indices of the village rate of trachoma and ocular *C. trachomatis* infection. For each country, the eligibility criteria are identical. In order to be eligible to be a sentinel child, the child must meet the following criteria:

1. The child is age 5 years or younger
2. The child must be a resident in an eligible, sample community (defined as either living in the community since birth, or moved in with parents or guardians).
3. The child must not have an ocular condition that would preclude grading trachoma or taking an ocular specimen.
4. The child must be willing to have a swab taken as part of being a sentinel child (this is critical for The Gambia and Tanzania, as each swab result counts towards meeting the stopping rule)
5. The child must have an identifiable guardian capable of providing consent to participate.

While ideally, we would only wish to select one child per household, in locations with smaller communities, we may have no choice but to select more than one child per household. Therefore, we have not restricted the number of children who may participate per household. We discuss the need to adjust for household clustering in the analyses.

## 5.3 Consent Procedures

Procedures for gaining consent to conduct this project vary in each country, depending on the requirements in Niger, The Gambia, and Tanzania.

### 5.3.1 Niger

#### 5.3.1.1 Background

In Niger, all procedures and protocols must be approved by the National Ethics Committee. The National Ethics Committee has requested oral consent for all participants, following a standard protocol within the grappes. This study involves a random selection of children and adults, who will be screened for clinical trachoma and have a swab taken of the upper eye lid to determine presence of infection. In addition, a random sample of children will receive nasopharyngeal swabbing to test for pneumococcal resistance. All persons in participating grappes will have the opportunity to be treated according to their community’s treatment assignment. Grappes not included in one of the study arms will still be offered treatment through the national treatment program.

#### 5.3.1.2 Consent procedures

####

Several levels of recruitment are involved when working in Niger. At grappe level, our research team discusses the project with the village chief. Once leadership consents to participation, we will undertake a complete census of study areas by going house to house in each grappe (see Chapter 6). At this second level, we ask permission of the head of the household or an adult dwelling in the household to collect data on household characteristics and the age and gender of each resident of the household. At this time, we explain the following: the census will be used to monitor the uptake of mass treatment in the community and to draw a random sample of children ages 0-5. These children may be eligible to be in the surveys that will be done at baseline, and every six months after mass treatment. At this point we describe what the survey entails (trachoma assessment of the child, and a swab taken of the upper eye lid to detect Chlamydia, the bug that causes trachoma). We ask them to consider that, if the household contains a child of eligible age who is selected, we will return to seek permission to examine the child.

Our local team members will provide information and seek consent, as they are fluent in Hausa, the local language in the Matameye district. The Nigerien study coordinator will train the team in research ethics and informed consent. The team will explain that the purpose of the project is to determine severity of trachoma and infection before and after treatment by examining a sample of the children in the grappe. They will demonstrate the use of the swab and explain that participation is completely voluntary and will not affect access to health care or access to the mass treatment to come after the survey. Additionally, they will explain that different grappes will receive different treatments in an effort to determine the best treatment, and that treatment for trachoma will be provided to everyone in the grappe once examinations are completed. If a child is selected for a nasopharyngeal swab, another consent will be obtained, using similar procedures explained above. If the participant or the participant’s guardian agrees to participate, they will mark a fingerprint on the consent form. All consent forms will be kept by PNLCC in Niamey.

### 5.3.2 Tanzania

#### 5.3.2.1 Background

In Tanzania, all procedures and protocols go through the National Institute for Medical Research for Institutional Review Board approval within the country. In general, when we have submitted research on trachoma to them, we ask for permission to obtain oral consent for all participants, following a standard protocol within the villages. This study involves children, who will be screened for clinical trachoma and have a swab taken of the upper eye lid to determine presence of infection. All persons in the study villages will have the opportunity to be treated with oral azithromycin at baseline, as part of the yearly mass treatment effort in Kongwa district. Half the villages will also be offered mass treatment with azithromycin yearly over the next three years, and half the villages will be treated only if the sentinel children have no evidence of either infection or TF.

#### 5.3.2.2 Consent procedures

There are several levels of recruitment when working in Tanzania. The first is the level of the community, where our team goes and talks about the project to the community leadership. This information is then imparted throughout the village by a series of village meetings held by the leadership where the research project is discussed and participation is discussed. When our team returns, we must address specific questions the village may have before allowing us entry. While it is rare to have a village refuse to participate, we have had two villages refuse, in 18 years of research.

Once the community leadership consents, we then go house to house to undertake a complete census (see Chapter 6). At this second level, we ask permission of the head of the household or an adult dwelling in the household to collect data on household characteristics, and the age, and gender of each resident of the household. At this time, we explain the following:

- The census will be used to monitor the uptake of mass treatment in the community and to draw a random sample of children ages 0-5 who will represent the children in this community.
- If this household includes children ages 0 to 5 years, these children may be eligible to be in the surveys that will be done at baseline, and every six months after mass treatment.
- Participation in the survey entails trachoma assessment of the child, and a swab taken of the upper eye lid to detect Chlamydia, the bug that causes trachoma.
- We ask them to consider that if the household contains a child of eligible age who is selected, we will return to seek permission to examine the child.

We plan to have our local team members provide the information and seek consent, as they are fluent in Swahili and the local language for Kongwa, Chigogo. Mr. Mkocha will train the team using the Johns Hopkins IRB course in research ethics and informed consent. The team will explain that the purpose of the project is to determine rates of trachoma and infection before and after treatment in this village, by examining a sample of the children in the village. They will demonstrate the use of the swab, and explain that participation of the child is completely voluntary and will not affect access to health care, or access to the mass treatment to come after the survey. They will explain that treatment for trachoma will be provided after the research survey is completed, to everyone in the village, but the family will be told the results of the examination. He will explain the project to the parents in the presence of the child, and show him/her what the examination entails. However, the parent is the decision maker as children age five (or even nine) and under have not yet gone through ceremonies for passage to adult hood and therefore will not countermand adult authority-asking them is a courtesy but rarely will an answer be obtained that is different.

Once the sample is drawn, our team will approach the child’s mother or father directly to seek verbal consent for participation in the survey. We have always sought verbal consent for our studies in the rural villages of Tanzania. Most people are functionally illiterate and are asked to sign their names for taxes, collection of money, or more sinister schemes where their land has been taken away. We consider it unethical to have them sign a piece of paper they cannot read, and have always requested approval to obtain witnessed verbal consent.

### 5.3.3 The Gambia

Fully informed consent will be sought using procedures approved by the Gambia Government/MRC joint Ethics Committee. Consent is obtained at village level, similar as to that described for Tanzania, from the village leadership at a village meeting using the attached information sheet (in Appendix 1. At individual level, it is considered that children over six should receive an explanation of the study according to the attached information sheet (Appendix 1), and give their individual consent (see Appendix 1 and that for younger children individual informed consent may be obtained from a guardian is used. As many rural Gambian people are not literate, documentation of witnessed consent using independent literate witnesses is provided for. The attached consent forms are subject to approval by the Gambia Government MRC joint Ethics Committee.

## 5.4 Risks and Efforts to Minimize Risk

There are minimal risks to the participants. Flipping the eyelid is only slightly uncomfortable and most children are not bothered by the procedure (in fact, many children can do it themselves). The swab is uncomfortable, and can be a painful procedure if the eye is very inflamed. However, the procedure does not harm the child, other than upset them for a few moments if it is uncomfortable. By informing the parents about the clinical status of the child, and motivating them to seek treatment, the assessment is actually helpful. There is no alternative to the clinical examination for determining trachoma status.

There is the theoretical risk of transmitting trachoma from one child to another during the examination. Our procedures should reduce that to virtually zero, and even if there is transmission, the mass treatment that follows our survey should eliminate the infection.

There is the theoretical risk of being stigmatized for having trachoma. However, we have never found that to be an issue in working in these villages, as people are aware that blindness is an issue and that trachoma is common. These villages have been part of a control program for a number of years, so they are aware of the condition in their environment. As many of these children have trachoma, there is no stigma to being told one has the eye disease. The parents will be in attendance and will be told of the findings at the time of the examination.

Procedures for protection against risk include the following: we are careful to have a single individual flip the lid while the senior lab technician carries out the examination, takes the swab, and the photograph of the upper eye lid. In this way, any psychological trauma to the child, or discomfort from having the eyelid flipped is minimized as the lab technician can work quickly. In addition, by having the person hold the eyelid, the swab procedure can be done safely without worry of accidental corneal scratch. We have never had an accident in all the years we have taken swabs for trachoma using these procedures. In the unlikely event of a corneal scratch, the team has an eye patch and topical tetracycline and can treat the scratch immediately. We will minimize any inadvertent transmission from one child to another by having all persons who touch the eyelid change gloves between each child. In the unlikely event that infection is transmitted, the mass treatment offered after our survey should eliminate any infection. The results of the examination are told to the parents immediately after the examination, and not revealed to other members of the village.

The forms are stored in a folder, which after data entry are kept in a locked file room. The data are transmitted to the main data centers without names, just identification numbers. Such procedures ensure confidentiality.

For villages that meet the stopping rule, mass treatment will be stopped after the survey in which criteria was met. Theoretically, then a community may receive one round of mass treatment, and not again until after the third survey. If infection is subsequently found in any of the sentinel children, the child may not receive azithromycin until after the third survey. We have previously received ethical approval to conduct longitudinal surveys in these communities following mass treatment, without re-treatment[[15]](#footnote-16),[[16]](#footnote-17),[[17]](#footnote-18). Because the infection is largely self limited and does resolve, by the time the PCR was completed and results known, the child would likely have no infection when we returned. If these communities have had no infection post treatment, which made then eligible for the stopping rule, the likelihood of experiencing repeated bouts of infection is remote. The risk of scarring takes at least five years with repeated bouts of infection, which under the conditions of the stopping rule, will be rare. In addition, the parents may seek tetracycline eye ointment which is kept at the health post or health centers as part of the essential drug kit.

# **Chapter 6: Procedures: Census of communities**

Chapter

6

____________________________________________________________________

## 6.1 Overview

In this Chapter, we discuss the procedures for conducting census of communities. The census is a critical piece of data collection, as it is the document by which we measure coverage of mass treatment in the community, and it is the sampling frame for drawing a random sample of children in each community. Once the communities have been selected, and agree to participate as described in Chapter 5 for each country, the survey teams will then conduct a full census. The methods for conducting the census will be the same for all countries.

In the context of this trial, as described earlier, the community in Tanzania is an mtaa or cluster of mtaas (population between 1,000 and 2,000 persons); in the Gambia, it is Enumeration areas (population between 600- 800 persons); and in Niger, the community is a grappe (population between 250 – 600 persons).

## 6.2 Baseline Census

The census team will meet with the community leaders, and collect a list of the head of household names. In Tanzania, this means a meeting with the balozi and mtaa leaders (neighborhood leaders); in The Gambia with a meeting of village elders and compound heads. In Niger, the meeting is with the chief of the grappe.

Each of the community leaders will assign a knowledgeable community resident to escort the census team member to the households under his/her jurisdiction. The household will be assigned a unique identification number which will be affixed to the door. These numbers will correspond to a community number followed by a unique three digit ID for that household (See Census Form, section 10.2). At the end of the census, the census team leader will go through the community to be sure that there is no door that is missing a number. If a household prefers not to have a permanent number affixed to the door, they will be asked to at least have a plastic sheet with the number taped to the door for the duration of the census so that they are not visited and enumerated more than once.

For each household, the census team member will ask to speak with either the male or female head of household to obtain a list of the names of all persons resident in the household, ages and gender. In Niger, the name of each child’s mother will also be recorded, as there is more than one wife/mother in each household. The definition of resident in the household means a person who has slept in this household for at least three month in the past or who intends to reside with the family for the next six months. For ages, the age of the children needs to be as precise as possible, as they comprise our sample. Vaccination or MC clinic cards will be used to obtain age, where available. Otherwise, event calendars specific to each location will be used to tie ages to such events as elections, weather events, significant deaths, etc.

Other demographic information to be collected in all sites will include education completed by the head of the household, distance to the closest source of water, whether there has been a health education program for face washing, observations on clean face status of children, presence of latrines, and cleanliness of the area around the doorway of the household. These measures enable us to further characterize households in communities where sustainable reduction in disease and infection has occurred, and enable us to compare across countries for any environmental effect on reduction in infection. Precise definitions are described in section 10.2.

The census data are entered into the data base using customized data entry forms in Access that are standard across all clinical sites. This component is described under data management section in Chapters 10 and 12.

## 6.3 Interim Census

The census will be updated at each year for the ensuing three years. The procedures are exactly the same for new households that come into the village since the previous census. The same census information will be collected on the household and each person in the household. For new additions to the households, or loss of persons in a household, addendums to the census list will be made indicating the age, gender, and relationship to head of household for the additions, or reasons for deletion of a person (death or permanently moved to another community). If the person has simply moved within the village, they will be deleted from their previous household and added to the new household using the update census form (see Chapter 10 section 10.2.).

These updated census lists will be used to draw a new random sample of children at each yearly survey, and provide the denominator for the mass treatment interventions.

Census data will also be updated during the time of the surveys. If children have died or moved, or households have moved away, then census update forms will be generated at the time of the survey to update the census list. The data from the surveys will only be able to update the status of children who were randomly selected for survey, but we will capture that information.

# **Chapter 7: Procedures: Study Visits**

Chapter

7

_______________________________________________________________

7.1 Overview

The purpose of the study visits is to collect the basic data on the sentinel children who will represent the community burden of trachoma and ocular *C. trachomatis* infection over time. Once the sentinel children are selected in each community, the study team will examine them for trachoma and take swabs for determination of laboratory evidence of infection. Swabs are sent to the laboratories for processing. New, cross-sectional samples will be drawn at each study visit, using the latest census survey as the sampling frame, and following the random selection procedures discussed in Chapter 4, section 4.3. The procedures for the study visits will be identical across all country sites, and will not vary over time with any of the study visits. The sample of 100 children can be examined in a single day, so we have set aside one day for the examinations and a second day for catch up. For logistical reasons, the teams cannot make more than two visits to a single community, so children who are not available for the examination within two days will be replaced from the alternate list as described in section 4.3.

The time line for study visits is shown in section 7.5. Basically, after the baseline visit, the villages receive mass treatment according to the randomization schedule with follow-up visits and further mass treatments scheduled as per treatment arm thereafter for three years. In the next sections we describe the content and procedures for the baseline and follow-up visits.

The study team for these visits consists, at a minimum, of an intake clerk, a trachoma grader/photographer/lab technician, and a driver. There may be separate persons who perform the functions of trachoma grader, photographer, and lab technician, and person who may perform tubing. A person whose job it is to solely flip lids may also be part of the team, and can help the others be more efficient. Because these functions may be combined in different ways in each country, we describe the job, rather than the person.

## 7.2 Baseline Visit

Once the child is selected, the family is notified of the selection. In Tanzania, an appointment card (“chiti”) that contains the child’s name, head of household and neighborhood leader’s name, and household ID is sent by the health worker to the family, with the date of the exam and location. The family is asked to bring the child to a central location in the neighborhood. In The Gambia, the household head and the child’s parents/guardians are notified prior to the visit. In Niger, the registration worker goes door to door to notify families of the exam visit.

The child arrives for the examination and is checked in by the intake clerk. The intake clerk has a Master List of eligible children for the community (see section 10.2.3) and a stack of pre-printed ocular examination forms with the child’s study identification information on the form. The intake clerk checks off the child as arriving on the Master List. The intake clerk completes the informed consent procedures, as described in section 5.3. If the guardian agrees to have the child be part of the survey, then the intake clerk signs the witness for verbal consent form (see Appendix 2). The intake clerk hands the guardian the correct ocular form with the attached labels, and instructs the guardian and child to wait for the examination.

The intake clerk keeps a running count of the children who are examined, and notes those who have failed to show up for an appointment. S/he assigns the community health worker to go to the households of children who have not appeared and determine the reason. We will make every effort to include such children, including offering transportation to and from the exam site, or a home visit if necessary. If the child has moved permanently or died, then the intake clerk will select a household and child from the alternate list, and complete a census update form (see section 10.2.2) If the child is not available during the examination times in the community due to travel or illness, the reason is noted on the Master list, and again an alternate child is selected.

At the end of the day, the intake clerk must account for all children on the Master list of eligible children. Either they have presented for examination, or a reason is provided for non-attendance, and an alternative appointment scheduled or an alternate selected. If the list is incomplete, the survey team must wait until the households are visited and the child is examined, another appointment made, or an alternate is examined.

The following describes the details of the procedures for the examination. An ocular form is filled out (see section 10.3 for ocular form details) as a record of the examination.

### 7.2.1 Trachoma Assessment

Equipment needed: ocular form, black pen, torch light, 2.5X loupes.

Before examining the child, the grader should examine the ocular form, be certain it contains labels for the swab and check if a photograph is necessary. The examiner should call out the child’s name and ascertain that the child (by name age and gender) is correct. If incorrect, the child and guardian should be sent back to the intake clerk for resolution-either the form sent in was incorrect, or the wrong child was brought for the examination.

To examine the patient for trachoma, it may be necessary to have guardian hold the child securely in the lap, with the child’s legs around the guardian’s waist and the guardian holding the child’s hands. The child’s head is then in the lap of the examiner/lid flipper. The child is told to look “down” at the guardian, as it is easier to flip lids. Once the person who flips the lids has on a new pair of gloves, s/he should NOT touch the child anywhere else but on the eyelid to ensure no contamination. Trachoma is assessed by flipping the upper eye lid and examining the tarsal plate for evidence of trachoma. Be certain that the pressure on the upper eye lid as it is everted is not too strong or it will blanche and make grading very difficult. If necessary, let the lid go between grading and photography. The left eye is flipped first, and graded, followed by the right eye; the right eye is then photographed (selected) and swabbed. The person who flips the lids will be wearing a pair of gloves which are exchanged between each patient. The trachoma grader or lab technician may or may not be the “lid flipper”.

We plan to use the WHO simplified grading scheme for trachoma, which assesses the presence or absence of follicular trachoma (TF), intense trachoma (TI), conjunctival scarring (TS), trichiasis (TT), and corneal opacity (CO).[[18]](#footnote-19) For this study, the relevant signs are TF and TI, signs of active trachoma. TF is defined as five or more follicles in the region of interest of size 0.5 mm. If there are fewer follicles, then TF is graded as not present. TI is defined as inflammation severe enough to obscure 50% of the deep tarsal vessels. Occasionally, but rarely in children, there is scarring sufficient to obscure the vessels-however, this is not TI as the obscuration is not the result of inflammation. Where scarring is this bad, the eyelid may be labeled as “cannot grade” for signs of active trachoma. Senior graders, who have all been standardized to Dr Bailey at LSHTM, will perform all field gradings. The data on the presence or absence of trachoma signs is entered onto the form.

If more than one grader is used per country, then graders will be assigned randomly to intervention and control communities, to ensure no subtle grader bias due to doing only one kind of village exists in the treatment arms. Further quality control procedures are discussed in section Chapters 13 and 17.

### 7.2.2 Laboratory Procedures

#### 7.2.2.1 Specimen Collection

Equipment needed: sterile swabs, swab vials, labels, ocular swab shipping list.

Each child who is serving as a sentinel child must have an ocular swab taken for determination of infection. If the child refuses the swab, or a swab cannot be taken for another reason, then the child is ineligible. The intake clerk should be notified that the child is ineligible, a note made in ink on the ocular examination form and in the Master List of Eligible Children, and an alternate household and child selected as replacement.

Each ocular form has a set of printed labels with the child’s study identification number. One label is placed on the ocular form in the section asking if a specimen was taken, one on the study vial after the swab is inserted, and one on the shipping log for the box in which the vial is inserted. In this way, each vial box has an accounting of specimens for subsequent shipping. There are extra labels as well, if one is lost or torn.

We will be taking a field control swab for each laboratory technician sufficient to have 50 control swabs per survey. These will be taken using the “blue air” swabs that will be pre-marked in each box of swabs.

#### 7.2.2.2 Protocol for swabbing of the conjunctiva

1. Upon taking a swab from the box, the lab technician should announce what type of swab it is:

- An unmarked “plain swab”, indicating no control collection necessary for this patient.
- A "blue air swab" (negative field control), indicating examiner must collect a second swab for this patient. Swab sachet will be marked with blue color.

1. The swab sachet should be opened in a sterile manner, revealing only the tip of the swab shaft, with the swab head itself remaining sterile deep within the sachet.
2. If the child needs an ocular photograph, make sure this is taken prior to the swab. When ready to swab, the lab technician should slowly pull out the swab (if removed too quickly the Dacron swab tip can unravel). The gloved hand should be held no closer than 1 inch from the Dacron polyester swab tip during the entire swabbing procedure, in order to avoid contamination of the swab tip.
3. As the lid flipper (who may be the technician) holds the right eyelid in the everted position, the technician swabs the upper tarsal conjunctiva of the child with a gloved hand, using a steady and firm swab. Ideally the swab should be placed flat, with its entire length parallel to the conjunctiva to give the greatest surface area of contact, and if necessary, this may require repositioning of the child’s head by the examiner. The swab should be drawn firmly in one direction over the conjunctiva with enough pressure to cause blanching of the conjunctival vessels. The swab should then be rotated 120 degrees along its axis and the newly revealed fresh region of Dacron on the swab tip should now be drawn firmly across the conjunctiva. The swab should be rotated another 120 degrees along its axis and the conjunctiva swabbed for a third time. This will ensure sufficient collection of conjunctival epithelial specimen for PCR analysis in the lab.

**Note:** An aggressive twisting motion toward the cornea can push the conjunctiva upwards (towards eyebrow) which can lead to the swab falling onto cornea. Thus a pure lateral motion or slight twisting toward the eyebrow is preferable, in order to ensure safety of the cornea at all times. Quick movements should be avoided (less control, increased contamination, risk of touching the cornea). Instead, a slow, steady motion should be carried out. Care should be taken to avoid swabbing the eyelashes. Traction on the lower lid by the non-swabbing hand (or by an assistant) can keep the lower lid lashes from inadvertently touching the swab.

#### 7.2.2.3 PCR Control Swabs

A negative field control ("blue air swab") will be taken on a randomly chosen 50 children per lab technician/grader to assess the frequency of contamination. For each negative field control, the examiner will pass a sterile Dacron swab within 1 inch of the child’s conjunctiva. Control swabs are taken after the original swab but before changing gloves for the next patient. A label from the control set, made to look like a regular ID but indicating a control swab and generated from the CCU, will be used. One label is affixed to the ocular form in the indicated area and one is affixed to the vial, and a third to the ocular specimen shipping list.

#### 7.2.2.4 Protocol for tubing and handling of samples

The tubing and handling protocol must be carefully followed in order to prevent contamination and ensure the safe transport of the samples back to the microbiology laboratory. The person in charge of labeling, tubing, arranging, and handling the samples needs to perform this task in the most orderly and attentive manner.

- 1. Both hands of the tuber should be gloved at all times. The tuber's gloves only need to be changed when any potential contamination of the gloves occurs. The tuber opens the capped, hinged lid of a microcentrifuge tube, which has been labeled with the participant’s random identification number. If it is a control or “blue air” swab, then the label comes from the control set of labels.
  2. The swab is inserted by the lab technician (using the still-gloved hand that swabbed the participant’s conjunctiva) into the microcentrifuge tube held by the tuber. The swab shaft should only be inserted until the Dacron swab head is fully in the tube. The tuber should lower the cap onto the swab shaft held by the examiner, and the examiner should quickly break the swab shaft using a swift downward snapping wrist movement.
  3. The tuber should screw the cap of the microcentrifuge tube tightly and place it in the sample collection box, located in the cooler bag filled with frozen ice packs. The flap of the cooler bag should be closed between each patient. The cooler bag should be in as cool a place as possible in the field, in a shaded area out of the sun.
  4. Upon returning from the field each day, the samples will be immediately taken to the field office and stored in a freezer, reserved solely for storage of specimens and ice packs. All samples will be in sample boxes, labeled with the grappe (and, if necessary, district) names for easy future identification.

### 7.2.3 Shipping and Storage of Samples

In accordance with the Roche COBAS AMPLICOR™ CT/NG protocol, swab samples taken in the field will be transported on ice in a closed, insulated container until arrival at the laboratories in Baltimore, San Francisco, and at the MRC in The Gambia, where they will be stored at -80°C for later analysis. Samples will be imported in the USA as per the CDC Permit to Import/Transfer Etiological Agents or Vectors of Human Disease.

1. Materials for shipping to the US

- Insulated shipper box (ThermoSafe VIP insulated shipper, 615DCS or 499 DCS)
- Gel Packs (ThermoSafe U-tek 24 oz. -10°F)
- Shipper label
- Consignee label
- Responsible person label
- UN3373 label
- Biological substance, Category B label
- Documentation to include in shipper box
  - Pro-forma invoice
  - Letter from Shipper
  - CDC permit
  - Letter of approval from Ethics committee in country
  - airway bill from ;world Courier, DHL, or other air transport service

2. Packing the samples

For dry samples without media:

- Boxes can be wrapped with plastic wrap and clear plastic tape only. There is no need to use pressure-resistant bags.
- Boxes may also be wrapped with a cotton absorbent pad (chuck) for improved insulation.
- As soon as samples are prepared for shipping, they should be returned to the -20°C freezer to prevent samples from thawing.
- Place all stickers on outside of the shipper box (see detailed shipper instructions).
- Leave at least one shipping box, preferably a partially-filled one, unpacked for inspection by the air shipper employee and or customs.

When ready to ship, the prepared sample boxes should be placed into the partially filled shipper box.

Next, all frozen icepacks and samples should be placed in the shipper box. (6 gel packs per a small shipper, 12 gel packs per a large shipper)

- For a small shipper box: Use 6 frozen gel packs-- one below sample boxes, 4 on each side, and one on top of sample boxes
- For a large shipper box: Use 12 frozen gel packs-- 6 gel packs flat at the bottom and 6 on the top

Immediately after the shipment is accepted by the shipping company, the shipper box should be closed and taped shut using clear plastic tape. Tape should be placed around all corners of the outside box.

### 7.2.4 Laboratory Processing Procedures

A dry swab is taken in the field, a study identification label is affixed, and the vial is kept frozen until it reaches each country team’s designated laboratory as described above. The specimens are processed according to strict protocol, outlined in the manufacturer’s kit directions, followed by the Johns Hopkins International Chlamydia Laboratory, the Molecular Biology Laboratory at the University of California at San Francisco, and the laboratory at the medical Research Council in The Gambia. We will use the Roche Amplicor *C. trachomatis* qualitative PCR assay from Roche Molecular Systems. Procedures are summarized as follows: Each swab is eluted by vortexing in Amplicor CT/NG lysis buffer in polypropylene tubes, and then Amplicor specimen diluent is added. Using a known positive sample in the laboratory, we will create positive and negative C. trachomatis (CT) processing controls; two CT+ and two CT- processing controls are run with each batch of specimens. The Working master mix is created and the specimens prepared prior to amplification. The specimens and controls are placed in the thermal cycler for amplification. Once completed, the specimens are denatured, and sent for detection using probe-coated microwell plates. Detection is accomplished by measuring the optical density at A_450_. The assay result for the negative controls should be less than 0.2 A_450_, and the assay result for the positive controls should be 0.8 or greater for ocular specimens for a valid run. We will test separately for amplification of both the target plasmid DNA and the master-mix internal control samples to determine if PCR was inhibited. Samples whose values in valid runs are ≥0.8 A_450_ are counted as positive, and samples less than 0.2 A_450_ are negative. Samples for which the result are equivocal (≥0,2, <0.8) will be tested again; if equivocal twice, they are left as equivocal and called not positive in the analyses as no run equaled 0.8 or greater.

### 7.2.5 Photograph Grading

An ocular photograph will be taken of the right eye of every nth child sufficient to ensure at least 50 photographs per trachoma grader per survey round. In Tanzania, at 100 children per each of 32 communities, or 3200 children per survey round, this is about every 32nd child seen by each of the two graders. In The Gambia and Tanzania, more photographs will need to be taken to ensure that at least 25-50% has trachoma. To accomplish this, on every Master List for a community a star will be printed on the ocular forms of 3-4 children who need photographs. They will also have two extra, colored labels printed indicating they need a photograph. The labels are placed in the photo log book (see Chapter 10 section 10.4). In Niger, there will be a greater number of examiners. To ensure quality, we will collect photos on every participant presenting for examination.

Clinical photography will be performed before conjunctival swabbing. The protocol for photography is included below.

### 7.2.6 Photography Protocol

Equipment needed: camera, labels, photo log book, and CD burner

Miscellaneous equipment: Extra battery, card reader, lens filter, extra media card

#### 7.2.6.1 Camera Setup

A handheld Nikon D-series camera and lens will be used for all photographs. If possible, use natural light rather than a flash, as the flash introduces artifacts into the image.

1. Model
   1. Nikon D-series camera: D-40, 50, 70, 80, 90, 100 or 200
   2. There are minor differences between the different models, mostly related to setup
2. Lens
   1. Nikon Macro Autofocus 105 mm f/2.8
   2. Manual setting- turn off autofocus
   3. Disengage “limit” engage “full”
   4. Extend lens to 1:1; note with older versions of this lens it is 1:1 at full extension; newer versions can go to slightly higher magnification than 1:1 so caution must be exerted
3. Image size/resolution
   1. Jpg normal- set thru “setup” menu
   2. Large- set thru “setup” menu (“small and medium” are probably adequate)
4. White balance- automatic or flash; set thru “setup” menu
5. Shutter- Aperture Priority
   1. f57- set thru menu; note if lens is not extended to 1:1, f57 will not be allowed
6. ISO 400

#### 7.2.6.2 Photograph Procedure

1. Place patient into position that will allow maximum stability; either standing, sitting or “head-clamp” position. Employing village volunteer to help is very useful.
2. Take photo of patient ID
3. Image is brought into focus by changing the working distance, not by turning the lens. This is because the lens is fixed in its manual setting. The working distance is approximately 20 cm from the eye with our current settings
4. Take minimum of 2 photos. If there is any doubt of the quality of the photo while the patient is in position it is better to continue to take more photographs before the patient is allowed to leave. It is easy to delete photos if they are not needed.
5. Check photos before allowing child to leave. If they are not acceptable, repeat procedure. Only stop if the patient or guardian requests that we stop, or if is deemed impossible, even with further attempts. Note we have obtained >95% acceptable photos in previous studies.
6. If the child cannot be photographed for some reason, it does not affect the eligibility of the child. A notation is made on the ocular form of why the photograph cannot be taken, but no replacement is sought. Again, this is expected to be a rare event.

Images are stored in digital file folders according to study ID number. They are burned onto a CD every week and sent to LSHTM where they are graded for quality assurance purposes by Dr Robin Bailey, who is the standard grader for the PRET study. This is discussed further in section 13.7.

#### 7.2.6.3 Photo TROUBLESHOOTING GUIDE

Note all camera settings are permanently embedded in every photo that is taken and can be viewed with the camera or with any standard commercial photo-viewing software (e.g., Adobe Photoshop; Photomechanic, Nikon View, Canon, etc.)

1. Photos too DARK, too LIGHT or OUT OF FOCUS
   1. Check camera settings: ISO 400, Aperture preferred, +/- on zero, Manual setting on lens, flash elevated; battery fully charged
   2. If all photos are affected in the same way than it is most likely the settings on the camera or lens
2. Change camera settings from default recommendations
   1. Decrease F stop to lighten, increase to darken
   2. Could be taken down to 32 or lower but depth of field will be lessened
   3. +/- can be increased or decreased to change exposure (+ to lighten; - to darken)
   4. Check that lens is on Manual NOT Autofocus
   5. ISO- increase to lighten photo, decrease to darken
   6. Very high ISO will produce graininess
   7. Meter- try “spot” instead of “pattern
3. Have another person observe photography in real time
   1. Make sure flash is not depressed by photographer or someone else while photo is taken. (This commonly happens if photographer has loupes). Is the lens well supported?
4. Make sure battery is fully charged
   1. If first photos in series are acceptable and then they gradually become less exposed (darker) it might be because the battery is gradually losing power during the session. Note that the flash reaction time increases as the battery power decreases.
5. Out of focus
   1. F stop may be too low; movement artifact; flash doesn’t work; lens is dirty
6. Image not centered
   1. Movement of child or camera (hold child’s head between knees of “helper”; stabilize camera with second hand)
7. Reflection artifact
   1. Move camera slightly between first and second photos to achieve different angle; gently dab conjunctiva with swab- must be done at periphery to avoid creating inflammation

### 7.2.6 Follow-up of Children

All children on the Master List of Eligible children for each community will be followed up at their household if they do not present for examination. Families will be offered free transportation to the examination site and back, or a home visit in order to assist in overcoming barriers to the examination. If the child/household is temporarily away, another appointment will be set up for a second day. If the child is too ill to be examined, refuses, has died or the household has moved permanently, or travel exceeds the time the study team can be in the community, then the next household and child from the alternate list will be selected to participate. When the alternate child is selected, the Master list will be updated to indicate which child is being replaced, and the Study Identification number of the replacement child (see Master List section 10.2.2) if additional data on the child or household is found that necessitates a change to the census list, then an update census form will be completed.

For the ancillary study in villages where all children ages less than ten years are enrolled, then efforts to include all children will be made as there are no “replacements”

## 7.3 Forms and Swab Management for Visits

At the end of the examination day, once all children on the Master List of eligible children and the alternates have been accounted for, three reconciliations will take place. First, the lab technician will be certain that each vial has a label. If one is missing, then the forms and specimen log, and vials should be matched to determine which label is missing. If more than one vial is missing a label, then the children may need to be re-swabbed to be certain the trachoma grade corresponds to the correct laboratory determination of infection. The number of vials must also match the number of ocular examination forms and specimen log. If there is a mismatch, then the vials and forms should be matched to determine what is missing. If an ocular examination form was missing or not filled in, the child must be re-examined. Similarly, if a vial is missing, and there is no notation that a specimen was not taken for a reason, then the child must be re-swabbed. Once the swabs, specimen log, and ocular forms are reconciled, the ocular forms are given to the photographer.

The photographer reviews his/her photo log to be certain that where a child was to have digital images of the eyelid that the photograph was taken or a notation made as to why the photograph was not taken.

The intake clerk will compare the ocular exams in hand to the Master List of eligible children to be certain that each child who presented was in fact examined. If an ocular form is missing but the child is listed as having presented, then the study team must return to the household and reconcile the difference, likely by re-examining the child.

The intake clerk must account for each child on the Master List of Eligible children for the community, either with an ocular form, a notation on the list (in which case there is an alternate child chosen); also if the notation states the child has died or permanently moved out of the community, or moved within households, then a census update form should also be present.

## 7.4 Missed Visits

Missed visits, for the purpose of this trial, occur if a community cannot be seen within the window of plus or minus two months. The four month window was chosen at it keeps the village visit largely during the same season as the original scheduled visit. Reasons why a community might not be able to be seen during its regularly scheduled visit include a funeral of a village leader, significant religious or country holiday, rainy weather, or a local reason advanced by community leaders. In any case, the events other than weather rarely last longer than a week in any country, so we expect no difficulties in re-scheduling visits within the window. The reason for the broad window is that other communities may have been notified of their visit schedule, and it is critical to honor those commitments, so a last minute re-schedule may take up to three weeks to re-do.

## 7.5 Follow-Up Visits

The first mass treatment is carried out immediately after the baseline survey. Subsequent follow-up visits are at 6, 12, 18, 24, 30, and 36 months from the first mass treatment. The census lists are updated formally every year prior to the 12, 24, or 36 month survey. The 6, 18, and 30 month surveys will use the census lists for the previous survey when drawing new random samples of sentinel children. The window for each survey is +/- two months.

The exact same procedures as stated for the baseline examination visit are to be followed for all subsequent examination visits. In all cases, mass treatment according to treatment arm is to follow immediately after the survey, to ensure that children are not treated prior to the survey.

## 7.6 Sample Study Visit Schedule

We show a sample schedule of study visits, in Table 2 for Tanzania where two teams are responsible for the surveys, and another team for mass treatment (which is described more fully in section 8). As noted earlier, we have two days in each community to schedule examinations. Depending on the month of starting, we have flexibility in the schedule to work around December holiday and rainy spells in January to April. Currently, only the 6, 18 and 30 month surveys are liable to be affected by the rainy season. If the rains are heavy, we will alter the schedule of surveys, and allow a window of +/- two months for the community examinations as described above. The six month examinations are key to the decision to mass treat in subsequent years, so they must be done.

Next, we show a sample schedule of study visits in Table 3 for Niger where three to four teams are responsible for the surveys, and another team for mass treatment (described more fully in section 8).

**Chapter 8: Intervention: Mass Treatment Implementation**

Chapter

8

_____________________________________________________________________

## 8.1 Overview: Treatment with Azithromycin

The intervention for this trial is community treatment with azithromycin, a single dose antibiotic highly effective against *Chlamydia trachomatis*. Antibiotic sufficient for this trial has been donated by Pfizer international to the National Trachoma Control programs in the three countries. The dosage is 20mg/kg, up to 1 gm in a single dose of either liquid (for children) or tablets (for children able to swallow tablets and adults). In Tanzania and Niger, pregnant women can be treated with azithromycin, but not in The Gambia. In The Gambia, Tanzania and Niger, children below 6 months are not treated with azithromycin but are given topical tetracycline for trachoma.

Azithromycin has been extensively studied for mass use in trachoma control, and has been found to be very effective and well–tolerated. In the ACT trial, no significant adverse effects in children and adults were noted in Tanzania, Egypt and The Gambia[[19]](#footnote-20). In the STAR trial, which treated adults, no differences in adverse events were noted between topical tetracycline and oral azithromycin[[20]](#footnote-21). Initial potential concerns for creating clinically significant resistance in other organisms to azithromycin do not appear to be realized[[21]](#footnote-22). On the basis of these and other studies, each of the countries in this trial have undertaken mass drug administration for their communities, with education to residents that any GI concerns be directed to local health centers and posts for treatment. We anticipate using the same local network to monitor any self reported adverse events, and this is described further in Chapter 14.

Below, we describe in detail the implementation of the intervention in each country setting.

## 8.2 Niger

Mass azithromycin administration in Niger will be carried out by trained community health workers based in each CSI and supervised by the Ministry of Health. (This team is not part of the survey/sample collection team.) The need for number of antibiotic doses will be determined by a grappe’s original census list. The census list will be updated at each treatment visit by community health workers.

A team of 8 health workers from the study area will be identified and recruited for training and distribution. Training will be 3-4 hours (refresher training may be more brief), and will include comprehensive information and practice in drug administration and enumeration/recording skills.

Before mass administration, trained health workers will meet with village chiefs to plan mass treatment for the community. Any questions regarding differences in treatment coverage, and/or exclusive treatment of children age 0-12 in the biannually treated arms (see below, section 8.2.1) will be addressed. Trachoma volunteers from each community will also be recruited by the health workers to help village leaders mobilize throughout the treatment phase.

In the enhanced (>90%) coverage arm, community members will be informed about the day(s) of mass treatment in their grappe a few days beforehand, and encouraged to participate in treatment. In the standard (80%) coverage arm, this community mobilization will not occur. The day of the treatment, health workers will go door-to-door, treating all members of each household. Participants’ names will be identified on the census list, and then verified as eligible to receive treatment according to address (and age, for bi-annual arm). Any known reason for non-attendance at mass treatment is noted on the list in pencil.

At the end of the day of mass treatment in all grappes, health workers will tally the number of individuals recorded on the census who have been treated. For those grappes assigned to the standard coverage arm, if 80% coverage has been achieved, no additional treatment days will be necessary. If 80% coverage has not been reached, one extra day will be scheduled and health workers will inform village chiefs and community members of the date of their return. After two visits, mass treatment will conclude for that community, regardless of the attained coverage. In the case of over or under treatment for that round, the community will still be analyzed on an “intent to treat” basis as described in Chapter 16, data analyses plan.

For those grappes assigned to the enhanced (>90% or more) coverage arm, up to three additional treatment days (for a total of four days) can be scheduled. Before returning for each follow-up day, health workers will compile a list of all individuals recorded on the census who did not yet receive mass treatment. Village leaders and trachoma volunteers will be given this updated list and asked to identify and mobilize those who missed treatment, informing them of the date of the return visit and encouraging their participation in follow-up visits. After four visits, mass treatment will conclude for that community, regardless of the attained coverage.

All those who present for treatment will be treated. This is true even in communities randomized to standard treatment, meaning that coverage could be as high or higher in communities randomized to standard treatment as those assigned to enhanced coverage.

### 8.2.1 Six monthly Mass Treatment (Niger)

Models imply that children form a core group for the transmission of trachoma—if infection can be eliminated in this age-group then it would presumably fade away in adults, whose treatment now requires a substantial portion of a program’s resources. We have demonstrated that treating children alone can be successful in an area with a modest amount of trachoma. A recent study in a hyperendemic area also demonstrates a degree of community protection in older, untreated individuals after quarterly treatment of children only.

Therefore, in addition to evaluating two coverage targets for the entire community, we will be evaluating whether biannual treatment of children is as effective as annual treatment of all individuals in a community. 12 grappes will be randomly assigned to a coverage level of 80%, and 12 other grappes to a coverage of ≥90%, with children ages 0-12 (i.e. newborns to those less than 13) receiving treatment every 6 months.

Biannual treatment visits will be organized and implemented in the same way described above (section 8.1.3), except that only children 0-12 will be treated in these communities (12 assigned to standard coverage and 12 assigned to enhanced coverage). Any questions regarding this exclusion category by community members will be clearly addressed and explained by health workers, who will have received training in this area. All children between the ages of 0-12 who present for treatment and have guardians present for consent will be treated.

8.3 Tanzania

Mass administration will be carried out and supervised by a special treatment team and a network of community health workers. This team is not part of the survey team, and has access to the randomization assignment of the village in terms of coverage. The survey team is masked to coverage assignment. The supervisor of the treatment team is an eye nurse, with three co–workers, working with community health workers. The Supervisor has a list of all community residents, based on the latest census update, and this will be used to estimate need for antibiotic and coverage.

An application is made to Tanzania National Trachoma Task Force for sufficient antibiotic to cover single doses for the 32 villages in this trial at each mass treatment round. The application covers liquid for children up to age four or so, then tablets (250 mg) for the remaining population. The supplies are brought to Kongwa by truck and stored in locked facilities at the KTP compound. A file of treatment assignment created by the study Biostatistician, Ms Munoz, is given to the supervisor to keep in a locked cabinet. As the treatment supervisor gets ready to plan for each mass treatment, s/he opens each envelope for the village and reads the treatment assignment.

After the survey days in the village, the treatment supervisor is meeting with the village leaders to plan mass treatment for the community. S/he explains the treatment assignment if it is other than the usual program (yearly with an 80% target). There are typically one to two community health workers in the community, and these are enlisted to help with distribution. The community will be informed about the days of mass treatment in their neighborhood (mtaa). At a central location for each mtaa, the mass treatment team leader will set up a station with his/her mass treatment list of residents in the village, and as each family comes, will provide a chit (ticket) for the medication, and check off that the persons within the family have come. If any family member is missing, the family is asked to make sure the member comes before the end of the day for treatment. The reasons for non-attendance at mass treatment are noted on the list in pencil.

Each resident has his own ticket with his ID number that is redeemed for a dose of the medication; the ticket is taken by the community health worker who supervises the dosing, and the dose is written on the ticket. The ticket is matched at the end of the day to the mass treatment list to be certain that those who showed up also had an observed dose of azithromycin, and assists in the accounting for drug dispensed for the national program. All mtaas will be provided a single day of mass treatment in their neighborhoods at the outset.

More treatment days will be scheduled depending on the tally of coverage at the end of the first set of days in each mtaa and the randomization assignment. For subsequent days, the community health worker will be notified in advance of persons who missed mass treatment, and will be asked to personally go and inform them of the subsequent treatment day in their community, and to advise treatment.

At the end of the day of mass treatment in all mtaas, the supervisor will tally the number of children ages 0-9 in the community who have been treated, using a specially built program that will determine the percentage relative to the randomization assignment. If, after the first round of mass treatment, the percentage of children is between 80% and 89% for the one assignment, or above 90% for the second assignment, then treatment for that community will stop. If treatment is below target, then the supervisor will schedule subsequent days for the community, with treatment to be held for ½ day in each mtaa. The tally will continue at the end of this second offering to determine if coverage has been met.

If, after the second offering of mass treatment the coverage has not been met, then the supervisor will schedule a door-to door offering for households with missing treatment data. The program in which the treatment data are entered will generate a random list of households and the resident(s) who remain untreated, with a cut-off for visits that will meet the target coverage. The community health workers will be given the random assignment of households to visit, with the names of persons missing treatment. If they are unable to find, or treat, persons to meet the target, then they keep going down the list until target is met.

All persons who present for treatment will be treated, even if it means that for communities randomized to 80-89% coverage, coverage will be higher than 89%. We cannot withhold treatment for those who have presented, as it will compromise the standing of the national Trachoma Task force and the community health workers.

If after one week of treatment scheduled as described above, the coverage is still below target, and the community health workers and treatment team report that all persons who could be were contacted and treatment refusal or prolonged travel accounted for missed doses, the supervisor will declare mass treatment ended for that community. In the case of over or under treatment for that round, the community will still be analyzed on an “intent to treat” basis as described in Chapter 16, data analyses plan.

In the experience of our team doing treatment, coverage of 80% can be achieved with usual offering for one or two days per mtaa. 90% can be achieved with special effort, which will be documented for cost effective analyses.

8.4 The Gambia

Mass administration will be carried out by community ophthalmic nurses based in each district and supervised by the National Eye Care Program Manager Mr. Sillah. This team is not part of the survey team, and has access to the randomization assignment of the district in terms of coverage. The survey team is masked to coverage assignment. The supervisor of the treatment team is a community ophthalmic nurse, working with community health workers. In keeping with program practice the *de facto* population of each settlement, as established the day before by enquiry is targeted for treatment. The list of all community residents, based on the latest census update will be used to estimate need for antibiotic and coverage.

The selected districts have been identified for mass treatment by The Gambia’s national plan for azithromycin in trachoma control, and sufficient antibiotic for 2008 is reserved at central stores under the control of Mr. Sillah. As the treatment supervisor gets ready to plan for each mass treatment, s/he opens each envelope for the village and reads the treatment assignment.

After the survey days in the village, a meeting is held with the village leaders to plan mass treatment for the community. If the treatment assignment is other than the usual program (yearly with an 80% target) this will be explained. There are typically one to two community health workers in the community, and a variable number of ‘friends of the eye’ (nyateros) and these are enlisted to help with distribution. The community will be informed about the day of mass treatment in their enumeration area (CEA). At a central location for each CEA the mass treatment team leader will set up a station with the census list of household residents, updated by enquiry the day before, and as each household comes, will provide a chit (ticket) for the medication, and check off that the persons within the family who are present in the village have come. If any family member is missing, the family is asked to make sure the member comes before the end of the day for treatment. The reasons for non-attendance at mass treatment are noted on the list in pencil.

Each resident listed in the census has his own ticket with his ID number that is redeemed for a dose of the medication; the ticket is taken by the community health worker who supervises the dosing, and the dose is written on the ticket. The ticket is matched at the end of the day to the census list to be certain that those who showed up also had an observed dose of azithromycin, and assists in the accounting for drug dispensed for the national program. Because it is program policy to treat the *de facto* population, IDs and tickets with separate numbering need to be reserved to account for the treatment of temporary visitors. All households will be provided a single day of mass treatment in their neighborhoods at the outset.

A second treatment day in each CEA will be scheduled in those districts randomized to enhanced coverage. For this day, the community health worker will be notified in advance of persons who missed mass treatment, and will be asked to personally go and inform them of the subsequent treatment day in their community, and to advise treatment.

At the end of the day of mass treatment in all CEAs, the supervisor will tally the number of children ages 0-9 in the community who have been treated, using a specially built program that will determine the percentage relative to the randomization assignment. All persons who present for treatment will be treated, even if it means that where communities are randomized to program coverage, their coverage will exceed that in communities randomized to enhanced coverage. We cannot withhold treatment from anyone who has presented, as it will compromise the standing of the national Trachoma Task force and the community health workers.

After either one or two visits, depending on randomization, the supervisor will declare mass treatment ended for that community regardless of whether coverage is still below target. In the case of over or under treatment for that round, the community will still be analyzed on an “intent to treat” basis as described in Chapter 16, data analyses plan.

In the experience of our team doing treatment, coverage of 80-90% of children in the *de facto* population is easily achieved at one visit and over 90% can be achieved with additional visit effort, which will be documented for cost effectiveness analyses. In the view of the program there is a rapidly diminishing return from more than two visits as people present but not presenting for treatment are likely to be refusals.

## 8.5 Monitoring Mass Treatment Coverage

The census data that are obtained and updated yearly will be the primary tool used for monitoring mass treatment coverage. The programs that are built for data entry of the census data will have a report that will be generated that consists of Mass treatment lists for each community, organized as needed for each country (for example, mtaa in Tanzania, grappe in Niger, and village in The Gambia). These master lists are used to record observed treatment coverage for each resident of the community. The data entry of these lists is essential to monitoring coverage as the treatment is underway.

Features of the program will be tailored to each country needs: for example, in Tanzania, as data are entered on coverage, a report can be generated calculating the coverage of children ages to 9.5 years, and if coverage has not hit target, a further report can be generated listing, in random order, all households with at least one resident who has not been treated, and the residents missing treatment. These lists can be used to further guide mass treatment steps as described above, and the data from these reports entered to update coverage. At the end of mass treatment, each community resident should be accounted for as having been treated or not treated and the reason why.

After each community has completed its mass treatment, the full data set on treatment is sent to the principal investigator for each country within a week in order to monitor the success of the treatment team in reaching coverage targets. S/he will review the percent coverage of the community, and if it is over or under target, the reasons why in the file. This will be done within three weeks of mass treatment ending so that if necessary, the treatment team can be re-deployed to the community to improve coverage. If all residents are accounted for, and the resident did not meet treatment coverage targets, the principal investigator will discuss with the treatment team supervisor ways to improve coverage for the second or third rounds (perhaps different timing or working with other community health team members, for example).

### 8.5.1 Mass Treatment Verification Procedure

Treatment verification is critical to ensuring that coverage levels have been achieved and that reported data is accurate. Treatment verification occurs after each community has completed mass treatment and is conducted by the Community Drug Distributers (CDDs) who initially treated the villages. Post-mass treatment, a CDD pair, consisting of a man and a woman for each sub-village, returns to the village to randomly select a sample of no less than five houses for interview. During the interview, the CDD pair records the number of people residing in the house and how many of those people received treatment on a treatment spot-check form. After the sample of houses is interviewed, the CDD pair compares the information recorded on the spot-check form to the data in the census treatment books as well as the information gathered from the household’s respondent during team members initial visits to the home prior to mass treatment.

8.6. Mass Treatment and Stopping Rule Implementation

In The Gambia and Tanzania, half the villages will be randomized to one year of mass treatment, and the data from the six month survey used to guide further treatment at the second year. Ideally, we would use the one year survey immediately prior to the next scheduled round of mass treatment, but the laboratory results cannot be processed that quickly.

The data from the survey on infection and trachoma in the random sample of 100 children ages five years and younger will be used to determine if the stopping rule has been met. The stopping rules are as follows:

1. If there is no infection in the sample, regardless of clinical trachoma, then the stopping rule has been met for that round of mass treatment and the community will not be treated.
2. If there is no evidence of TF in the sample, regardless of infection, then the stopping rule has been met for that round of mass treatment and the community will not be treated.
3. If either #1 or #2 has not been met, then the community will be mass treated again. Note that this means that if there is no evidence of infection, but still TF or TI, the community would be stopped. If there is evidence of infection, it would not be stopped.

The laboratories for The Gambia and Tanzania will process the specimens from the six month surveys, but as they are masked to treatment assignment, they will do all communities. They will report the data, and the study statisticians for Tanzania and The Gambia (Ms Munoz and Dr Bailey) will review each community’s data for evidence of infection or trachoma. The results –stop treatment or do not stop treatment-will be transmitted to the Treatment Team supervisor, and s/he will acknowledge receipt and place the decision document with the results in the file for each community. When it is time for mass treatment of the community, the treatment supervisor will review the decision document and plan accordingly.

In the data entry programs for entering mass treatment coverage, there is an “opt out” button for each community in the entry programs for The Gambia and Tanzania. Only the treatment supervisor can enter into that button with a pass word. A code will be present on the decision document. The supervisor will enter in the program that mass treatment was not carried out, and the reason was the result of the stopping rule; it will then prompt for the decision code, which must be entered to be valid.

Once a community has been stopped, it will not receive mass treatment again until after the third year survey, unless the 12 or 18 month survey show infection has climbed back to 20%, in which case mass treatment will be reinstituted at the next round. This parallels the WHO rule of re-surveying at three years to determine if further treatment is necessary. For example, if a community has no infection, as determined in the sentinel children, after the first round of mass treatment, the community will not receive mass treatment at the second or third rounds, even if infection starts to rebound, unless it rebounds to 20% by the 12 or 18 month survey. In this way, we are able to determine if, or with what rapidity, infection re-emerges. All communities will receive mass treatment after the last survey at three years, post baseline.

## 8.7 Masking Intervention

These trials are single masked, as it is not possible to mask the communities to the assignments they are to receive. There are two outcomes-clinical trachoma and infection-based on data collected by the survey teams. The teams that implement mass treatment are different than the teams that will be undertaking the survey. The survey team will be unaware of the assignment to coverage arm (80% versus 90%), and this element of the trial will be masked to the trachoma graders. However, for villages that stop (Tanzania and The Gambia) it will be difficult to assure masking of the survey teams, at least for the two and three year surveys. The survey teams enter the village before the treatment teams and are liable to be told by village leadership if they were stopped in the previous rounds. For villages in Niger randomized to target children for treatment, the survey teams are liable to be informed of treatment status by families.

The trial has several mechanisms to avoid bias in trachoma outcome assessment due to unmasking of assignment. First, trachoma assessment will be checked for consistency of each grader by reference to Dr Bailey, who will grade the images taken at each round. Dr Bailey will be masked to the treatment assignment in each country as he grades the images, which are identified by ID number only. If the grades are below kappa=.6 or show drift over time to lower grades, the grader will be suspended from surveys until s/he can be re-standardized. Second, a major outcome is laboratory evidence of infection, and the laboratory is masked to the treatment assignment of the specimen, so infection data are truly single masked. Finally, in the analyses phase of the study, we can evaluate the consistency of the relationship between clinical trachoma and infection across villages in the same treatment arm; if the relationship differs markedly, especially in the direction that favors drastic reduction in trachoma unsupported by the infection data, we will report that bias due to the fact that the unmasking may have been a factor.

# **Chapter 9: Cost Effectiveness Data Collection**

Chapter

9

________________________________________________

## 9.1 Overview

The cost effectiveness analysis will be based on community cost estimates that we will calculate using the cost collection instruments described below. For each community, we will collect cost information regarding: all medical and non-medical personnel involved in the study and all consumables used. We will also include laboratory costs attributable to screening/treating each community. The totality of the equipment costs - such as vehicles and field equipment costs – and laboratory costs will be recorded aggregately and then allocated to each community according to the community population.

The cost collection *per se* will be organized as follows:

For each field trip/intervention, a designated member of the team will fill out the recurrent costs forms. Equally, this designated member of the team will be in charge of filling out the capital costs that are only incurred once for resources shared across different communities.

The filled forms will be double entered using the data entry software “Access” by two different data entry clerks. The double-entered data will be verified, and any differences in data entry will be checked against the paper forms. The verified dataset will be sent to our health economist Mireia Jofre-Bonet who will analyze the data.

## 9.2 Cost Data Collection

Cost data to be collected are of two types: 1.Recurrent costs and 2.Capital costs – i.e. those only incurred once. The first kind of data will be recorded on each day in the field. The second type of costs data will be recorded aggregately for the study and allocated to each community according to its relative population. Details on filling out these forms are described in section 10.8

The cost data collection will be done by filling out the following five forms. These forms can be found in Appendix 1:

- Form 0: Capital Costs
- Form 1: Medical Personnel Costs
- Form 2: Non-Medical Personnel Costs
- Form 3: Field Consumables’ Costs
- Form 4: Lab Costs

# **Chapter 10: Study Instruments**

Chapter

10

______________________________________________

## 10.1 Overview

The data to be collected for this study consists of household information, and clinical and laboratory information on the child. In addition, data are collected on the mass treatment intervention, and details of the study process. In the next section, we describe the forms that are used to collect these data. These forms can be found in Appendix 1.

## 10.2 Census Forms

Overview: The baseline census form is used to collect information on each household in the communities randomized to this trial. It is the central document that provides information for the following:

- Selection of households with eligible children in the community
- Selection of children within a household when there is more than one eligible child
- Master List for Surveys
- Data base for mass treatment
- Environmental factors at the household level that may be important for trachoma

Description of how the census is to be conducted, and appropriate numbering of doorways is described in Chapter 6 for this study. A household is defined in Tanzania as a unique doorway belonging to a family. In Niger, a household is a family unit with one male head of household and often more than one wife (up to four wives), sleeping regularly in the same compound. In The Gambia, a household is a group of people who eat from a single cooking pot. The definition of resident in the household means a person who has slept in this household for at least three month in the past (or if age less than 3 months, was born not the household) or who intends to reside with the family for the next six months. In this section, details are provided on filling in the Baseline and Update Census forms. This section should be used with the forms in Appendix One (Study Forms).

### 10.2.1 Baseline Census Form (Form 1)

Identification numbers:

GRAPPE/EA/VILLAGE__ ___

In Niger, use the first two numeric digit entries to identify the grappe; In The Gambia, use the entry to identify the Enumeration Area; for Tanzania, use the entry to identify the village.

VILLAGE/MTAA: __ ___

In Niger and The Gambia, use the second numeric two digit entry code to identify the village; in Tanzania, use the entry to identify the mtaa.

HOUSEHOLD NUMBER: ___ ___ ___

Use this three digit alphanumeric entry to uniquely identify a household; this number is also written on the door of the household.

Village Name: write in the name of the village

Name of head of Household: Write in the name of the head of the household. This may be provided by the neighborhood leader or another neighbor if the head or another adult in the family is not home.

Census taker: insert the initials of the census taker for this household. Each census taker should have a unique set of initials, including a number if there is more than one person with the same initials.

Date: insert the date in which the household census was completed (or the date at which census gathering had to be stopped and no information was provided). Use DD/MM/YY format.

1. Is there an adult home to answer questions?

If yes, mark “yes”. If not, the census taker should determine when would be a better time to return to get the information. If the family is traveling and not returning during the time of the census, then the census taker must indicate the reason for being unable to census. If there is not information on the family, s/he MUST return at least three times at different times over the course of the village census to try and catch an adult at home. If the census taker is unsuccessful, then mark “no” unable to census and provide a reason why. This household will not be chosen as a source of sentinel children, as detailed information will not be available on the children, but census information may be provided at the time of mass treatment, if the family returns for that activity.

2. How many years of education has the head of household completed?

Ask the question of the adult, and make sure that the answer is years completed, not including starting but not completed. Write in the number of years. If the answer is a grade, translate that into years completed. For example, in Tanzania the answer maybe Form one, in which case eight years of education has been completed. If the adult does not know, write letter “X for unknown.

3. How far away is the nearest water source?

We will measure the distance to water by asking how long it takes to walk one way directly to the nearest source of water. Time will be measured relative to the time it takes to do common tasks, such as boil water, cook ugali, or other tasks. Once the persons can provide an estimate, code as either less than 30 minutes, 30 minutes to one hour, or more than one hour. Each country will have its own code of relevant tasks that estimate time.

4. Has there been a health education program to promote face washing in this village in the last year? I do not mean a radio campaign but a local program in your village.

This is an optional question for areas in which the full SAFE strategy has been implemented. It helps determine if the health education campaigns have reached the village level.

5. OBSERVE: Does this household have a latrine:

The census taker will look in the compound or area around the house for a latrine. If one is not visible, the census taker will ask the interviewee if the household has a latrine. If yes, the team member will ask to see it. If it is within 60 feet (20m) of the house, it will be counted as a latrine for that household.

6. OBSERVE: Is the doorway area clear of garbage or waste?

The census taker observes the cleanliness around the doorway of the house. S/he will be standardized to observe the area around the doorway for evidence on the ground of leftover food or animal or human waste. Waste water or plant detritus does not count as waste. If there is a large animal pen within 25 feet (about 8 m) of the doorway, this will be recorded and counted as unclean doorway.

7. Household List: note that to the left of each name slot is a pre-printed number in sequential order. This two digit code is the unique person code for each person in the household. Use it when needed to identify the mothers of children as described below.

Name: List the names of all persons who reside in this household, as defined above. Start with the head of household. List the first name, last name, and an optional place for alias if needed.

Sex: Note the sex of the person as “M” for male and “F” for female.

Date of Birth: If it is possible to obtain a date of birth, record the date of birth: use DD/MM/YY format. If only the year is known, fill in “x” for the unknown numbers. Use a vaccination card or MCH card if available to get the birthdate of children, and a voting card or other piece of ID for the adults if available. If the entire date is unknown, then skip to the column labeled “age”

Age: use this column if no birthdate, or at least year of birth, can be obtained. Use an events calendar specific for each region to estimate the age of the person. If necessary, have other family members assist in determining the age. Avoid having a consensus using round numbers or last digits with 5’s as it indicates digit preference; try to get the person and/or family members to b e as precise as possible. For children age less than ten, indicate years and months if possible. For children less than age one year, insert a zero for years.

Mom: For each child age less than ten years, insert the two digit person code for the primary caretaker of the child. This will help identify the child in subsequent surveys.

Child Face status: The census taker will make observations on the cleanliness status of each child ages five years and under who are present at the time of the census. S/he will be standardized to measure a clean face as the absence of ocular discharge or “sleep” on the eyelashes or lids, the absence of nasal discharge on the nares and cheeks and lips, and the absence of flies on the face when observed for 3 seconds. The presence of any one sign is evidence of an unclean face. If the child is not present or cannot be observed, indicate “X” for each sign as “don’t know”. Otherwise, record yes or no for presence of sign on the child’s face.

Random number: an optional column for study sites where the data entry cannot be completed prior to selecting an eligible child in a household, and a random number assignment is made in the field. In the field, the census takers will have a Table of random numbers with a random start. They will then go horizontally across the page as they assign each child, starting with the first closest to the top, a random number in order that they appear on the table. As the number is assigned, it is crossed off the table. The child with the lowest number is the one selected from that household. For example, if child 04 has number 54 and child 05 has number 16, child 05 is the selected child. Note that if child 05 is not available for the survey, another household entirely is selected, not another child from the same household. This avoids always selecting younger children as they tend to be around the house.

### 10.2.2 Census Update Forms

#### 10.2.2.1: Surveys and mass treatment updates (Form 2)

After the baseline census, surveys will be undertaken and mass treatment provided. Each of these contacts provides an opportunity to update the census information for the village. The Master List of eligible children for the survey has a place to note if the child has died or moved permanently from the village (see section 10.2.3). If these are the reasons the child was not examined, then a census update form is generated for data entry. Similarly, if a resident has been added to a household at the time of mass treatment (who is not a visitor), or a resident has died or moved, this information is noted in the mass treatment book and generates a census update form. The Census update asks for the complete identifying number of the person. This is the Grappe/EA/Village number, village/mtaa number, household number and person number. Assign the next available number in the household to the person to be added.

If the person is to be added: fill in the information on the new addition exactly as described from the baseline census:

Name: List the names of all persons who reside in this household, as defined above. Start with the head of household. List the first name, last name, and an optional place for alias if needed.

Sex: Note the sex of the person as “M” for male and “F” for female.

Date of Birth: If it is possible to obtain a date of birth, record the date of birth: use DD/MM/YY format. If only the year is known, fill in “x” for the unknown numbers. Use a vaccination card or MCH card if available to get the birthdate of children, and a voting card or other piece of ID for the adults if available. If the entire date is unknown, then skip to the column labeled “age”.

Age: use this column if no birthdate, or at least year of birth, can be obtained. Use an events calendar specific for each region to estimate the age of the person. If necessary, have other family members assist in determining the age. Avoid having a consensus using round numbers or last digits with 5’s as it indicates digit preference; try to get the person and/or family members to b e as precise as possible. For children age less than ten, indicate years and months if possible. For children less than age one year, insert a zero for years.

Mom: For each child age less than ten years, insert the two digit person code for the primary caretaker of the child. This will help identify the child in subsequent surveys.

Note for new additions if they are children, the face status is not needed.

If subtracting a person from a house: check that the person whose ID is listed is to be subtracted from the household and provide a reason. If the person is known to have moved to another household in the village, check #3, and provide the household number where the person has moved. If the household number is unknown, provide the name of the head of the household and as soon as possible, locate the household number for that head of household. Generate another census update form to add those persons to their new household. If the reason for subtraction is not death or re-location, check “other” and specify the reason. If the person is in the village but refused to be surveyed or mass treated, DO NOT fill in a census update form. This is only to be used if the location has changed.

If subtracting an entire household from the census, you do not need to insert a person number, but item #3 on change code must be checked. Provide a reason for the subtraction. The result will be the elimination of that household number, so if the entire household has become part of another household (reason #2) then be sure and have the number of the other household, and add the persons to the household with another census update form.

#### 10.2.2.2 Yearly Census updates (Form 3)

The census will be updated every year in preparation for drawing a new sample, and undertaking mass treatment. A Master Census list of each house will be printed, using the most recent census update data, likely from the six month survey previous. The census takers will use this book to return to each house and update the census.

If adding new persons, use the same information as in the baseline census form:

Name: List the names of all persons who reside in this household, as defined above. Start with the head of household. List the first name, last name, and an optional place for alias if needed.

Sex: Note the sex of the person as “M” for male and “F” for female.

Date of Birth: If it is possible to obtain a date of birth, record the date of birth: use DD/MM/YY format. If only the year is known, fill in “x” for the unknown numbers. Use a vaccination card or MCH card if available to get the birthdate of children, and a voting card or other piece of ID for the adults if available. If the entire date is unknown, then skip to the column labeled “age”

Age: use this column if no birthdate, or at least year of birth, can be obtained. Use an events calendar specific for each region to estimate the age of the person. If necessary, have other family members assist in determining the age. Avoid having a consensus using round numbers or last digits with 5’s as it indicates digit preference; try to get the person and/or family members to b e as precise as possible. For children age less than ten, indicate years and months if possible. For children less than age one year, insert a zero for years.

Mom: For each child age less than ten years, insert the two digit person code for the primary caretaker of the child. This will help identify the child in subsequent surveys.

If subtracting a person, indicate the reason as described above: If the person is known to have moved to another household in the village, check #3, and provide the household number where the person has moved. If the household number is unknown, provide the name of the head of the household and as soon as possible, locate the household number for that head of household. Generate another census update form to add those persons to their new household. If the reason for subtraction is not death or re-location, check “other” and specify the reason.

If the entire household has departed the village, then note the household has left permanently. There should be tangible evidence of permanent re-location and not just traveling. Temporary absences of households of less than one year should not be changed in the census unless the neighbors/village leaders know they have gone for good.

In the event that a new household has come into the village, then the census taker should assign a new household ID (use the next available household number on the village list being certain that it has not been assigned elsewhere). Add the number to the doorway. Fill in a census form identical to the baseline census form with household information noted as described above.

### 10.2.3 Master List of Eligible Children for Survey (Form 4)

As the examination surveys are undertaken, the intake clerk will have a master list of children who have been randomly selected for the surveys (Form 4). On the list is a column that allows reasons to be given for why children are not examined. If a child is not examined due to death or a permanent move out of the village, then a census update form is generated by the intake clerk, and that form is entered into the census data base and updates the census files.

## 10.3 Ocular Form (Form 5)

The top of the ocular form is pre-printed with information from the census data base. There should also be affixed a set of four labels. These labels are used for the specimen vial, the specimen log book, and the photo log book if necessary and the field control swab if necessary. The examiner should observe the form for a star in the upper right corner, which indicates the child needs an ocular photograph of the right eye.

Child Study ID number: the nine digit code consists of a two digit village/grappe/EA code followed by the two digit village/mtaa code, followed by the household number followed by the person code.

Examiner: the person who will be doing the trachoma grading should insert initials. These initials will be checked against a data base of certified graders.

Date: the date the exam was performed should be written in. The format is DD/MM/YYYY

Child’s name: the child’s name as it appears in the census database will be printed here.

Age: the child’s age as it appears in the census database will be printed here.

Sex: the child’s sex as it appears in the census data base will be printed here.

Head of Household name: the name of the head of the household where the child resides is listed here as it appears in the census data base.

(NOTE: The age and sex help identify the child that should be examined, in case there is any confusion. The head of the household name is useful to locate the child or perform a house visit if necessary)

1. Was the ocular exam performed on the child?

The appropriate box is checked. If the child was present but no exam was done, a reason should be specified. If answers 2-5 are checked, then the first child off the alternate list should be informed and brought in for examination. If answers 4 or 5 were checked, then a census update form also needs to be filled out to ensure the census data base is updated.

1. Classification of trachoma

The examiner first examines the LEFT EYE. S/he grades the eye for trichiasis and corneal opacity. The eyelid is then flipped and signs of TF, TI, and TS are graded as present: “1” or absent: “0”. If they cannot be graded at all, use grade “9” for cannot grade.

The examiner then examines the RIGHT eye. The grading is repeated. The examiner inserts “0” or “1” as necessary. No space should be left blank-if the sign is not present, a “0” must be written to be certain the sign was not skipped.

1. Was a swab taken?

A swab must be taken of the eyelid of the right eye (if the child is selected for photography, then the image is taken prior to swabbing). If yes, then the examiner must write in the box number in which the vial is placed, and place an ID label next to the box number. The label helps to make certain the vials are not mixed up. If the swab is not taken a reason must be provided, and a child from the alternate list is informed and brought in for examination.

1. Was this child chosen for photography?

If yes, then tick off the yes box and place the ID label next to the yes box and another label in the photography log book. The first image taken is of this identification number in the photography log book.

1. Was this child chosen for a field control lab swab?

If a blue colored swab was selected for this child, then the child is to have an air swab taken. Procedures for the field control are described in section 7.2.2. In this case, a special set of field labels have been created which resemble regular identification numbers but are laboratory controls. Place one of these triplicate labels next to the yes box, to link the child to the field control; place the second label on the vial, and place the third on the specimen shipping list for the box in which it will be inserted.

## 10.4 Photo Log Book (Form 6)

The purpose of the photo log book is to keep a record of the photograph images taken as backup in case there is an error in the order the images are taken, or the ID photo is missing. The photo log book consists of sequentially numbered columns. For each person to be photographed, the ID label with the form is placed in the next available slot, and the image is taken of that identification number. This link enables the clinical grade to be associated with the photograph grade. Then the photographer takes two images of the upper eye lid as described in section 7.2.3. S/he reviews the images, and if they are acceptable, crosses off the next two slots after the ID label in the photo log. If one or both is not acceptable, then one or more are deleted and new images taken until there are at least two that are acceptable for transmittal. There should be an “x” for as many images as are going to be transmitted.

At the end of the survey, the photographer burns the 150 plus images (50 children times a minimum of three images) onto a CD and sends the CD to Dr Bailey, with a copy of the photo log for that survey period.

## 10.5 Ocular Specimen Shipping List (Form 7)

Each of the specimens taken will be placed in a box for the laboratory that holds 81 vials (100 tubes in Niger). The boxes are numbered sequentially, and indicate the Grappe/EA/village and date it is filled. As the specimens are taken, a label is placed on the ocular form, on the vial, and on the shipping list. Each page of the shipping list refers to a separate box, so that each page can serve as the list sent with the specimens to the lab to describe the contents of the box. In addition, the box number is indicated on the ocular form, so if a vial is missing, the likely box in which it was packed can be located.

When the page for a box is completed, the page is copied to send with the box when shipped. And the original is kept with the field team as a record of having taken and packed the vial.

## 10.6 Mass Treatment Log Book (Form 8)

This book is printed out from the census data base at the time of mass treatment, for use by the mass treatment team to determine coverage. Each page is a unique household in the community, organized by village or mtaa and last name of the head of household. Section I is the listing of the current residents. For each person, indicate with a tic in the appropriate box if they were treated or not, and if not present, why they were not present. If they were treated, then indicate the dose that was provided in terms of number of pills or ml of liquid.

Section II permits adding new residents and indicating if they are temporary residents of the household for the purposes of mass treatment. Sometimes the village is inflated with persons who want treatment, and indicate they are part of a household in case that is a requirement, which in this study it is not for purposes of mass treatment. By adding them and indicating they may be just temporary, it helps account for the doses used but does not artificially inflate the census or coverage figures.

This book is entered into the data base as a record of coverage for the community, and if new persons have come into the house as permanent residents, then the census is also updated.

## 10.7 Stopping Rule: Decision Report Form (Form 9)

The Data Coordinating Centers for Tanzania (JHU) and The Gambia (LSHTM) will receive the results from the laboratory processing of specimens at 6, 18, and 30 months. They will also have the clinical grades for the sentinel children. The laboratory results show no child of the 100 has infection OR that no child has TF, then the community has met the stopping rule, and the CC issues a report to the head of the treatment team that the community is not to be treated at the next scheduled mass treatments. The report will also contain a code for that village that can be used by the treatment supervision when entering in the data on the reason why the village was not treated. If there is at least one child with infection then the stopping rule has not been met and the report will indicate that treatment is to continue for that community. The report is also sent to the senior principal investigator for each clinical site. However, the report is not sent to the head of the survey team, as they are not to be informed of the community treatment arms.

The report is a simple indication of the community name, the outcome of the survey, and the action, with a code if necessary, for use by the treatment team supervisor to verify why the village was not treated.

## 10.8 Cost Forms

*Form 0: Capital Costs*

This form contains cost information on any vehicle used during the study and field costs incurred only once. This table will be filled out only once and not for every field trip. Capital costs will be imputed to each village on a population based proportional basis at the end of the study.

The table referring to capital costs for vehicles:

1. Average petrol cost per kilometer/miles: Record the cost of petrol, in the currency of the country, at the beginning and end of the field trial. Add these 2 values together and divide by 2 to get the average cost. Conversions into US$ will be performed at the end of the study.
2. State whether the car was bought or rented
3. State the purchase price, in the currency of the country, when applicable.
4. State the daily cost, in the currency of the country, of the vehicle if it is rented
5. State the years of useful life attributable to the car. This will be calculated based on the year of make of the car and the number of kilometers ran by the car.
6. State the resale value of the vehicle (if it is not resold at the end of the study, state what the likely resale value would be).

The table referring to field equipment capital costs:

For each item of equipment:

1. The number of units
2. The price/unit in the currency of the country
3. State whether the good was donated or purchased
4. If the good is donated, state what its market value would be.

*Form 1: Medical Personnel Costs*

Form 1 will be filled out for every day in the field.

At the top of the form:

1. The date of fieldwork will be recorded in the format: “1^st^ January 2008” (to avoid American MMDDYY versus British DDMMYY formatting).
2. The location(s) of fieldwork (the name of the community/communities worked in)
3. The activity/activities performed that day (e.g. sensitization, census, examination, treatment)
4. State clearly the number of people covered by the activity e.g. “100 children examined”, “150 people treated”.
5. The distance traveled (state Km or miles) that day by each vehicle separately

Medical personnel table:

1. The position of the medical personnel involved (e.g., ophthalmic nurses). Each person is written on a separate row
2. The number of hours spent working that day (including travel to and from location from accommodation)
3. The tasks performed that day by that person
4. The person’s normal salary and the over what time unit (e.g. salary/year), in the currency of the country
5. If , and how much (in the currency of the country), the person received as a per diem
6. Any additional costs for that person (e.g. accommodation, food etc.).
7. Any notes that are relevant to understand the extent of the involvement of that person in the study.

*Form 2: Non- Medical Personnel Costs*

Form 2 will be filled out for every day in the field.

At the top of the form:

1. The date of fieldwork will be recorded in the format: “1^st^ January 2008” (to avoid American MMDDYY versus British DDMMYY formatting).
2. The location(s) of fieldwork (the name of the community/communities worked in)
3. The activity/activities performed that day (e.g. sensitization, census, examination, treatment)
4. State clearly the number of people covered by the activity e.g. “100 children examined”, “150 people treated”.
5. The distance traveled (state Km or miles) that day by each vehicle separately

Non-medical personnel table:

1. The position of the non-medical personnel involved (local helpers, drivers, etc). Each person is written on a separate row
2. The number of hours spent working that day (including travel to and from location from accommodation)
3. The tasks performed that day by that person
4. The person’s normal salary and the over what time unit (e.g. salary/year), in the currency of the country
5. If , and how much (in the currency of the country), the person received as a per diem
6. Any additional costs for that person (e.g. accommodation, food etc.)
7. Any notes that are relevant to understand the extent of the involvement of that person in the study.

*Form 3: Field Consumables Costs*

Form 3 will be filled out for every day in the field.

At the top of the form:

1. The date of fieldwork will be recorded in the format: “1^st^ January 2008” (to avoid American MMDDYY versus British DDMMYY formatting)
2. The location(s) of fieldwork (the name of the community/communities worked in)
3. The activity/activities performed that day (e.g. sensitization, census, examination, treatment)
4. State clearly the number of people covered by the activity e.g. “100 children examined”, “150 people treated”
5. The distance traveled (state Km or miles) that day by each vehicle separately

Field consumables table:

1. The name of the consumable (one consumable per row). These items may include: tables, powder, water if bought, dispensing instruments, swabs, gloves, etc. Note that vehicle repairs are recorded here.
2. The cost/unit (in the currency of the country), plus specify what the unit is e.g. “Box of 100 gloves”, “Box of 100 swabs”
3. How many units were used
4. State whether the goods were donated or purchased
5. If the good is donated, state what its market value would be
6. Any relevant notes that can clarify the market value attributed to the good when controversial.

*Form 4: Lab Costs for PCR*

Form 4 will be filled out for every day of PCR lab work.

Capital costs table:

For each item of equipment (e.g. freezer, refrigerator, thermocycler, safety cabinet, pipettes, heating blocks, vortex, tube racks, plate washer, incubator, Microwell Reader, computer, printer):

1. The number of units bought
2. The price/unit in the currency in which they were bought
3. State whether the good was donated or purchased
4. If the good is donated, state what its market value would be

Lab staff costs table:

To be filled in every day:

1. The position of the lab staff member (e.g. lab technician). Each person is written on a separate row
2. The number of hours spent working that day on the study’s samples - excluding the lunch break if it lasts more than half an hour
3. The tasks performed that day by that person for PCR of the study’s samples
4. The person’s normal salary and the over what time unit (e.g. salary/year), in the currency of the country
5. Any additional costs (accommodation, food, per diem) per day
6. Any notes that are relevant to understand the extent of the involvement of that person in the study.

Lab consumables costs table:

1. The name of the consumable (one consumable per row). These items may include: tips, gloves, tubes, kits, labels, plate lids etc.)
2. The cost/unit (in the currency of the country), plus specify what the unit is e.g. “Box of 100 gloves”, “Box of 100 P200 tips”
3. How many units were used
4. State whether the goods were donated or purchased
5. If the good is donated, state what its market value would be.
6. Any relevant notes that can help clarify the market value attributed to the good in question.

# **Chapter 11: Training and Certification**

Chapter

11

_______________________________________________________

The PRET partners will hold an eight day workshop prior to the start of the study, where the teams from The Gambia, Tanzania, and Ethiopia will get together for extensive training, standardization, and certification in study protocols and procedures prior to the start of the trial. This workshop is part of the quality assurance mechanisms for this trial, and assures that all teams are performing in an identical fashion, and to protocol. UCSF personnel, Drs Lietman and Gaynor, who were previously certified as standardized graders during the original training, trained and tested the PNLCC team when the study site moved from Ethiopia to Niger.)

## 11.1 Trachoma Grading

The standard for this trial will be the WHO simplified grading scheme, consisting of follicular trachoma (TF), trachoma intense (TI), and trachomatous scarring (TS). Dr Robin Bailey will be the senior standard grader against whom all the trachoma grading will be compared for quality assurance purposes. He will conduct a training session which includes the WHO training set, supplemented by slides to teach the characterization of trachoma and other signs as seen in the field. After verbal discussion and agreement with the slides, the teams are taken to the field in Niger, for field experience grading, and a standard trial.

Field experience consists of graders observing eyelids together and agreeing on the grade, followed by one on one with Dr Bailey, discussing eye lids clinical signs. When Dr Bailey feels the trachoma graders are sufficiently standardized, then we will have a formal trial, where 50 children are all graded by each grader, masked to the grades of the others. The children walk, with a number attached to each shirt, to each grader who will assess the presence and absence of each sign. At the end, the score sheets are entered and kappa assessed for each grader compared to Dr Bailey.

If the kappa is 0.6 or greater, then the grader is certified for the trial. If the kappa is worse, then the grader must do more work with Dr Bailey to achieve a better agreement. No grader can be part of the data collection for the trial unless certified.

If, during the course of the trial itself, the review of photographs indicates that a grader is drifting, and the kappa for his clinical grades against his photogrades as read by Dr Bailey is less than 0.6, then the grader receives suspension of certification subject to re-training. The re-training can be done in country by another grader who is still certified. Once that grader and the suspended grader have carried out a trial in masked fashion, and agree with kappa =0.6, then the suspended grader can be reinstated provisionally. At the next clinical grading session, he must be certified again by Dr Bailey’s review of the grades of the photographs compared to the clinical grades.

## 11.2 Laboratory Specimen Handling

The standard for this trial are the protocols developed and used by the University of California at San Francisco. They are described in detail in section 7.2.2. During the standardization workshop, Dr Lietman’s team will conduct training on appropriate laboratory procedures. A session where the protocol is described and details provided will be held. This will be followed by a field session where those who will be taking specimens will be observed for proper technique, from opening the packages, swabbing the eyelid and inserting the swab in the vial, and labeling. Open discussion of technique will take place for the first and possibly second attempts. The lab technician will need to perform properly on two eyelids to be certified for the trial. No lab technician can be part of the data collection for the trial unless certified.

Throughout the trial, air swabs are taken to check for possible field contamination. If any of the swabs are positive, then the lab technician receives provisionally certification, subject to review by another, certified, lab technician in country. Once re-training is accomplished, the lab technician is re-certified, subject to review again of his field control swabs.

## 11.3 Photography

Each person who will be doing photography for the trial will receive training from Drs Bruce Gaynor and Tom Lietman on the proper use of the handheld Nikon D-series with macro lens to achieve gradable images of the upper lid. Training will consist of orientation to the camera, its parts, and care of the camera. Procedures will be taught that include shooting the identification number first, labeling the photo log with the patient identification number, and downloading and storing images on a CD for transmittal to Dr Bailey AND to the study site investigator.

Participants will practice shooting images of each other’s eye lids until acceptable images are obtained, while shooting within a few seconds. Once the trainers feel the photographers are sufficiently ready, they will take their equipment to the field and while the trachoma graders/laboratory technicians are practicing in their sessions with trachoma cases, the photographers will shoot images. (In the event that the photographer is the same person as the lab tech or trachoma grader, then the photographer will practice on his/her own cases).

Certification will be granted once photographers pass an oral test on camera care, trouble shooting, and procedure, and can submit three consecutive acceptable photographs, with ID numbers, from the field, downloaded onto a CD. Photographers who do not pass this certification cannot be used in the trial.

## 11.4 Study Forms

The standardization workshop will include a session on the study forms, and the training and certification will be led by Ms Munoz, lead statistician and data manager for PRET. She and the economist from London will conduct a formal session going over in detail the forms for all participants at the workshop, so all are familiar with each form, each field and how to fill it out properly. The session will include practice session with other team members posing as head of households for census, and practice with census updates, mass treatment, and examination forms.

In the field, those who are doing trachoma grading will practice filling in the ocular forms, the cost forms, and the mass treatment forms. Those supervising census will practice on households in the village with census forms and updates. Standardization exercises will be led by Dr West on observations of a clean face, presence of a latrine, and observations of garbage and waste around the doorway. The team leaders of each country will be expected to teach their census teams these observations once they have been standardized at the workshop.

Each study team member from each country must undertake an oral test on filling in the forms properly and how to handle problems and each must demonstrate acceptable performance in filling in each of the forms, as observed by Ms Munoz and the economist. Ms Munoz will then certify the team member on the study forms. The certified team members are then required to teach, using the same methods, the team members for their country team and issue certification. Lists of the team members who have successfully passed certification will be sent to each study site investigator and to the Executive committee. Only the initials of certified study team members will be allowed on forms sent for data entry. Only team members who have been trained and certified by the country study team member from the workshop can use study forms for this study.

## 11.5 Data entry

Data entry managers will be included in the Workshop. They will be part of the session on study forms, to understand details on the content and completion requirements for the forms. They will then attend sessions on data entry programs for the study that will cover the following topics:

- How to maneuver through the data bases
- How to do data entry for each form
- Double data entry
- How to troubleshoot problem forms
- When to send forms back to the field for resolution
- Meaning of error messages and warnings
- Saving data bases
- Copying data bases for back up and encryption
- Printing reports
- Signing off and proper storage of paper forms
- Tips on Access

The data bases will have a manual associated with them for use by the statisticians and data managers for each country site.

The data managers must be certified by Mr. Dreger, lead programmer for JHU, on data entry of all forms. Certification will consist of successful entry of a set of practice forms, saving and copying data bases, and proper sign off on forms. Once certified, the data manager can then train their data entry team members. All certified data entry team members must have their names sent to the study site investigators and the Executive committee members. Only data entry persons who have been trained and certified will be permitted to do data entry for this study, and their initials will be recognized for data entry.

# **Chapter 12: Data Management and Routine Reports**

Chapter

12

_______________________________________________________

## 12.1 Overview

Well-designed data management infrastructure is an essential part of any field study, given the large number of people and places involved in the data collection process. To ensure appropriate data management, one person must be responsible for the supervision of data collection, processing, and/or storage. For this project, the study coordinator will be responsible for the supervision of data collection at the field sites during baseline and subsequent follow-up visits. Once the team has finished the field work in a community, the study coordinator will supervise data entry and management at the central office in each study site. The study coordinator will have ultimate responsibility for regularly checking with each individual to evaluate data flow.

All study forms were created in English and they will be translated to the local languages if needed. The process of translating forms is a multi-stage process that requires a minimum of two individuals fluent in both English and the local language. One individual will translate all forms into the local language. Once the translation is complete, the forms will then be given to a second translator who will translate the forms back into English. Both translators will review the forms for cultural sensitivity and understanding, and will report any problems to the principal investigator. The newly translated English-language forms will then be compared to the original forms to determine any differences that arose during the translation process. If corrections need to be made, the principal investigator will meet with the first translator to discuss how the differences arose and what changes need to be made to correct these differences. The first translator will then make the changes to the local language forms and give them to the second translator, who will again translate the forms into English. This process will continue until all participants in the translation process are satisfied that the local language and English language forms are interpreted in the same manner.

## 12.2 Niger

### 12.2.1 Study Forms Preparation

#### 12.2.1.1 Census Forms

At baseline, each census taker is given a set of forms for a grappe, and a list of household numbers unique to them. The numbers may begin with a unique letter, then sequential numbers. At subsequent census, a master Census Book is created with the most recent version of the census for that grappe, which is used to update the census with new arrivals and departures.

#### 12.2.1.2 Master Survey List

An electronic copy of the original census document is an electronic copy of the original census document. Using an electronic copy of the census, 120 children are randomly chosen. These children are listed on a Master Survey List, containing the full spelling of each individual’s name, as well as their age, gender, and household number. A new Master Survey List is generated for each grappe at each collection visit and is used for both mobilization and registration.

#### 12.2.1.3 Ocular Examination Form

The Examination Form is printed for each of the 120 children who will be part of the sample survey in each grappe. Random number labels are also pre-printed for placing on tubes and examination forms.

#### 12.2.1.4 Blank census update forms

Blank forms are brought each day to the field during surveys or mass treatment in case they are needed to update information on sample children or residents.

### 12.2.2 Daily Data Management Activities in Field

All completed Master Survey Lists, Census update forms, Ocular Examination Forms and, depending on the activity, mass Treatment or Census books will be given to the Study Coordinator at the end of each workday and stored in a safe, secure place for transport to Niamey at the visit’s conclusion. The Study Coordinator will check the forms for completeness and accuracy.

### 12.2.3 Data entry

The census, collection fieldwork and treatment data collected in the field will be entered into a database at the World Vision office in Maradi, Niger. A double entry on key fields, using the uniform Access data entry package created for each site, will be done to minimize data entry errors. The PNLCC data manager will do the final editing. The Proctor Database Manager will enter all PCR results into the database.

### 12.2.4 Preparation of Data for Transfer to UCSF

#### 12.2.4.1 Data management

All treatment and collection data will be entered within four working weeks of collection. Data will be entered and inconsistencies will be resolved by the respective study coordinator or, if necessary, by consultation with the appropriate data collector. Access to any patient information will be protected by a password, or locked in a secure storage room. All data will be backed up upon entry, and forms will be kept until the conclusion of the study.

#### 12.2.4.2 Reconciliation of data

Before data entry, the forms will be reviewed and cross-checked for consistency and completeness. If the forms are not filled out completely, the PNLCC data manager will contact the person responsible for completing the form to provide missing data or clarify any inconsistent data. The data manager is the only person who is authorized to add missing data or make any changes to the study forms. All changes will be made in a different color ink and initialed and dated.

#### 12.2.4.3 Specimen transfer procedures

In accordance with the Roche COBAS AMPLICOR™ CT/NG protocol, swab samples taken in the field will be transported on ice in a closed, insulated container until arrival in San Francisco, where they will be stored at -80°C for later analysis. Samples will be imported in the USA as per the CDC Permit to Import/Transfer Etiological Agents or Vectors of Human Disease.

### 12.2.5 Preparation of data for transfer to LSHTM

Two separate data entry clerks at PNLCC Niger will double enter the cost-effectiveness data using the "Access" program. The double-entered data will be verified, and any differences in data entry will be checked against the paper forms. The data manager will send the draft dataset to the Proctor study Coordinator for verification. After reviewing the dataset with the Proctor Database Manager, the Proctor study coordinator will transfer the final dataset to the representative at LSHTM.

### 12.2.6 Data Editing at the Coordinating Center

Through range checks, the data entry software ensures to a large extent that there are no inconsistencies or invalid collection data. Data will also be checked by the Database Manager for consistency and errors.

Specific software has been developed to verify consistency. The software will create an error file with relevant data such as the form identification, field names and data. The database manager will then consults the forms, resolve the inconsistency and enter the correct data. The corrected consistency error will then be electronically merged with the full database to give the corrected file.

### 12.2.7 Data management process

All completed Census Forms, Master Lists, Ocular Examination Forms, and Treatment Forms will be given to the Data Manager at the end of each workday and stored in a safe, secure place for transport to Niamey. The Data Manager will check the forms for completeness and accuracy.

- Master List and Census Forms: Forms have patient information/names and will be organized by the PNLCC Data Manager and stay in Niger at the PNLCC office, stored in secure, locked area.
- Ocular Examination forms: Should be immediately photocopied, with one copy stored with PNLCC and the other transported to the Proctor Foundation for double data entry under supervision of Proctor’s Data Management Specialist.
- Treatment forms: Treatment coverage calculated and recorded, forms organized and stored in secure place at the PNLCC office in Niamey.

### 12.2.8 Periodic reports

Study Coordinators and Data Managers will deliver periodic reports updating partners on each collection and treatment visit. Reports will be deliverable within 60 days of completion of the visit.

### 12.2.9 PCR result reports

All PCR results will be double-entered at Proctor within one week of obtaining results. Any inconsistencies will be resolved by the appropriate study coordinator (PNLCC or Proctor), and if necessary, by consultation with the appropriate examiner or lab personnel. Patient names will not be made available to UCSF personnel; individuals will be identified by ID number only. All data will be backed up upon entry, and forms will be kept in locked cabinets through the conclusion of the study.

### 12.2.10 Data Storage

At the data-coordinating center, the operator takes a backup copy of the day’s entry onto the data server and a CD. The data in the main computer will be backed-up daily and there will be at least two sets of backup disks at any particular time. All the backup CDs will be kept in a place different from the computer center.

## 12.3 Tanzania

### 12.3.1 Overview

Training manuals and baseline household census forms will be generated prior to the study commencement. Follow-up visit forms will be computer generated prior to the visit with the house/subject locator information already included on the forms. These forms will be produce based on information in the master files kept at the Kongwa central office. In addition to the forms, the computer program will print labels specific to each visit with the subject’s name, identification number, and visit number. The labels will be used to link laboratory specimens, and photographs to the participants, as well as to keep the inventory of the samples and of the photographs taken.

###

### 12.3.2 Daily Data Management Activities in Field

Each day when the team returns from the field, they will bring two types of folders with them. Each folder type must receive special attention. The first folder will contain the completed visit forms for participants who were seen that day. The second folder will contain the forms for all participants whose visit is still pending; this folder should be brought again to the field on the next field day.

Once all forms have been accounted for, each form should be reviewed for completeness following the steps outlined below.

1. Review each form for legibility and completeness of items entered. If any item has not been completed, place the form in the “For joint review” folder. Forms in the joint review folder should be reviewed at the end of the day with the person who completed the form.
2. Once all forms have been reviewed, take the stack of forms with questions to the person(s) who completed the form(s) and determine why the item(s) was/were not completed. If possible, complete the item with the assistance of the person originally completing the form. If the item is blank and the person who completed the form cannot return to collect any missing information to complete the item both the study coordinator and the person who originally completed the form should place their initials next to the blank item and place an “M” in the space where the data should have gone.
3. Return all forms to the appropriate folders for data entry.

### 12.3.3 Data Entry

A customized data entry system is being developed in Microsoft Access 2003 by programmers at the Dana Center at Johns Hopkins to facilitate data entry and management. The Project Director will install the system on available computers in the Kongwa office and will fully train the data entry clerks, and the project manager on how to use the system. Uses of the data system will include:

1. Electronic inventory of forms
2. Double data of key fields entry
3. Data editing
4. Archived history of editing changes
5. Generation of reports on performance and data quality
6. Production of data collection aids (follow-up schedules, reminders of upcoming and overdue follow-up contacts)
7. System backups
8. Generation of Forms

The management of the data will occur at Kongwa and Baltimore. The staff at Kongwa will have responsibility for accounting for the completeness and cleanliness for all data entered and sent to Baltimore. The Dana Center Data Coordinating Center will have responsibility for periodical reports on forms/data that are out of range, or missing and not accounted for, creation of the Master Data File, quality assurance reports for the field, and the performance of staff

Data entry will be performed using Microsoft Access 2003. The data system is designed such that each type of form is stored as a separate table. Variable names within each table are unique, with the exception of the participant-specific identifier that is used to link tables together. The initial screen prompts the data entry person to select the form type that is going to be entered. Next it prompts the clerk to enter a study id number. The structure of the program allows the information that is constant across forms (i.e. basic demographics) to be retrieved from the master table to each form within that participant’s visit.

The program contains a series of data checks within each form. In particular, the system will check to make sure that entries are within the allowed range, specific to each item entered. The data entry system also contains a series of checks for internal consistency (i.e. skip patterns) within each table, and does not allow duplicate entries. Finally, reports of missing forms for a particular visit are issued at the end of data entry day. Key fields in all forms will be entered twice, at the end of a data entry for the form, the double entries are compared. If all fields are identical, the entry is recorded into the database. Otherwise, discrepancies have to be resolved before the record is saved.

At time intervals during the day, and at the end of the data entry session, the computer prompts the user to do a backup save. The database is copied as a text files to another subdirectory in the hard drive, and to an external drive.

At the end of the day, the access database will be copied to an external drive. A different folder within the external drive is assigned for each day of the week, so that at all times we will have backup data to the end of the day for the last five days. Each week the data will be transferred to the Baltimore office, under the supervision of the project manager.

General guidelines for data entry

1. Each clerk will be assigned one community at a time for data entry. The data entry clerk should only enter forms from that community on the computer assigned to him/her.
2. Always, enter census (census-update data) forms before any other forms (ocular forms, logbooks). Upon completion of data entry of a specific village visit, the data entry clerk should file the village-visit folder in the “completed data entry” file cabinet.
3. Each form should be initialed and dated by the data entry clerk at the time of entry. If there are questions regarding a specific form, the form should be placed in a designated folder for consultation with the data manager later that same day.
4. Enter all items on the forms exactly as they appear. Do not attempt to determine what the person completing the form meant; ask the project manager if you have questions.

### 12.3.4 Preparation of Data for Transfer to JHU Data Coordinating Center

The project manager, will supervise data entry. He/she will be responsible for integrating the data bases entered into the individual computers into a single data base, the master file, containing the data from all communities. Each week, he/she will email the data files to the Dana Center where the study statistician will perform additional data checks.

To assure confidentiality, all data will be sent encrypted and pass-word protected. Before sending the data file(s) via email, they must first be encrypted. WinZip will be used to compress and encrypt the file(s). A password will be sent to the Kongwa office to use in the encryption process. WinZip can be downloaded from the web at www.winzip.com, or it can be provided to the data centers by Johns Hopkins if web access is not possible.

To zip and encrypt a file:

1. Select the file or files that you want to send

- For a single file, right click on the file
- For multiple files, use the mouse to lasso the files, then right click on the files while they are highlighted

1. Choose **Add to zip file...**

- Your options may be slightly different, depending on the installation of the WinZip software.

1. Under Options, choose **Encrypt added files**
2. Push the **Add** button
3. Enter the password provided to you by Johns Hopkins
4. Reenter the password
5. Under Encryption Method, choose **128-Bit AES encryption**
6. Press the **OK** button
7. You may now exit the WinZip utility and email the resulting compressed file (ending in .zip)

12.3.5 Data Editing.

At the Dana Center the study data manager/statistician will perform additional data checks. Discrepancies will be sent to the project manager for reconciliation by reviewing the original data forms. Corrections will be noted on the printout and returned to the Dana Center. The project manager will make edits to the master file. He/she will be the only person with permission to make edits to the master database. Records being edited, along with the date and time, will be automatically archived into an external database prior to implementing the changes. To create an audit trail, a report containing the operator, the type of transaction, the date, the old values, and new values will be generated.

12.3.5.1 Specimen transfer data procedures.

During field work at collection time, laboratory samples will be placed in collection boxes that have been numbered in advance. The box number as well as the label identifying the specimen will be recorded in both the ocular specimen shipping list and the corresponding ocular exam form. At the end of the field day the specimen boxes will be storage in a freezer that will be kept at -80°C. Once a visit has been completed, specimens for that visit will be packet according to the procedures described in section 6.5 and send to the Baltimore laboratory for processing. In accordance with the Roche COBAS AMPLICOR™ CT/NG protocol, swab samples taken in the field will be transported on ice in a closed, insulated container until arrival in Baltimore, where they will be stored at -80°C for later analysis. Samples will be imported in the USA as per the CDC Permit to Import/Transfer Etiological Agents or Vectors of Human Disease.

At the same time, a copy of the shipping log for each box will be sent. The laboratory will be responsible logging in samples and report to the Dana Center any discrepancies between the shipping log and the actual shipment. The Dana Center data management team will work with the project manager in Kongwa to resolve all inconsistencies.

### 12.3.6 Generation of Follow-up Lists

At each visit and for each community the study coordinator will be responsible for supplying the field team with a census book to update the census with new household members, permanent moves out of the village and deaths that occurred in the previous six months. The census book will be generated by clicking the appropriate bottom under the reports menu of the access data base.

Once the data entry for the census update has been completed, the computer will select at random the sentinel children to be examined, and the ocular forms and corresponding labels for the selected children will be printed. If the visit includes annual treatment, treatment forms for each household will be also generated.

### 12.3.7 Periodic Reports

Reports will be issued for each visit with summary information on key variables. In order to explore problems with standardization, the reports will be stratified by community, and by interviewer/examiner. If any irregularities are detected, investigators will be informed and re-standardization of pertinent procedures will take place.

Participant tracking reports will be generated for each visit. These reports will be given to the project manager, who will determine whether the visits have been conducted. If the visits were not conducted, he/she will work with the field team so that a follow-up visit can be scheduled. If the visit was conducted, but no data have yet been entered into the data system, he will make sure that the data for that visit is entered.

Periodic reports for quality assurance will also be prepared. These include the following:

Trachoma grading: The Coordinating Center will receive the grades from Dr Bailey on the ocular photographs for each grader at each visit. These will be compared to the clinical grades given to the same children. Agreement will be determined, using the kappa statistic, for TF and TI. A report is generated on the agreement to the Study site investigator and to the Executive Committee. If agreement is below kappa 0.6, then the Study Site investigator will responsible for carrying out re-standardization exercises as described in Chapter 17.

Field Laboratory specimen reports: the Coordinating Center is responsible for receiving the data on the positivity of the air field specimens, and generating a report on the rate of positivity by visit and by lab technician. A report is generated to the Study Site Investigator and to the Executive Committee. If field contamination is greater than 1%, then remedial actions in the field are required to bring the field operations back to standard, as discussed in Chapter 17.

### 12.3.8 Data Storage

At the office in Kongwa, data will be stored in locked file cabinets. Electronic databases in Kongwa will be backed up to an external drive on a daily basis and periodical backups will be stored off-site. At the Dana Center, for the analysis phase, the data will be transferred into a Sun station UNIX base system, where SAS permanent data files will be created. Three generations of backup files are kept at all times for this computer. One copy is housed off-site. In the central computer, tape backups are performed daily. Four tapes are used during the week (labeled Monday-Thursday). There are four Friday tapes (Friday1 to Friday 4) for each Friday of the month. At the end of every week (Friday), backups are done on the weekly tapes. There are twelve monthly tapes (January to December). At the end of each month, the backup is done on the monthly tape. This procedure ensures the possibility of retrieval of old versions of files up to one year old.

To maintain data and study documentation, codebooks with question-by-question specification of all questionnaire responses will be created and updated periodically. Furthermore, a complete description of all data files will be kept. This description will include the file creation date, the number of records, the record layout, the field names and description and a list of allowable codes for each field.

12.3.9 Preparation of data for transfer to LSHTM, UCSF

Once the data for a single visit for all the communities has been entered and edited, SAS permanent data sets will be created. These data sets will be used for all the statistical analysis and the generation of official reports. Customized programs will be used to extract the information required for the analysis to be carried out both in San Francisco and London. The de-identified data and corresponding documentation will be sent electronically using the data transfer protocol described in the previous section. Persons are only identified using a study number.

## 12.4 The Gambia

### 12.4.1 Study Forms Preparation

#### 12.4.1.1 Census Forms

At baseline, each census taker is given a set of forms for a census enumeration area (CEA), and a list of household numbers unique to them, developed by prior listing of household heads. Census enumeration areas have unique ‘geocodes’ in the 2003 Gambian census which are five digit numbers. The household numbers may begin with a unique letter, then sequential numbers. At subsequent census, a master Census book is created with the most recent version of the census for that CEA, which is used to update the census with new arrivals and departures.

#### 12.4.1.2 Master Survey List

The Master Survey List is a copy of the original census document. In the 3 ‘longitudinal’ CEAs all children 0-9 are chosen, and in the 9 ‘sample CEAs 100 children aged 0-5 are randomly selected, together with 10 reserves. The registration form then contains the full spelling of each individual’s name, as well as their age, gender, and household number. A new randomized Master Survey List is generated for each CEA at each collection visit and is used for both mobilization (the day before collection is scheduled) and registration.

#### 12.4.1.3 Ocular Examination Form

The Examination Form is printed for each of the children who will be part of the sample survey in each CEA. Labels are also pre-printed for placing on sample tubes, photo log books, and specimen shipping log books.

#### 12.4.1.4 Blank census update forms

Blank forms are brought each day to the field during surveys or mass treatment in case they are needed to update information on sample children or residents.

### 12.4.2 Daily Data Management Activities in Field

All completed Master Survey Lists, Census update forms, Ocular Examination Forms and, depending on the activity, mass Treatment or Census books will be given to the Study Coordinator at the end of each workday and stored in a safe, secure place for transport to Fajara The Study Coordinator will check the forms for completeness and accuracy.

### 12.4.3 Data Entry

The treatment data collected in the field will be entered into a database at The Sheikh Zayed Regional Eye Care Centre by National Eye care Program staff. A double entry on key fields, using the uniform Access data entry package created for each site, will be done to minimize data entry errors. The study coordinator will do the final editing. The collection fieldwork data will be entered into a database by The Gambia’s Database Manager, who will also oversee the work of the staff member helping to double-enter the data. The Gambia Database Manager will also enter all PCR results into the database.

### 12.4.4 Preparation of Data for Transfer to LSHTM

#### 12.4.4.1 Data Management

All treatment and collection data will be entered within four working weeks of collection. Data will be entered and inconsistencies will be resolved by the respective study coordinator or, if necessary, by consultation with the appropriate data collector. Access to any patient information will be protected by a password, or locked in a secure storage room. All data will be backed up upon entry, and forms will be kept until the conclusion of the study.

#### 12.4.4.2 Reconciliation of Data

Before data entry, the forms will be reviewed and cross-checked for consistency and completeness. If the forms are not filled out completely, the Study Coordinator will contact the person responsible for completing the form to provide missing data or clarify any inconsistent data. The Study Coordinator is the only person who is authorized to add missing data or make any changes to the study forms. All changes will be made in a different color ink and initialed and dated.

#### 12.4.4.3 Specimen Transfer Procedures

In accordance with the Roche COBAS AMPLICOR™ CT/NG protocol, swab samples taken in the field will be transported on ice in a closed, insulated container to Fajara, where they will be stored at -80°C for analysis on site. Where sample aliquots need to be sent outside the Gambia for QC or further analysis they will be imported into the USA as per the CDC Permit to Import/Transfer Etiological Agents or Vectors of Human Disease, and into the UK as per COSSH (Control of substances hazardous to health) guidelines. Locally stipulated regulations for the transfer of samples from MRC The Gambia will be fully met.

#### 12.4.4.4 Preparation of Data for Transfer to UCSF

Once the data for a single visit for all the communities has been entered and edited, SAS permanent data sets will be created. These data sets will be used for all the statistical analysis and the generation of official reports. Customized programs will be used to extract the information required for the analysis to be carried out both in San Francisco and London. The de-identified data and corresponding documentation will be sent electronically using the data transfer protocol described in the previous section. Persons are only identified using a study number.

# **Chapter 13: Quality Assurance Policies and Procedures**

Chapter

13

______________________________________________________________

## 13.1 Overview

Several policies and procedures are built into this project in each country in order to provide assurances of quality in the data collection, processing, and management, as well as assuring proper treatment of all participants. General QA procedures include:

- Intensive training sessions: All individuals involved in the study will undergo training and certification for all study procedures for which they are responsible. Training and certification procedures are described in chapter 11.
- Development of standardized forms and procedures for completion of forms. This includes procedures for recovery of missing data and changing responses, if necessary, as described below.
- Monthly data editing, report generation, and reconciliation, with feedback to the clinical sites in The Gambia, Niger, and Tanzania, and the laboratories by each Data Coordinating Center. These procedures are described extensively in each Chapter, and in Chapter 12. The process of providing periodic feedback on missing data or incomplete or out of range data is critical to timely recovery, identification of problems, and remedial actions to ensure that problems are solved.
- Monitoring of quality of trachoma grading and laboratory procedures used in the field, and in the laboratory. The Data Coordinating Centers will review the adherence to randomization procedures, treatment administered, timely follow-up of villages on schedule, agreement among graders for trachoma assessment in collaboration with Dr Bailey, and laboratory evidence of contamination of specimen collection or inadequate specimens. If the review uncovers a problem, the CC will notify the Study Site Principal Investigator, as well as issue reports to the Executive Committee. The clinical sites will immediately institute retraining procedures, including suspending a staff member from performance of the task until adequate performance can be demonstrated.
- Within each clinical site, the project directors have planned bi-weekly meetings of the census, survey, treatment, and the Data Entry Teams, to review reports and study progress, and address problems identified by the Data Coordinating Center.
- Masking of survey team as much as possible, and certainly the laboratory personnel, to treatment randomization. This procedure avoids bias in detection of infection, and determination of outcome.
- Active oversight by the Project Director in the field at the commencement of each study visit. The project directors will ensure the next phase of each part of the project proceeds smoothly, the data flow is steady, and the feedback for the new phase is in place.
- Site Monitoring with 6 monthly site visits by the Study Site Principal Investigator. These will focus on adherence to protocols, review of team meetings, and resolution of any issues.
- Creation of a paper or electronic trail to any data modifications and limiting access to the main database. Extensive back up procedures that protect the quality of the database. These procedures help ensure the integrity of the database.
- The Executive Committee will meet every six months to monitor, across sites, study progress, address any issues arising from data quality monitoring, review safety reports and review consent procedures.

## 13.2 General Guidelines for Form Completion

A significant component of quality assurance for data collection is the proper completion of forms. Detailed instructions for the completion of each form are provided in chapter 10, so this section is intended to provide guidelines for the completion of all forms. The following rules apply when completing the forms:

• Complete forms in “real time”. Do not fill in the forms at a later time, or more convenient time. Later completion has a high potential for recall bias, missing data, or wrong data. The form must be filled in while the child/family member is present. The most difficult form to accomplish this will be the mass treatment form, which may be completed over a period of days for the village. However, it is essential that all persons be accounted for in terms of treatment. In the event of missing data, the staff must NOT try to recall the missing treatment but re-question the family to capture the data. If this cannot be done, it is better to code the data as missing (see below).

• Use only black ink for initial forms completion

• Use only red ink for alterations/edits to original entries on forms, which must be initialed by the person making the change.

• Check to make sure that ID labels/data and **study** identifiers are on every page of every form. Forms that do not have this information are to be referred back to the study site project directors for clarification and review.

• Print all written responses

• Do not change units or re-phrase questions.

• Enter data in the units and number of digits prescribed on form. Do not record fractions. Use a decimal only if it is written on the form. Be sure to include a value behind a decimal point, if applicable.

• Right justify all numbers, entering leading and following zeroes where applicable.

- Left justify all letter codes, leave remaining spaces blank.
- Write pertinent comments in the margins or on the last page of the form.
- Review all responses for completeness; skip patterns and accuracy before signing off on a form.

## 13.3 Missing Data on Study Forms

Missing forms or specimens are discussed in Chapter 12. In summary, procedures conclude with the person responsible signing off on missing items or forms or vials that all attempts to locate have been undertaken and the item is missing. This form is transmitted to the Coordinating Centers in order to update the Master File.

If some data within forms are missing and cannot be obtained when the form is reviewed, then an appropriate code is inserted in the empty data field to indicate missing or not applicable.

## 13.4 Changing Responses on Study Forms

In general, responses should not be changed unless there was a transcription error or the discovery of incorrect data by one staff on a form prepared by another. In these cases, the following guidelines should be adhered to:

Preserve the audit trail, so changes can be traced from original response to the changed response. Often, this means nothing more than making corrections on the original form. In the case of responding to questions raised by the data entry clerks, be certain that the missing data/specimen forms are properly filled out and accounting made to site project directors.

Do not obliterate, erase or white-out incorrect response. To correct a response: draw 1 or 2 lines through incorrect response, write correct response next to or about it in red ink, put initials and date in the margin by the correction.

## 13.5 Oversight

The Project Directors at each site will be responsible for overseeing the initiation of all phases of data collection. Thus, together with the study site principal investigators, the project directors will be present at the start of each new phase of data collection, specifically the census, surveys, mass treatment, and new rounds of each. The Project Director will be responsible for ensuring that each type of visit runs smoothly, and will remain onsite until all individuals involved in data collection for that visit type are comfortable with the data collection procedures. They will also be certain that the Data Entry team follows the procedures for each type of data entry, is operating smoothly, and responsibly storing the forms.

Bi-weekly staff meetings of project staff during census, survey, and will be held to review progress in enrollment/follow-up, review outstanding problems with forms or data entry, and reconcile reports from the coordinating center. The staff can report field problems, issues in dealing with the villages, and other concerns that might arise. All staff can attend, including drivers.

## 13.6 Adherence to Procedures

The Data Coordinating Center for each clinical site will monitor adherence to the procedures for randomization, mass treatment administered, census and follow-up surveys, adherence to standards for trachoma grading and laboratory evidence of contamination of specimen collection or inadequate specimens. Mechanisms are in place to ensure that villages are randomized to each treatment group in the absence of bias by study staff; and the treatment assignment is not known to the laboratory personnel.

## 13.7 Quality Assurance for Field Trachoma Grading

The images taken will be organized into folders for each examiner by visit. The folders will be sent to Dr Robin Bailey for review and grading, as he is the standard to which all the field graders have been trained. He will grade each set of 50 digital images for quality of the image, using 2.5 magnification. The first observation is for grade-ability of the image. If more than 20% of the images are ungradeable for any one photographer, the clinical site will be notified, certification of photographer will be suspended and the photographer will have to be re-certified.

Dr Bailey will grade each gradable photograph with a clinical grade for trachoma, using the WHO simplified grading scheme. The grades will be transmitted to each study site data coordinating center, which will compare the grades with the grades assigned to the eyes in the field. Agreement will be assessed, and if agreement falls below 0.6 in any month, the reasons for disagreement will be discussed, and the set of photographs re-circulated to bring agreement to acceptable levels. If necessary, the survey will be interrupted while the study site lead investigator and the field grader re-grade photographs and undertake field work until agreement is re-established. Some of the disagreement could be due to differences between grading photographs and grading live eyes in the field, although we have shown very high concordance.

The quality assurance of grading in the field is a required report from each center that will be presented at six months to the executive committee for review and reported to the DSMC during their annual review.

## 13.8 Quality Assurance for Specimens

Once per year, a set of five positive and five negative specimens from each laboratory will be re-aliquoted and half the sample will be sent to Dr Lietman’s laboratory, masked as to the findings from the original lab. Dr Lietman’s lab will re-run the results, as will the lab of origin for confirmation of results, thus providing assurances of similar procedures across laboratories. There must be at least 80% concordance of results between the laboratory and Dr Lietman’s lab.

The following steps will be followed:

- Dr Lietman and the lab of origin will send their results to the study site CC.
- The CC will send a report of the concordance, with ID numbers of non-concordant results, to the lab of origin and to Dr Lietman.
- If concordance is at least 80%, the CC will issue a one year quality assurance certificate to the lab and to Dr Lietman for his records, and to the Executive committee.
  - If concordance is less, the CC will issue a provisional notice to the lab chief and Dr Lietman, who must work together to resolve the difference, including re-sending specimens until concordance is reached. Once concordance is reached, the quality assurance certificate is again issued to the lab and to Dr Lietman for his records, and to the Executive Committee. Note that some of the discrepancy may be due to issues with shipping, resulting in loss of positivity of specimens. To the extent possible the re-aliquoted specimens in the laboratory will be stored under conditions that mimic the shipping and re-storage so that conditions of the specimens are similar.

The Executive Committee will review yearly the quality assurance certificates for the laboratories. Data on continued quality assurance will be presented to the DSMC.

There is also the possibility of field contamination, which is checked by using the “blue air” specimens. Each laboratory will process these controls masked as to their status as controls. The CC for each site is responsible for reporting the positivity of the negative field controls to the study site investigators. If the rate is above 1%, then the investigators must take remedial action to correct field procedures to remove contamination. Data on field contamination results will be presented to the DSMC for each site by the CC.

# **Chapter 14: Adverse Events**

Chapter

14

________________________________________

Azithromycin is being given to these communities under the auspices of the National Trachoma Control programs in each country. They have determined, for programmatic reasons, that widespread coverage with azithromycin is an acceptable community risk for the benefit of eliminating blinding trachoma. Approximately 100 million doses of oral azithromycin have now been distributed for trachoma, and reports of serious side effects are essentially non-existent. This may be due in part to minimal surveillance. In fact, where carefully monitored, there were actually fewer GI side effects after taking azithromycin compared to topical tetracycline, perhaps because azithromycin treats some cases of diarrhea. Azithromycin is generally well-tolerated. The most common side effects of azithromycin and erythromycin are diarrhea or loose stools, nausea, abdominal pain, and vomiting, each of which may occur in fewer than one in twenty persons who receive azithromycin. Rarer side effects include abnormal liver function tests, allergic reactions, and nervousness.

All community residents will be advised to alert the village health worker if they experience, within two weeks of mass treatment, a serious adverse event, defined as diarrhea, nausea and vomiting for more than two days, hospitalization for any cause, or death in a family member. All adverse events will be recorded on a copy of the census list left with the village health worker by the mass treatment teams. These events will be reviewed at the next six month survey, and entered into the adverse event data base for reporting to the Data and Safety Monitoring Committee. If, for any reason, the village resident needs further care, they will be referred to the nearest health center for examination and treatment, and the most appropriate action will be taken to provide immediate care, in accordance with the policies in each country.

# **Chapter 15: Ancillary Studies**

Chapter

15

_______________________________________________________________

## 15.1 Risk of Re-emergence: Pediatric Longitudinal Study

We need a sample which can be followed longitudinally over time to determine how disease or infection re-emerges, and the value of multiple exposures to treatment. For this, a longitudinal sample of all children is needed. The sample for the trial is a simple cross sectional and thus the sample members may change at each round (as they age, or new residents move in, or as a consequence of sampling) this sample cannot address the critical questions of infection, trachoma, and re-emergence within children by number of years of mass treatment. For this, we propose a longitudinal cohort of children in a wider age range, for whom we can follow age and cohort effects. The second sample will consist of a longitudinal cohort of children ages nine and younger who will consist of all children in communities which are a sub-set of the randomly selected communities. For the longitudinal sample, we will randomly select up to three of the communities in each arm in which we will follow all children age 0 to 9 years at baseline. This sample will be a maximum of 750 children per arm, and a minimum of 300 children. Each year we will re-census the population of the communities to enroll any new children ages less than nine years. In this way, we can monitor the effect of treatment on successive age cohorts who have experienced variable numbers of rounds, and coverage, of treatment. They will also be followed up at each visit scheduled for the random sample, and exactly the same procedures for trachoma grading and ocular specimens will be used.

For example, within each arm of the trial for Tanzania, we will have randomized 16 communities. Within each arm, at least one community will have all children ages less than 9 years followed longitudinally. These children will also serve as the random sample at baseline of children ages 0-5 years.

## 15.2 Modeling Trajectory of Disease Elimination: Public Health Planning Study

The optimal frequency, coverage, or duration of mass azithromycin distributions is unknown. While clinical trials are the gold standard to assess efficacy, they cannot explore all potential strategies for the different settings of interest. Mathematical models of infection transmission are a natural method to generalize the results of our specific trials. Here, we will use data from the proposal’s clinical objectives to estimate region-specific transmission parameters for the mathematical model of trachoma. We will then use the model to determine the optimal frequency, coverage, and duration of treatment for different levels of endemicity.

## Ancillary Benefits of Mass Treatment with Azithromycin

As part of the larger PRET study, we will nest a study evaluating the added value of mass treatment on these other common morbidities. This ancillary study is not a randomized trial, because we could not randomize villages that met trachoma criteria not to receive azithromycin. However, in terms of other diseases, we do not expect differences in baseline prevalence. We intend to analyze the data at the village level to start.

The study would be conducted in Kongwa, Tanzania, in four communities already censused and slated to receive mass treatment with a single dose of azithromycin (Intervention communities). Four control communities would also be selected that did not have trachoma rates high enough to warrant mass treatment with azithromycin (mass drug administration or MDA). We plan to randomly select samples of children and adults in each of the eight villages for a series of cross sectional surveys and interim, bi-weekly, surveillance for six months.

## Cost Effectiveness Study

The cost effectiveness analysis will be based on community cost estimates that we will calculate using the cost collection instruments described below. For each community, we will collect cost information regarding: all medical and non-medical personnel involved in the study and all consumables used. We will also include laboratory costs attributable to screening/treating each community. The totality of the equipment costs - such as vehicles and field equipment costs – and laboratory costs will be recorded aggregately and then allocated to each community according to the community population. Further information on this ancillary study can be found in Chapter 9.

## Laboratory Screening Study

The laboratory screening study evaluates the utility of a trachoma laboratory screening for identifying “graduated” communities in Niger, Tanzania, and The Gambia.

# **Chapter 16: Data Analyses Plan**

Chapter

16

___________________________________________________

## 16.1. Primary Outcome Analyses

The data analyses will address several key questions about mass treatment. The analysis will be performed on an “intention-to-treat” basis, according to the community’s original treatment assignment. In addition actual treatment compliance data for each community will be collected and used in the secondary analysis, which will allow us to directly model those communities where mass treatment was stopped. The main result of this study consists of two comparisons: one is between trachoma/infection prevalence in communities randomized to the two target coverage arms; the second between trachoma/infection prevalence in communities by either treatment frequency (yearly versus 6 monthly) or yearly versus according to the stopping rules for mass treatment. Initially, we will describe the comparability of the four intervention arms by comparing average community size, our key environmental variables, and baseline trachoma/infection .We will evaluate differences overall and among countries, as these differences can be incorporated as explanatory variables in the models.

To determine the changes in prevalence overtime we will conduct cross-sectional analyses of data for each of the follow-up visits: 6, 12, 18, 24, 30, and 36 months as well as longitudinal analysis incorporating the effect of time. The analysis will be carried out at two levels: first, using individual communities as the unit of analysis and second using the sentinel children as the unit of analysis and accounting by the clustering effect of children living in the same community.

These two approaches are complementary.

### 16.1.1 Community-level Analysis

Cross-sectionally, trachoma/infection prevalence rates will be presented for each community and each time period stratified by randomization arm. We will test for differences in the mean prevalence rates between groups using a non-parametric approach: the Wilcoxon rank sum test when comparing two groups or its extension, the Kruskal-Wallis test when comparisons involve more than two groups.

To evaluate changes in prevalence over time, we will create mixed models with random effects using the logit of the prevalence of trachoma/infection as the outcome and allowing each community to have its own slope and intercept. As there is reason to believe that the two interventions may interact with each other, the possibility of an interaction effect should be explored. The factorial design is optimally posed to test for such interaction. The models will take the following form:

$$Log\left(\frac{P_{it}}{1-P_{it}}\right)=(\alpha_{0}+a_{i})+(\beta_{0}+b_{i})t+\beta_{1}x_{1i}t+\beta_{2}x_{2i}t+\beta_{12}x_{1i}x_{2i}t+\gamma z_{pi}+e_{it}$$

Where P*_it_* is the prevalence of trachoma/infection for community *i* at time t, *x_1i_* and *x_2i_* are indicator variables for the treatment assignment, high compliance with antibiotic treatment or treatment based on the stopping rule respectively. α_0_ is the expected logit of the prevalence of trachoma/infection at baseline, which due to randomization is expected to be the same for the intervention groups. The deviations *a*_i_ of the prevalence of each of the communities from α_0_ is handled in the model as a random effect. β_0_ is the fixed effect quantifying the expected decline of the prevalence of trachoma/infection with time on the study in the lower compliance without stopping rule group and b_i_ is the corresponding random effect allowing each community to vary from the overall rate of decline. β_1_, β_2_ and β_3_ are the parameters to test the null hypotheses of interest (i.e., the effect of interventions). All three parameters have the interpretation of accelerating the rate of decline of the prevalence of trachoma/infection. Specifically, β_1_ is the intervention effect for communities randomized to high antibiotic compliance only, β_2_ is the intervention effect in communities randomized to treatment based on the stopping rule, and (β_1_ + β_2_ + β_12_) for communities randomized to both high compliance and antibiotic treatment based on the stopping rule. Since at baseline there has not been any intervention and the groups are randomized, we do not expect any differences at baseline and thus the model should not include a main effect of the intervention groups. Were there to be a difference due to imperfect randomization, we would include a main effect. Furthermore, in the case that the interventions do not continuously depart from each other, we will categorize time instead of using it as a continuous variable. This model can be further extended to include additional variables (*z_pi_*) to explore the effect (γ) of environmental factors such as presence of water source, percentage of households with latrines, etc, on prevalence.

### 16.1.2 Child-level Analysis

Using the sentinel children, we will construct for each period multivariate models with presence of active trachoma/infection as the outcome. Logistic models of the following form will be built to estimate the effect of the two treatments and their interaction:

$$Log\frac{\Pr(Y_{i}=1)}{\Pr(Y_{i}=0)}=\beta_{0}+\beta_{1}x_{1i}+\beta_{2}x_{2i}+\beta_{12}x_{1i}x_{2i}+\gamma z_{pi}$$

In this model, *Y_i_* is an indicator variable for presence of trachoma/infection, *x_1i_* and *x_2i_* are indicator variables for the treatment assignment, high compliance with antibiotic treatment and treatment based on the stopping rule, respectively. β_1_ is the intervention effect for children living in communities randomized to high antibiotic compliance only, β_2_ is the intervention effect for children living in communities randomized to treatment based on the stopping rule, and (β_1_ + β_2_ + β_12_) for those living in communities randomized to both high compliance and antibiotic treatment based on the stopping rule. The models will include additional covariates (*z_pi_*) to adjust for initial/previous period trachoma/infection status at community level and other factors thought to be related to the outcome that in spite of randomization were dissimilar at baseline. Because the outcomes for children living in the same community are likely to be correlated, all standard errors will be corrected to account for that correlation using the Generalized Estimation Equation approach. Since GEE approaches work best when there are a large number of fairly small clusters, we will consider an alternative approach. Using communities as the cluster we will also implement a random effects model allowing the intercept *β_0_* to be (*β_0_ + a_i_*) where *a_i_*~ N(*0,σ^2^*) corresponding to the random variation of the *i^th^* child in a given community. The models are easily implemented using PROC NLMIXED in SAS. We will compare the results of these two approaches to examine the consistency of the inferences.

We plan to evaluate the correlation of clinical trachoma with laboratory evidence of infection in these communities at each survey time point, within treatment arms. It is recognized that in an undisturbed state, there will be more active trachoma than infection, as well as some sub clinical cases of infection. However, following mass treatment there is a pronounced disassociation, as infection declines dramatically with more modest declines in disease rates. Indeed, that is the premise of our contention that a test of infection that indicates low levels may be a better indicator of when to discontinue mass treatment than a clinical indicator. However, over time, clinical trachoma declines as well, although the trajectory is not well described.

## 16.2 Additional Analyses

### 16.2.1 Cost Effectiveness Analyses

The cost effectiveness analyses to be done are twofold: One analysis will compare the cost-effectiveness different frequencies of screening – i.e. six months versus every twelve; and yearly versus having a stopping rule. The second analysis will compare the cost-effectiveness analysis of the intervention when treatment is triggered based on an 80% to 89% coverage versus triggering it based on a 90% coverage.

For both types of analysis, the measure of effectiveness will be the *proportion of communities fulfilling the “stopping” criteria after the 3 years*. And, for both types of analysis, we will use the approach the incremental cost effectiveness analysis ratio (ICER) approach by which the ICER is calculated as:

$$ICER_{AversusB}=\frac{C_{A}-C_{B}}{E_{A}-E_{B}}$$

where *C­_i_* for *i=A, B* is the cost of following strategy *i* (accumulated costs of strategy *i,* which are discounted at the 5% discount rate when aggregated in a multiyear period); and *E_i_* is the value of that the measure of effectiveness takes following strategy *i*.

#### 16.2.1.1 Frequency: 1. six monthly versus yearly; 2. Yearly versus stopping rule

To compare the Trachoma control intervention with a 6 months frequency to that of 12 months frequency (in Niger) we will first of all calculate the average cost per community (adding those recurrent costs to those prorated capital costs and the laboratory costs) in the 6 months arm. We will do the same for those communities in the 12 months arm. Then, we will apply an incremental cost effectiveness analysis approach and we will calculate the ratio resulting from dividing the differences in average cost of those two modalities of intervention by the difference in their average effectiveness.

To compare the yearly screening versus the stopping rule interventions in Tanzania and The Gambia, we will do similarly and calculate the average cost and average effectiveness of those communities in each arm. Then, we will calculate the incremental cost effectiveness ratio by dividing the cost differential by the effectiveness differential.

#### 16.2.1.2 Coverage: 80%-89% coverage versus 90%+ coverage

Equally, to compare the 80%-89% versus the 90%+ interventions, we will do similarly and calculate the average cost and average effectiveness of those communities in each arm. Then, we will calculate the incremental cost effectiveness ratio by dividing the cost differential by the effectiveness differential.

### 16.2.2 Model of Trachoma and Infection Elimination

We will simulate trachoma transmission using a stochastic (random process) model, where individuals are assumed to be repeatedly infected. We chose a stochastic modeling framework because chance effects are more important when we near elimination, and because experience with previous studies suggests considerable intercommunity variability which cannot be explained by other known indicators such as baseline prevalence, socioeconomic indicators, or antibiotic coverage. The model focuses on the core group of pre-school children, known to have the highest risk for and to carry the highest burden of infection. We assume that each child is either susceptible (not infected), or infectious, leading to a standard SIS model of transmission^8, 9^. We denote the number of infected children at any given time by *Y*, and the total number at risk by *N*. When a mass antibiotic administration occurs, a fraction *c* are covered with an antibiotic of efficacy *f*, and each infected individual has a probability *cf* of being cured (and susceptible again). Between mass treatments, we assume that susceptible individuals become infected at a rate (*β* *Y/N*) proportional to the prevalence fraction *Y/N* of infection in the population. Once infected, individuals recover due to natural processes (even in the absence of antibiotic) at a constant rate*γ*, and again become fully susceptible once. These assumptions yield a standard continuous-time Markov process (with constant rates) which models the number of infected individuals until the next mass antibiotic administration occurs.

To estimate the site-specific transmission parameters for the stochastic model from epidemiological data, we use previously described methods[[22]](#footnote-23),[[23]](#footnote-24),[[24]](#footnote-25). Briefly, we apply kernel-density estimators to simulation results to estimate a likelihood function from site-specific data collected before and after treatment. We maximize this likelihood, yielding both parameter estimates and their associated confidence intervals. Sensitivity and uncertainty analyses will be conducted on all parameters. Additional extensions to the model will include predicting the blinding sequelae of trachoma. All simulations and calculations will be conducted using *Mathematica* 6.0 (Champaign, IL).

This objective will proceed in several stages. In the first year of the project, we will construct the model given existing, pre-proposal data from each of the three study areas and from data available from other regions. Next, we will use the baseline and 6-month results of the antibiotic trial in this proposal to estimate site-specific parameters. We will validate the models using subsequent data from each of the sites in subsequent years. In years 4 and 5 of the proposal, we will use the models to estimate optimal treatment strategies, determining the most cost-effective coverage, frequency, and duration of treatment necessary to achieve elimination in 90% of communities.

Sensitivity analysis will be conducted based on varying the recovery rate parameter and repeating the simulations to ensure consistency (as we have done previously).

Additional extensions to the model will include within-community, household level heterogeneity. Transmission of infection may vary considerably even within pockets of a community, and these may be the same pockets that are most difficult to reach with antibiotic coverage. The longitudinal cohort studies in each of the three countries will provide the data necessary to assess the importance of heterogeneity and to adequately characterize it for inclusion in the model.

We can also use existing data to translate our conclusions about the prevalence of infection into what would be expected in terms of its consequences, clinical trachoma and trichiasis, the measures used by the WHO and trachoma programs.

### 16.2.3 Re-emergence: Longitudinal Study of Cohorts of Children

In each of the treatment arms there will be communities where all children aged 0-9 years will be followed overtime. To identify factors associated with presence active disease/infection in children, the following regression model will be used:

$$Log\frac{\Pr(Y_{it}=1)}{\Pr(Y_{it}=0)}=\alpha_{0}+\beta_{1}x_{1i}t+\beta_{2}x_{2i}t+\beta_{12}x_{1i}x_{2i}t+\beta_{3}t+\gamma z_{it}$$

Where Y_it_ is an indicator variable for presence of trachoma/infection for child i at time t, x1i, x2i,, β1, β2, β12 have interpretations similar to the ones depicted in the previous longitudinal community level models, β3 describes the effect of time for the x1i=0 and x2i=0 group, and γ the effect of personal risk factors. In this model the effects of interest are quantified by β1, β2, β12 and correspond to different rates of change due to treatments. To account for the within-person serial correlation, standard errors will be corrected using the Generalized Estimation Equation approach. However, in these models, we can account for number of rounds of mass treatment experienced by the child, as well as the community, and model.

These cohorts also permit us to determine risk factors for re-emergence of infection or trachoma in children who at a previous round were free of either one and thus at risk. We will determine the risk associated with re-emergence as a function of number of previous rounds of mass treatment, and investigate the magnitude of infection/disease re-emergent clustering by family or neighborhood.

### 16.2.4 Specific Analyses for the Niger site

The following analyses are planned for the Niger site (UCSF). All tests are two-sided with an alpha level of 0.05.

- - In the annual azithromycin arm, we propose to compare resistance levels in pneumococcus from nasopharyngeal swabs taken at baseline to resistance levels at 36 months, to determine whether or not resistance levels have risen. This will be conducted using the Wilcoxon signed rank test on the differences (thus accounting for pairing). In all uses of linear regression or linear models with prevalence as the outcome, we propose to use the square root or fourth root transformation to stabilize the variance.
  - In the biannual children arm, we propose the same analysis. Specifically, we propose to compare resistance levels in pneumococcus from nasopharyngeal swabs taken at baseline to resistance levels at 36 months, to determine whether or not resistance levels have risen. This will be conducted using the Wilcoxon signed rank test on the differences (thus accounting for pairing).
  - At 36 months, we propose to compare the resistance levels in pneumococcus from nasopharyngeal swabs between two arms: the annual arm and the children biannual arm. We wish to determine whether or not treating children biannually causes more resistance than treating everyone annually. We propose to use the Wilcoxon rank sum test (there is no pairing).
  - We propose to compare the chlamydia infection levels (from PCR taken from conjunctival swabs) in adults at baseline to the infection levels in adults at 36 months, in communities in the biannually treated arms. Because only children are treated, changes in adult prevalence are attributable to herd protection. The primary comparison will disregard the intended coverage target assignments and compare all villages using the Wilcoxon signed rank test (one-sample test on the differences, thus accounting for pairing). Supplemental analyses will (a) be conducted separately on the 90+% coverage arm, and (b) be conducted using a linear model with the outcome variable being the 36 month adult prevalence, and predictors being the baseline prevalence in adults, and coverage. Baseline prevalence in children may also be used as a predictor instead of baseline prevalence in adults.
  - We propose to compare chlamydia infection levels in adults (based on PCR taken from conjunctival swabs) at 36 months between the adult mass treatment arms and the children treated arms. The intent is to compare the herd effect (seen in the children treatment arms) with the direct effect. Specifically, we propose to use a linear model with 36 month adult prevalence as the outcome, baseline adult prevalence, and coverage as predictors.
  - We propose to examine the final prevalence (36 month) prevalence in children in a linear model for all study arms. We will look at high vs low coverage, and biannual-child vs annual-all age indicators as predictors (a factorial model). Baseline prevalence will be used as a covariate.

# **Chapter 17: Responsibilities of PRET Partners**

Chapter

17

_____________________________________________________________________

## 17.1 Overview

This trial provides an excellent opportunity to examine the impact of various combinations of coverage and frequency of administration of mass treatment of azithromycin in country situations of trachoma ranging from hyperendemicity to hypoendemicity. Each of the country sites has power to detect differences between their various treatment arms, but the power of this partnership lies in the uniformity of data collection that will permit comparisons across countries.

The partners have agreed to conduct the trial with some overall coordination as well as some autonomy for each country site. In this section we describe the responsibilities of the partners in this trial.

## 17.2 PRET Executive Committee

The PRET Executive Committee comprised of Dr Sheila West, Dr Thomas Lietman and Dr David Mabey and the Chair, Dr Thomas Quinn is responsible for oversight of the partnership. The committee will be charged with the following:

- Overall project review; review of progress at yearly meetings, review of data coordination and management at 6 monthly conference calls
- Receiving recommendations from the DSMC and implementation of recommendations
- Making recommendations for resource allocation. Johns Hopkins University will serve as the primary accounting body for the partnership.
- Review of quality assurance reports from the CC of each center, and the reports on laboratory and trachoma quality assurance, and make recommendations to the study site principal investigators.
- Serve as a writing committee to oversee the draft of manuscripts using data from more than one site

We have created specific objectives to meet the overall goal of this project, and activities and milestones that chart the successful completion of each objective. The committee will use these milestones to monitor the ongoing progress of our project, and report every six months to all partners. The main evaluation component of the antibiotic intervention part of PRET will be the following: The determination of a cost effective treatment coverage and frequency to achieve rapid elimination of ocular *C. trachomatis* infection and disease reduction, and the development of guidelines for “graduating” communities to maximize program resources.

.

## 17.3 Study Chairman’s Office

Dr West, as principal investigator, has overall responsibility for the PRET partnership, and for assuring the overall scientific conduct of the trial, with assistance from the partners. The Chairman’s office has specific responsibilities for the following:

- Obtaining the grant funding and sub-contract dispersal
- Liaison with the Bill and Melinda Gates Foundation
- Convening the Data and Safety Monitoring Committee, and working with the DSMC study chairman on recommendations to the Executive Committee
- Convening the Executive Committee meetings and teleconferences, with assistance from the Center for Global Health
- Manage correspondence between all partners and keep a repository of all reports
- Ensure that partners follow through on protocols in a timely fashion and execute all areas of research as indicated in the grant
- Together with partners, participate in all major study related publications from this project
- Ensure that all centers and country sites have received appropriate IRB clearance from institutions and countries.
- Coordinate with partners and The Center for Global Health to carry out Standardization Workshop
- Preparation of all reports to be submitted to the DSMC, and to the Gates Foundation, together with partners
- Receive sub-contract reports and be certain that sub contract funds are disbursed in a timely fashion.

## 17.4 Clinical Centers: Niger, Tanzania, and The Gambia

The clinical centers have day to day responsibility for hiring and training staff, carrying out the trial in their country study sites, collection and transmittal of data, data management (discussed below), and data analyses for the data from their country site. Specific responsibilities include the following:

- Ensure the execution of the study at each site per protocol
- Coordinate with the Study Chairman’s office in writing progress reports and budget accounting
- Maintain all ethical clearances for the study, including IRB renewals, and DSMC-related approvals for country specific activities
- In collaboration with JHU data coordination team, prepare all forms and documents necessary for fieldwork
- Participate in Standardization workshop; ensuring relevant country team members are present, trained, and certified. Each site must also train of team members for each data collection and mass treatment
- Arrange logistics and itineraries for traveling team members in country
- Purchase, maintain, and organize transport of all necessary study supplies to and within country
- Conduct data entry of data collected, ensure appropriate back up, and send data to data management team for each clinical site.
- Coordinate and oversee shipping of collected study samples per protocol and IATA regulations
- Coordinate and oversee sending of photographs per protocol to LSHTM for review
- Coordinate and oversee sending data as required to LSHTM for cost effectiveness component
- Coordinate and oversee sending of data as required to UCSF for modeling component
- Principal investigator for each clinical site will participate in Executive Committee Meetings, and teleconferences and DSMC meetings as needed.
- Prepare reports as required for quality assurance and monitoring purposes.
- Creation of manuscripts on data specific to each site, review through the Executive committee partners, and publication

## 17.5 Data Coordination Centers

### 17.5.1 The Data Coordination Center

The Dana Coordination Center at Johns Hopkins University has overall responsibility for the creation of PRET study forms to be used at each clinical site, for the data entry programs that will be used at each site, and the manuals for the data bases. They will construct the training in use of these forms, and certify at least one person from each site on use of the forms, data entry, and data backup. However, each clinical site has its own Coordinating Center with responsibility for data management and quality assurance of data, which will be reviewed by the Executive Committee.

Details of responsibilities for each Center are described below

#### 17.5.1.1 JHU

Ms. Beatriz Munoz and the JHU CC have responsibility for the following:

- Creation of PRET Study Forms
- Creation and maintenance of data entry programs for all sites
- Creation and maintenance of manuals for data entry programs
- Training program on use of forms and data entry programs at Standardization Workshop
- Create randomization scheme for villages, and for children within villages, monitor adherence to randomization
- Determine if villages meet stopping rules and issue stopping rule report after 6, 18, 30 month surveys.
- Monitor adherence to stopping rules
- Create and maintain database for Tanzanian data for the study
- Monitor receipt of data from the Tanzanian field office after each census, examination survey, and mass treatment
- Analyze and provide data when requested by co-investigators, and for DSMC meetings
- Appropriately back-up all data
- Develop and run data cleaning checks and quality assurance steps as part of data management; communicate with Tanzania field site to resolve data problems
- Monitor receipt of specimens at Chlamydia Laboratory, work with Tanzania field office to account for any missing specimens, receive data on specimen results from the laboratory, and integrate into the data base
- Provide data from Tanzania on timely basis to UCSF for modeling component, and to LSHTM for cost effectiveness component
- Create reports on Tanzania data as required for progress reports on data quality assurance
- Participate in quality assurance activities for trachoma grading and laboratory specimen processing as required

#### 17.5.1.2 LSHTM

Dr Robin Bailey and the LSHTM CC have responsibility for the following:

- Maintenance of data entry programs for Gambian sites
- Coordinate with JHU CC to implement study forms and data entry programs
- Training program on trachoma grading at Standardization Workshop
- Primary responsibility for Report on Trachoma grading quality assurance (see Below)
- Create randomization scheme for EAs, and for children within EAs, monitor adherence to randomization
- Determine if EAs meet stopping rules and issue stopping rule report after 6, 18, 30 month surveys.
- Monitor adherence to stopping rules
- Create and maintain database for The Gambian data for the study
- Monitor receipt of data from the Gambian field office after each census, examination survey, and mass treatment
- Analyze and provide data when requested by co-investigators, and for DSMC meetings
- Appropriately back-up all data
- Develop and run data cleaning checks and quality assurance steps as part of data management; communicate with The Gambian field site to resolve data problems
- Monitor receipt of specimens at Chlamydia Laboratory, work with Gambian field office to account for any missing specimens, receive data on specimen results from the laboratory, and integrate into the data base
- Provide data from the Gambia on timely basis to UCSF for modeling component, and to LSHTM for cost effectiveness component
- Create reports on Gambian data as required for progress reports on data quality assurance
- Coordinate with economist to create reports of cost component of analyses
- Ensure partners involvement in cost-effectiveness analyses and publications

#### 17.5.1.3 The University of California San Francisco

Dr Travis Porco and the UCSF CC have responsibility for the following:

- Maintenance of data entry programs for Nigerien sites
- Coordinate with JHU CC to implement study forms and data entry programs
- Training program on laboratory procedures at Standardization Workshop
- Primary responsibility for Report on Laboratory quality assurance
  - Conduct periodic testing of duplicate specimens from each laboratory for ongoing quality assurance, and feedback grades to CC at each site.
  - Create report on quality assurance of laboratory specimens for PRET executive committee with recommendations
- Create randomization scheme for STUs, and for children within STUs, monitor adherence to randomization
- Monitor adherence to yearly versus 6 monthly treatment s
- Create and maintain database for Nigerien data for the study
- Monitor receipt of data from Nigerien field office after each census, examination survey, and mass treatment
- Analyze and provide data when requested by co-investigators, and for DSMC meetings
- Appropriately back-up all data
- Develop and run data cleaning checks and quality assurance steps as part of data management; communicate with the Nigerien field site to resolve data problems
- Monitor receipt of specimens at Chlamydia Laboratory, work with Nigerien field office to account for any missing specimens, receive data on specimen results from the laboratory, and integrate into the data base
- Provide data from Niger on timely basis to LSHTM for cost effectiveness component
- Create reports on Niger data as required for progress reports on data quality assurance
- Conduct objective of creating models for trachoma elimination
- Ensure partners involvement in model data analyses and publications

### 17.5.2 Trachoma Grading Quality Assurance Center

Dr Robin Bailey at LSHTM has primary responsibilities for reviewing the consistency of clinical grading of trachoma at the three sites over time. The study site investigators are responsible for assuring their graders continue to be certified over time. The partnership considered using photographs to document trachoma, but determined that reading almost 5,000 photographs from each site for each survey point was untenable. Instead, the following steps will be undertaken to ensure that the grading of trachoma is standard across sites and does not drift over time.

1. Training and standardization of trachoma graders at the outset, in the standardization workshop. Each trachoma grader will go through a training that consists of slide sessions and field experience, until agreement for grading TF and TI is at least kappa 0.6, measured against Dr Bailey. Each trachoma grader must be certified for this trial.
2. At each six month survey round, the graders will submit 50 photographs of children they have graded to Dr Bailey, who will return his photographic grade to the CC for each site. The CC will compare Dr Bailey’s grades with the clinical grades, and issue a report to him on the agreement. The following steps will then take place
   1. If kappa is 0.6 or greater, then certification is maintained.
   2. If kappa <.6 for trachoma, then the CC for that site will issue a report to that site for that grader, the certification is set to provisional, and the study site investigators are responsible for assigning a certified grader to work with the provisional grader to achieve agreement at kappa at least 0.6 in the field.
      1. At the next survey round, the provisional grader again sends 50 photographs of grading. If the agreement is now 0.6 or greater, certification is restored.
      2. If agreement is <0.6, with Dr Bailey’s, then the grader is notified by the CC that grading must stop, and certification is rescinded. The study site investigator is responsible for making sure the grader goes through a whole re-training process, submitting a set of 50 photographs until agreement is again acceptable.
3. The Executive committee will review, every six months, the certification status of all graders, as provided by the data coordinating centers of each clinical site. Data on continued certification will also be presented to the DSMC as part of quality assurance monitoring.

### 17.5.3 Laboratory Quality Assurance Center

Dr. Thomas Lietman at UCSF has primary responsibility for reviewing the consistency of each of the laboratories in this study for determining evidence of infection using Amplicor. The directors of each laboratory, Dr. Martin Holland, Dr. Thomas Quinn, and Dr. Julius Schachter, will have responsibility for maintaining the quality control for their laboratories, and submitting specimens once per year for quality assurance purposes. The quality control procedures are as follows:

- Within each laboratory, standard quality assurance procedures are followed, that permit each laboratory to maintain their CLIA certification, and allow the CDC to grant permission to import specimens from overseas.
- Each test run for determining positivity contains a *C. trachomatis* (+) control and a *C. trachomatis* (-) control is included in each test run of the COBAS AMPLICOR™ CT/NG. To test the effect of sample processing, a known positive sample is processed and tested in each test run. (This control is helpful when testing large numbers of negative samples.). An internal control intended to identify specimens that contain polymerase inhibitor is run routinely on each sample. The internal control helps identify false negative results.
- Once per year, a set of five positive and five negative specimens from each laboratory will be re-aliquoted and half the sample will be sent to Dr. Lietman’s laboratory, masked as to the findings from the original lab. Dr. Lietman’s lab will re-run the results, as will the lab of origin for confirmation of results, thus providing assurances of similar procedures across laboratories. There must be at least 80% concordance of results between the laboratory and Dr. Lietman’s lab. The following steps will be followed:
- Dr. Lietman and the lab of origin will send their results to the study site CC.
- The CC will send a report of the concordance, with ID numbers of non-concordant results, to the lab of origin and to Dr. Lietman.
- If concordance is at least 80%, the CC will issue a one year quality assurance certificate to the lab and to Dr. Lietman for his records, and to the Executive committee.
  - If concordance is less, the CC will issue a provisional notice to the lab chief and Dr. Lietman, who must work together to resolve the difference, including re-sending specimens until concordance is reached. Once concordance is reached, the quality assurance certificate is again issued to the lab and to Dr. Lietman for his records, and to the Executive Committee. Note that some of the discrepancy may be due to issues with shipping, resulting in loss of positivity of specimens. To the extent possible the re-aliquoted specimens in the laboratory will be stored under conditions that mimic the shipping and re-storage so that conditions of the specimens are similar.

The Executive Committee will review yearly the quality assurance certificates for the laboratories. Data on continued quality assurance will be presented to the DSMC.

There is also the possibility of field contamination, which is checked by using the “blue air” specimens. Each laboratory will process these controls masked as to their status as controls. The CC for each site is responsible for reporting the positivity of the negative field controls to the study site investigators. If the rate is above 1%, then the investigators must take remedial action to correct field procedures to remove contamination. Data on filed contamination results will be presented to the DSMC for each site by the CC.

# **Chapter 18: Data and Safety Monitoring Committee and Contact Information**

Chapter

18

## 18.1 Charter of Data and Safety Monitoring Committee

This charter is for the Data and Safety Monitoring Committee (DSMC) of the Partnership for Rapid Elimination of Trachoma (PRET) trials on antibiotics for trachoma. The following table provides an overview of the trial.

The Charter will define the Terms of Reference for the DSMC, its membership, its relationship with other trial components, and the purpose and timing of its meetings. The Charter will also provide the procedures for ensuring confidentiality and proper communication, the monitoring guidelines to be implemented by the DSMC, and outline of the content of the Reports that will be provided to the DSMC

## 18.2 Terms of Reference

1. The Data and Safety Monitoring Committee for the PRET trials are charged with oversight of the quality of the data collected, and the safety and efficacy of the antibiotic approach proposed in the trial. The committee is guided by the principles set out in the National Eye Institute Guidelines for Data and Safety Monitoring of Clinical Trials.
2. As part of its responsibilities for oversight of safety issues, the Committee shall review reports on adverse events at each meeting. If any member expresses to the chair of the DSMC committee concerns for safety issues in the interim, the chair may request a meeting or telephone conference call of a quorum of the members. If the DSMC believes that there is evidence that warrants a change in the procedures of the clinical trial, the Committee will be responsible for drafting a report of their recommendations to the Executive Committee of PRET and to the IRBs as relevant of Johns Hopkins University, University of California at San Francisco, and London School of Hygiene and Tropical Medicine. If there is no concern, the Committee will also issue a report stating they have no concerns.
3. The DSMC shall meet together at least once per year, and more frequently by telephone if deemed necessary by the members or chair of the committee. The meeting shall be for the purposes of reviewing the accumulated data on the quality of the information collected, the safety of the trial procedures, and the efficacy of the treatment arms. The committee shall issue a summary report, which will be forwarded to the Executive Committee of PRET and each Institutional Review Board.
4. The locations of the trials are in rural Africa. Social, cultural, or political exigencies may arise that may affect the trial or call into question some of the protocols of the trial. The DSMC has accepted the responsibility to serve as a review body for such matters, if requested by members of the DSMC or members of the study team. The members of the DSMC may request a review by the full committee of any events arising from the study which, in the opinion of the committee member, is a serious issue or one in which a response to a government may be necessary. The Study Chair will arrange a conference call as expeditiously as possible with a quorum of members. For this purpose, a quorum is considered three of the four members, with the member from Africa required and the DSMC chair considered a member. The call must include the Study Principal Investigator and the relevant principal investigator from the country (or countries) involved. The committee will recommend a course of action to the Study Principal Investigator regarding any such events. If the study leadership does not concur with the DSMC's recommendation, it will be the responsibility of the study leadership and DSMC Chair to reach a mutually acceptable decision. A minority report will be issued from this group.

## 18.3 DSMC Key Responsibilities

The DSMC will be responsible for carrying out the following responsibilities:

- Reviewing and approving the trial protocol and plans for data and safety monitoring
- Assessing data quality, including completeness
- Monitoring recruitment and losses to follow-up
- Monitoring compliance with the treatment protocol by villages and investigators
- Monitoring evidence for intervention differences in the main outcomes
- Monitoring the safety of interventions (e.g., adverse events)
- Deciding whether to recommend that the trial continues as planned or whether treatment should be terminated in some treatment groups
- Reviewing proposed major modifications to the study prior to their implementation (e.g., increasing target sample size, dropping an arm based on other trial outcomes or toxicity results).
- Suggesting additional data analyses

## 18.4 DSMC Membership

Members of the DSMC are appointed by the Executive Committee of PRET. The DSMC is an independent multidisciplinary group consisting of the following four members:

Dr Douglas Jabs: Chair. Ophthalmologist

Dr Antoinette Darville: Pediatric Infectious disease

Dr Grace Saguti: Ophthalmologist

Dr Maureen Maguire: Biostatistician and clinical trialist

The DSMC voting membership includes all four members mentioned above. Non-voting members include the members of the PRET Executive committee (Dr Thomas Quinn, Dr Thomas Lietman, Dr David Mabey, and Dr Sheila West), and members of the PRET team as required: Dr Emily Gower, Dr Shannath Merbs, Dr Robin Bailey, Dr Travis Porco, and Ms Beatriz Munoz.

### 18.4.1 Conflicts of Interest

The DSMC membership is restricted to individuals free of apparent significant conflicts of interest. The source of these conflicts may be financial, scientific or regulatory in nature. Thus, DSMC members may not be involved in PRET, have a vested interest in its outcome, have close personal or professional ties to a PRET investigator, or have financial investments in Pfizer. If at any time a DSMC member perceives that he/she or another member of the Committee has a potential conflict of interest, he/she is obligated to bring the issue to the attention of the full DSMC for open discussion and resolution. Any DSMC members who develop significant conflicts of interest during the course of trial should resign from the DSMC. The PRET Executive Committee will promptly appoint a replacement.

DSMC members will complete a conflict of interest disclosure form in advance of the first meeting. Competing interests should be disclosed by all DSMC members. The completed forms will be reviewed by the DSMC Chair. If a significant conflict of interest is noted, the DSMC Chair will determine if that a member has a conflict of interest that would compromise their ability to serve on the DSMC. If he recommends resignation, the member will be replaced.

The DSMC membership is to be constituted for the duration of the PRET trials. Before the publication of PRET trial primary results, the DSMC members should not discuss issues from their involvement in the trial.

### 18.4.2 Relationships

The DSMC is appointed by the Executive Committee of PRET and is advisory to the Executive Committee. The DSMC will not make decisions about the trials but rather advise the Executive Committee of PRET as to the data quality and safety of the interventions throughout the trial.

## 18.5 DSMC Meetings

The DSMC will meet once per year, at the start of the project, and once per year thereafter when there is data to review. Additional DSMC teleconferences will be scheduled as the Chair recommends. The agenda will be prepared by the DSMC chair in consultation with the study investigators.

At least five days before the DSMC the Study Principal Investigator will send to the DSMC members a binder of study materials via Federal Express to allow the members’ time to review the material before the meeting. The binders will include a set of data reporting tables, shown in Appendix 3. In keeping with confidentiality, the DSMC members will return the binder of materials to the Principal Investigator at the close of the meeting. The Study Principal Investigator and her team will be responsible for the preparation of meeting materials and all DSMC meeting arrangements.

Open sessions of the DSMC meeting may be attended by DSMC voting members, and the non-voting members, and key study team members from each of the clinical sites. Other members of the PRET study team may also be asked by the DSMC chair to attend.

Closed sessions of the DSMC will be attended only by the voting DSMC members, the Executive committee, and the three study statisticians. The closed session may be called when the DSMC reviews the safety and impact data unmasked as to random assignment. At the discretion of the DSMC chair, the committee can meet in Executive session with only the four voting members present to discuss issues and take formal votes as needed.

Minutes will be prepared by the Study Principal Investigator for the antibiotic trial, and the study project director for the surgery trial. Draft minutes will be combined and distributed to the DSMC chair for review and changes before distribution to the entire committee. The minutes shall summarize all recommendations.

Members must make every effort to attend DSMC meetings. If a member fails to make a meeting, it should be ensured that the member makes the next meeting. If a member fails to make the second meeting, the member will be replaced.

### 18.5.1 Protocol Changes and Ancillary Studies

The DSMC will meet in advance of the start of the trials. The DSMC is charged with reviewing and approving the protocols. The DSMC may review patient oral consent forms, but the institutional and country IRBS have primary responsibility for approval of these forms.

The DSMC will be informed in advance of major study protocol changes. DSMC concurrence will be sought on all substantive recommendations for changes prior to their implementation. Ancillary studies affecting the antibiotic trial in all countries will be brought forward to the DSMC for their information. The DSMC will review them for possible impact on the main trials. The DSMC will not make recommendations regarding whether the studies may or may not proceed, which is the province of the Executive committee.

### 18.5.2 Recommendations

DSMC recommendations will be provided to the study leadership and to the Executive Committee of PRET. If the recommendations involve participant safety, the study principal investigator, in collaboration with the site investigators, will act to implement the change as expeditiously as possible. However, if the study leadership does not concur with the DSMC recommendations, it will be the responsibility of the study leadership and DSMC Chair to reach a mutually acceptable decision for implementation, and a report issued to other DSMC members.

18.6 DSMC Contact Information

| **Table 5:DSMC Contact Information** |
| --- |
| Antoinette Darville, M.D.  University of Pittsburgh Physicians Pediatric Infectious Diseases  Keystone Building  3520 Fifth Avenue  Pittsburgh, PA 15213  Phone: 412-692-7885  email: [Toni.Darville@chp.edu](mailto:Toni.Darville@chp.edu) |
| Douglas Jabs, M.D., M.B.A.  Chairman, Department of Ophthalmology  Mt Sinai School of Medicine  One Gustave L. Levy Place, Box 1183  New York, NY 10029-6574  Phone: 212-241-6752  Fax: 212-241-5764  e-mail: [douglas.jabs@mssm.edu](mailto:douglas.jabs@mssm.edu) |
| Maureen Maguire, Ph.D.  University of Pennsylvania  3535 Market Street, Suite 700  Philadelphia, PA 19104-3309  Phone: 215-615-1501  Fax: 215-615-1531  e-mail: [maguirem@mail.med.upenn.edu](mailto:maguirem@mail.med.upenn.edu) |
| Dr. Grace E. B. Saguti  National Professional Officer- Disease Prevention & Control  World Health Organization  P.O. Box 9292, Dar-es-Salaam, Tanzania  Tel: +255 22 211718/2113005  Mob:+255 754 287875  Fax: +255 22 2113180  GPN: 37429  E-mail: [sagutig@tz.afro.who.int](mailto:sagutig@tz.afro.who.int)  [gracejengo@yahoo.co.uk](mailto:gracejengo@yahoo.co.uk) |

# **Chapter 19: Timeline**

Chapter

19

# **Figure 4: PRET Timeline - All Locations**

# **Figure 5: PRET Timeline - Niger**

# Figure 6: PRET Timeline - **Tanzania**

# **Figure 7: PRET Timeline - The Gambia**

Chapter

20

# **Chapter 20: Overall Project**

# **Figure 8: Program for the Rapid Elimination of Trachoma Overview**

Appendix

1

# **Appendix 1: General Study Forms**

# **Appendix 2: Informed Consent Forms**

# **Appendix 3: DSMC Tables**

1. [Frick KD](http://www.ncbi.nlm.nih.gov/sites/entrez?Db=pubmed&Cmd=Search&Term="Frick KD"%5BAuthor%5D&itool=EntrezSystem2.PEntrez.Pubmed.Pubmed_ResultsPanel.Pubmed_RVAbstractPlus), [Basilion EV](http://www.ncbi.nlm.nih.gov/sites/entrez?Db=pubmed&Cmd=Search&Term="Basilion EV"%5BAuthor%5D&itool=EntrezSystem2.PEntrez.Pubmed.Pubmed_ResultsPanel.Pubmed_RVAbstractPlus), [Hanson CL](http://www.ncbi.nlm.nih.gov/sites/entrez?Db=pubmed&Cmd=Search&Term="Hanson CL"%5BAuthor%5D&itool=EntrezSystem2.PEntrez.Pubmed.Pubmed_ResultsPanel.Pubmed_RVAbstractPlus), [Colchero MA](http://www.ncbi.nlm.nih.gov/sites/entrez?Db=pubmed&Cmd=Search&Term="Colchero MA"%5BAuthor%5D&itool=EntrezSystem2.PEntrez.Pubmed.Pubmed_ResultsPanel.Pubmed_RVAbstractPlus) Estimating the burden and economic impact of trachomatous visual loss. [Ophthalmic Epidemiol.](javascript:AL_get(this, 'jour', 'Ophthalmic Epidemiol.');) 2003 (2):121-32 [↑](#footnote-ref-2)
2. [Baltussen RM](http://www.ncbi.nlm.nih.gov/sites/entrez?Db=pubmed&Cmd=Search&Term="Baltussen RM"%5BAuthor%5D&itool=EntrezSystem2.PEntrez.Pubmed.Pubmed_ResultsPanel.Pubmed_RVAbstractPlus), [Sylla M](http://www.ncbi.nlm.nih.gov/sites/entrez?Db=pubmed&Cmd=Search&Term="Sylla M"%5BAuthor%5D&itool=EntrezSystem2.PEntrez.Pubmed.Pubmed_ResultsPanel.Pubmed_RVAbstractPlus), [Frick KD](http://www.ncbi.nlm.nih.gov/sites/entrez?Db=pubmed&Cmd=Search&Term="Frick KD"%5BAuthor%5D&itool=EntrezSystem2.PEntrez.Pubmed.Pubmed_ResultsPanel.Pubmed_RVAbstractPlus), [Mariotti SP](http://www.ncbi.nlm.nih.gov/sites/entrez?Db=pubmed&Cmd=Search&Term="Mariotti SP"%5BAuthor%5D&itool=EntrezSystem2.PEntrez.Pubmed.Pubmed_ResultsPanel.Pubmed_RVAbstractPlus) Cost-effectiveness of trachoma control in seven world regions. [Ophthalmic Epidemiol.](javascript:AL_get(this, 'jour', 'Ophthalmic Epidemiol.');) 2005;:91-101 [↑](#footnote-ref-3)
3. [Solomon AW, Holland MJ, Alexander ND, Massae PA, Aguirre A, Natividad-Sancho A, Molina S, Safari S, Shao JF, Courtright P, Peeling RW, West SK, Bailey RL, Foster A, Mabey DC.](http://www.ncbi.nlm.nih.gov/sites/entrez?Db=pubmed&Cmd=ShowDetailView&TermToSearch=15525721)Mass treatment with single-dose azithromycin for trachoma.N Engl J Med. 2004;351:1962-71 [↑](#footnote-ref-4)
4. West SK, Munoz B, Mkocha H, Holland MJ, Solomon AW, Foster A, Bailey RL, Mabey DCW. Infection with *Chlamydia trachomatis* after mass treatment of a trachoma hyperendemic community in Tanzania: a longitudinal study. Lancet 2005;366:1296-1300 [↑](#footnote-ref-5)
5. Report of the Eighth Meeting of the WHO Alliance for the Global Elimination of blinding Trachoma. *Report of a WHO Working Group.* 2004; World Health Organization Geneva Switzerland. WHO/PBD/GET/04.2 [↑](#footnote-ref-6)
6. Chaudhary MA, Moulton LH. A SAS Macro for constrained randomization of group-randomized designs. Comp Methods and programs in BioMed. 83:2006:205-210 [↑](#footnote-ref-7)
7. Chow SC, Shao J, Wang H. Sample size calculations in clinical research. Boca Raton: Taylor and Francis, 2003 [↑](#footnote-ref-8)
8. Melese M, Alemayehu W, Lakew T, Yi E, House J, Chidambaram JD, Zhou Z, Cevallos V, Ray K, Hong KC, Porco TC, Phan I, Zaidi A, Gaynor BD, Whitcher JP, Lietman TM. Comparison of annual and biannual mass antibiotic administration for elimination of infectious trachoma. JAMA 299(7):778-84, 2008 [↑](#footnote-ref-9)
9. Chow SC, Liu JP. Design and Analysis of Clinical Trials, 2d ed. New York, John Wiley, 2004 [↑](#footnote-ref-10)
10. Snapinn SM. 2000. Noninferiority trials. Current Controlled Trials in Cardiovascular Medicine 1:19-21 [↑](#footnote-ref-11)
11. Chow SC, Shao J, Wang H. Sample size calculations in clinical research. Boca Raton: Taylor and Francis, 2003 [↑](#footnote-ref-12)
12. Feiveson AH. 2002. Power by simulation. STATA Journal 2:107-124 [↑](#footnote-ref-13)
13. Holford NHG, Kimko HC, Monteleone JPR, Peck CC. 2000. Simulation of Clinical Trials. Annual Review of Pharmacology and Toxicology 40:209-234 [↑](#footnote-ref-14)
14. Chow SC, Liu JP. Design and Analysis of Clinical Trials, 2d ed. New York, John Wiley, 2004 [↑](#footnote-ref-15)
15. [Solomon AW, Holland MJ, Alexander ND, Massae PA, Aguirre A, Natividad-Sancho A, Molina S, Safari S, Shao JF, Courtright P, Peeling RW, West SK, Bailey RL, Foster A, Mabey DC.](http://www.ncbi.nlm.nih.gov/sites/entrez?Db=pubmed&Cmd=ShowDetailView&TermToSearch=15525721)Mass treatment with single-dose azithromycin for trachoma.N Engl J Med. 2004;351:1962-71 [↑](#footnote-ref-16)
16. West SK, Munoz B, Mkocha H, Holland MJ, Solomon AW, Foster A, Bailey RL, Mabey DCW. Infection with *Chlamydia trachomatis* after mass treatment of a trachoma hyperendemic community in Tanzania: a longitudinal study. Lancet 2005;366:1296-1300 [↑](#footnote-ref-17)
17. Burton MJ, Holland MJ, Makalo P et al. Re-mergence of Chlamydia trachomatis infection after mass antibiotic treatment of a trachoma endemic Gambian village. A longitudinal Study. Lancet 2005; 365:1321-8 [↑](#footnote-ref-18)
18. Thylefors B, Dawson CR, Jones BR, West SK, Taylor HR. A simple system for the assessment of trachoma and its complications. *Bull World Health Organ*. 1987; 65(4):477-83. [↑](#footnote-ref-19)
19. Schachter J, West SK, mabey D, et al. Azithromycin for Trachoma Control. Lancet 1999;354:630-5 [↑](#footnote-ref-20)
20. West SK, West ES, Alemayehu W, Melese M, Munoz B, Imeru A, Worku A, Gaydos C, Meinert CL, Quinn T. Single-dose Azithromycin prevents trichiasis [↑](#footnote-ref-21)
21. Gaynor B, Holbrook KA, Witcher JP, et al. Community Treatment with Azithromycin for trachoma is not associated with antibiotic resistant S. Pneumoniae at One year. Br J Ophth. 2003;87:147-8 [↑](#footnote-ref-22)
22. Bailey N, 1957. The Mathematical Theory of Epidemics. London: Griffin [↑](#footnote-ref-23)
23. Bartlett MS, 1956. Deterministic and Stochastic Models for Re-current Epidemics. Berkeley, CA: Proceedings of the Third Berkeley Symposium on Mathematical Statistics and Probability 4: 81−110. [↑](#footnote-ref-24)
24. Lee HL, Pierskalla WP. Mass screening models for contagious diseases with no latent period. Oper Res. 1988 Nov-Dec; 36(6):917-28. [↑](#footnote-ref-25)
